# Developing a mobile RNA delivery system for grafting trait improvement

**DOI:** 10.1038/s44319-026-00819-z

**Published:** 2026-06-10

**Authors:** Yiran Tao, Jiayu Zhang, Yuyang Zhang, Miaomiao Lei, Xuan Dong, Qicong Li, Jiuyuan Bai, Yun Zhao

**Affiliations:** Key Laboratory of Bio-Resource and Eco-Environment of Ministry of Education, College of Life Sciences, Sichuan University - Wangjiang Campus, Chengdu, China

**Keywords:** Plant Biology, RNA Biology

## Abstract

Systemic RNA movement offers a route for non-transgenic trait improvement by grafting, but progress has been limited by the lack of reliable tools to identify mobile RNA elements. Here, we report a fluorescence-aptamer-based live-imaging platform for screening mobile RNAs and mapping mobility elements. In a transient screening assay, live imaging of 100 RNA-seq-predicted candidates detects three reproducibly mobile mRNAs, *CAT3*, *CK1*, and *GAI*, under our assay conditions, with *GAI* mRNA exhibiting the highest bidirectional mobility. Truncation analysis defines two independent 30-nt cis-elements, GME1 in the coding region and GME2 in the 3′UTR, with GME2 mediating a higher transport rate and mobility rate. A tandem 2×GME2 cassette in the 3′UTR functions as a high-capacity RNA delivery module and, in our transient assay, outperforms the tRNA-like sequence motif (TLS; tRNA^Met^) construct tested here. Using 2×GME2, we deliver three otherwise non-mobile mRNAs across graft junctions and detect associated molecular or phenotypic effects in recipient tissues. Together, these findings establish a live-imaging-guided pipeline for mobile RNA validation and identify 2×GME2 as a non-viral RNA delivery element for graft-mediated trait modification.

## Synopsis

This study identifies and validates a 2×GME2 cassette as a novel non-viral delivery module for functional mRNAs, through which stress-related trait improvement can be achieved in grafted plants.

- Two mobility elements in *GAI* mRNA are identified using the 3WJ-4×Bro RNA imaging system in living plants.
- GME2 is engineered into a tandem 2×GME2 RNA delivery cassette with high capacity.
- The 2×GME2 delivery cassette enables the delivery of otherwise non-mobile functional mRNAs in grafted plants and confers tolerance to stress.

## Abstract (web summary)

This study identifies and validates a 2×GME2 cassette as a novel non-viral delivery module for functional mRNAs, through which stress-related trait improvement can be achieved in grafted plants.

## Graphical abstract

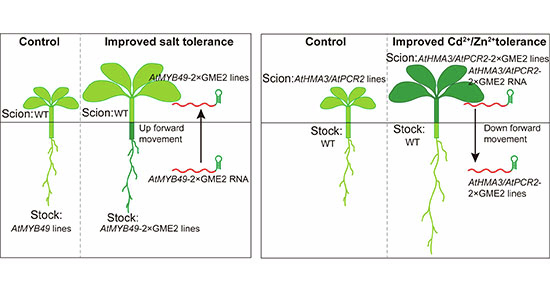

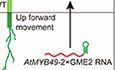

## Introduction

Grafting is a widely used asexual propagation technique in agriculture and forestry that enables the combination of distinct rootstock and scion genotypes to enhance tolerance to biotic and abiotic stresses (Wang, 2011; Wang et al, 2024; Qin et al, 2014), overcome soil-borne diseases (Panth et al, 2020), and facilitate the production of elite cultivars (Bletsos, 2006; Bakirbas et al, 2023; Zhang et al, 2002). However, current grafting practices largely rely on the empirical selection of rootstock-scion combinations, and the outcomes of trait improvement remain unpredictable due to the absence of tools that can direct information flow across the graft union (Loupit et al, 2023; Wang et al, 2024). There is an urgent need for strategies that can convert grafting from a largely empirical practice into a precise technology for directional trait improvement (Albacete et al, 2015).

In plants, long-distance movement of hormones (Akhiyarova et al, 2024), peptides (Carbonnel et al, 2023), proteins (Colleoni et al, 2024), and RNAs (Kitagawa et al, 2024; Bakirbas et al, 2023) through the phloem allows plants to coordinate development and environmental responses across organs. Among these signals, mobile mRNAs have attracted particular attention as informational molecules capable of conveying genetic information between tissues and mediating long-distance regulation (Zhang et al, 2025). Classical examples include *KNOTTED1* (Lucas et al, 1995), *GIBBERELLIC ACID-INSENSITIVE* (*GAI*) (Haywood et al, 2005; Huang and Yu, 2009), *CAT3* (Thieme et al, 2015), *CAX3* (Hao et al, 2022), *CK1* (Thieme et al, 2015; Zhang et al, 2016), *StBEL5* (Cho et al, 2015; Banerjee et al, 2006) and *FLOWERING LOCUS T* (*FT*) (Li et al, 2011; Li et al, 2009), which move over long distances and modulate leaf development, nutrient homeostasis, tuberization and flowering (Cho et al, 2015; Hao et al, 2022; Haywood et al, 2005). Mobile RNA motifs therefore showed potential as delivery vehicles to transport gene-editing components or functional RNAs from transgenic donor tissues into non-transgenic recipients.

To date, a limited number of sequence-defined mobile RNA elements have been described, including TLS-related elements (Zhang et al, 2016), and endogenous cis-acting mobility determinants such as those identified in *FT* (Li et al, 2009), while additional studies have shown that RNA modifications, including m^5^C and m^6^A, can also contribute to the mobility of specific transcripts (Li et al, 2025; Yang et al, 2019). Although proof-of-concept studies have demonstrated grafting-induced, transgene-free genome editing using TLS- based systems (Yang et al, 2023) or virus-based systems (Kang et al, 2025),virus-based approaches still face constraints related to cargo capacity and biosafety (Oh et al, 2025; Kang et al, 2025), while the broader efficiency and applicability of non-viral mobile RNA delivery systems remain to be improved.

A major bottleneck underlying these limitations is the lack of robust methodologies for identifying bona fide long-distance mobile RNAs and their minimal transport elements. Most candidate mobile mRNAs have been inferred from grafting-based transcriptome comparisons between scions and rootstocks, but a recent re-analysis of short-read RNA-seq and SNP-based graft-mobile mRNA datasets revealed a high false-positive rate and suggested that only a subset of predicted candidates are supported as bona fide long-distance mobile transcripts (Paajanen et al, 2025). Existing validation approaches—such as RT–PCR detection of tagged transcripts or GFP-based reporter systems (Huang and Yu, 2009; Li et al, 2025)—usually provide limited spatial resolution and may perturb RNA conformation or trafficking due to large protein tags (Xiao et al, 2024; Itzkovitz and van Oudenaarden, 2011). As a result, the sequence features that encode RNA mobility, and their utility as modular delivery elements, remain poorly characterized (Feng et al, 2024).

To overcome these challenges, sensitive, minimally perturbing tools are required to visualize mobile RNAs in vivo and to map their mobility elements at high resolution. Such tools would not only clarify the true extent of systemic mRNA signaling, but also enable the engineering of RNA delivery systems that can transport large functional RNA cargos across graft unions, without reliance on viral vectors or genomic integration. This resulting capability is particularly attractive for crops that are recalcitrant to genetic transformation, as it could support non-transgenic precision breeding via graft-mediated RNA delivery (Zhang et al, 2016; Yang et al, 2023).

In this study, we employ a protein-independent fluorescent RNA aptamer, 3WJ-4×Bro (Bai et al, 2020), to visualize long-distance mRNA transport at the whole-plant scale. Using a 3WJ-4×Bro-based screening framework, we screened 100 RNA-seq-predicted mobile mRNA candidates and found that only three of them showed reproducible long-distance mobility under our assay conditions (*CAT3* (Thieme et al, 2015), *CK1* (Thieme et al, 2015; Yang et al, 2023), and *GAI* (Huang and Yu, 2009)). Guided by live imaging and truncation analysis of *GAI* mRNA, we define two independent 30-nt mobility elements, GME1 in the coding region and GME2 in the 3′ untranslated region, and show that GME2 possesses superior transport rate and efficiency. Building on this element, we construct a 2×GME2-based RNA delivery system that enables high-capacity, long-distance transport of diverse target mRNAs, including non-mobile functional transcripts. Finally, we demonstrate that 2×GME2-mediated delivery of *AtMYB49* (Zhang et al, 2020), *AtHMA3* (Morel et al, 2009), and *AtPCR2* (Song et al, 2010) mRNAs across graft unions enhances salt and heavy metal (Cd and Zn) tolerance in grafted *Arabidopsis thaliana* (*A. thaliana*) plants, illustrating a generalizable strategy for RNA-based, transgene-free trait improvement through grafting.

## Results

### 3WJ-4×Bro reports long-distance mRNA movement in intact plants

**Figure 1:**
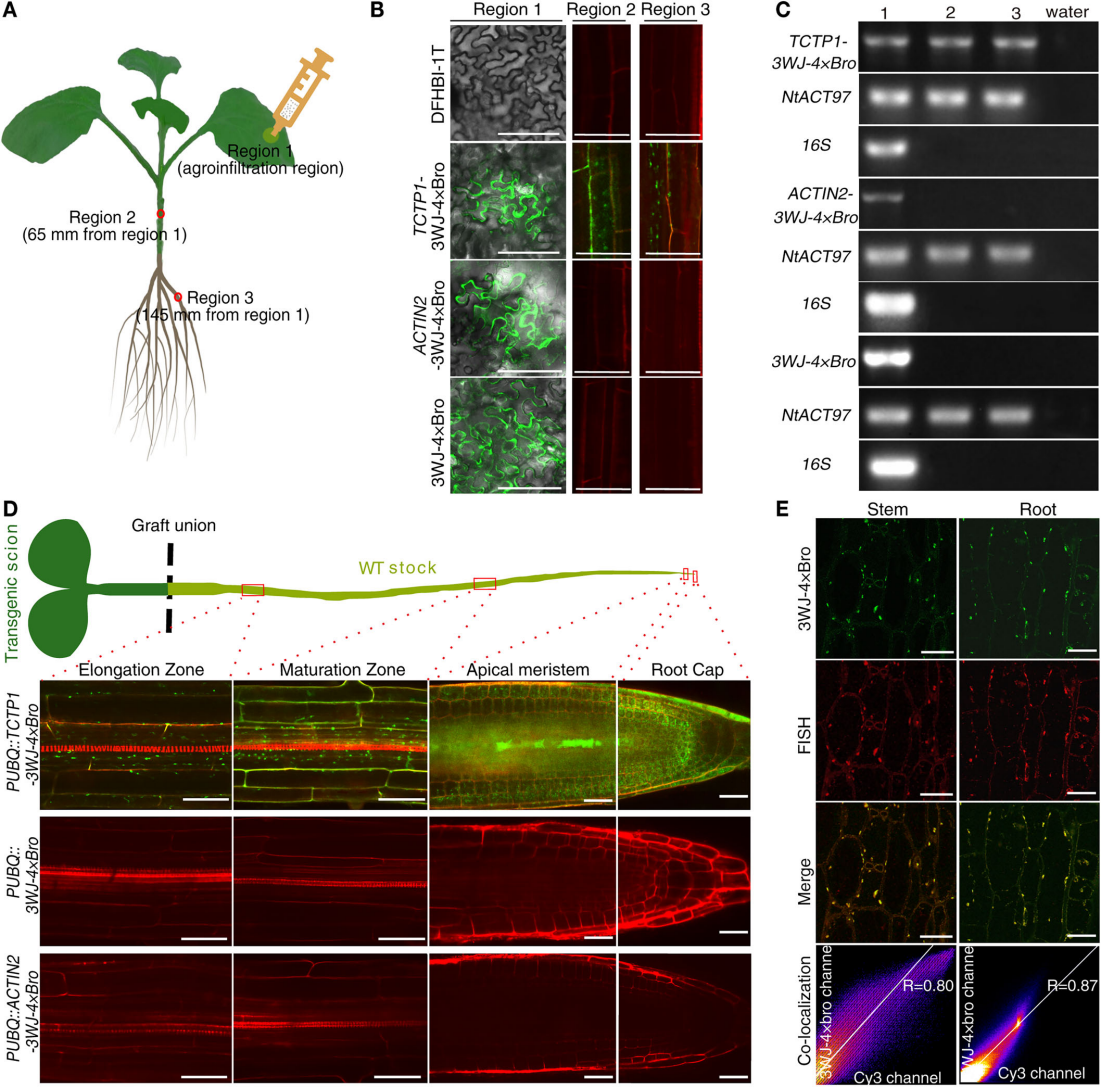
3WJ-4×Bro enables visualization of long-distance mRNA movement in intact plants. (**A**) Schematic of the experimental design in *N. benthamiana*. Region 1 corresponds to the agroinfiltrated leaf area, region 2 to the stem phloem 65 mm from the infiltration site, and region 3 to the root phloem 145 mm from the infiltration site. (**B**) Confocal images showing 3WJ-4×Bro fluorescence in infiltrated leaves and distal stem and root tissues after transient expression of *TCTP1*-, *ACTIN2*-, or aptamer-only constructs. Cell boundaries are indicated by PI (propidium iodide) staining (red). Scale bars, 100 μm (leaf), 20 μm (stem), 25 μm (root). (**C**) RT–PCR detection of 3WJ-4×Bro-tagged RNA and Agrobacterium 16S rDNA in region 1–3 samples. Lane 1, region 1; lane 2, region 2; lane 3, region 3. *NtACT97* served as a reference gene. (**D**) Representative longitudinal optical section images of root showing distribution of 3WJ-4×Bro-tagged RNAs in different root regions at 14 days post-grafting (> 2 independent transgenic lines per construct, each *n* > 10 plants). Cell boundaries are indicated by PI staining (red). Scale bars, 25  μm. (**E**) Co-localization of 3WJ-4×Bro (green) and Cy3-labeled FISH probe (red) detecting *TCTP1* mRNA in stems and roots of *N. benthamiana*. Scale bars, 50 μm (stem), 50  μm (root). Pearson’s co-localization coefficients are indicated in the panels. Source data are available online for this figure.

**Figure EV1:**
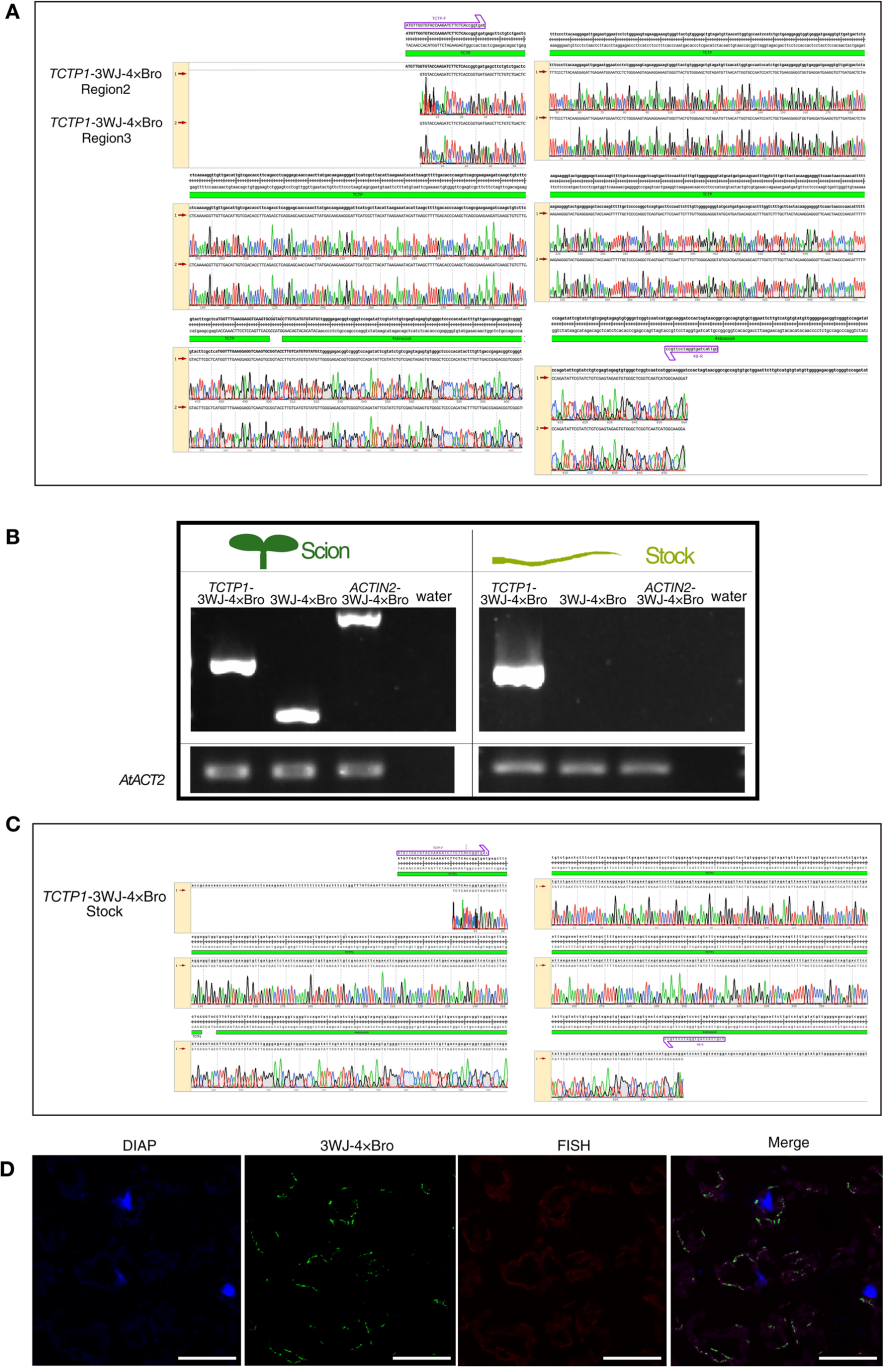
3WJ-4×Bro enables imaging various mobile mRNAs. (**A**) Sanger sequencing validation of *TCTP1-*3WJ-4×Bro in regions 2 and 3. (**B**) The RT–PCR analysis confirmed the presence of 3WJ-4×Bro-tagged RNA in root phloem. Samples were collected 1.5 cm below to the graft junction. The reference gene *AtACT2* was utilized in the analysis. (**C**) Sanger sequencing validation *TCTP1-3WJ-4×Bro* in wild-type (WT) stocks. (**D**) Representative confocal images of mock-infiltrated *N. benthamiana* leaves transiently expressing 3WJ-4×Bro alone. Samples were collected at 56 h post-infiltration (hpi). Scale bar, 50 μm.

To establish a tool for visualizing long-distance RNA movement in planta, we first evaluated whether the previously reported 3WJ-4×Bro fluorescent RNA aptamer (Bai et al, 2020) could be used to trace mobile mRNAs at the whole-plant level. We fused 3WJ-4×Bro to a known mobile mRNA, *TCTP1* (At3G16640) (Yang et al, 2019), and to a control mRNA, *ACTIN2* (AT3G18780) (Thieme et al, 2015) and transiently expressed *TCTP1*-3WJ-4×Bro, *ACTIN2*-3WJ-4×Bro, or 3WJ-4×Bro alone in leaf epidermal cells of *Nicotiana benthamiana* (*N. benthamiana*) via Agrobacterium infiltration (Fig. 1A). At 48 h post-inoculation (hpi), confocal imaging confirmed that all three constructs accumulated in the infiltrated region (region 1; Fig. 1B). At 56 hpi, however, strong fluorescence was detected in distal stem and root tissues (regions 2 and 3) only in plants expressing *TCTP1*-3WJ-4×Bro, whereas no signal was detected in plants expressing *ACTIN2*-3WJ-4×Bro or 3WJ-4×Bro alone (Fig. 1B). RT–PCR followed by Sanger sequencing verified the presence of *TCTP1*-3WJ-4×Bro transcripts, but not *ACTIN2*-3WJ-4×Bro or 3WJ-4×Bro, in RNA extracted from regions 2 and 3 (Figs. 1C and  EV1A). Moreover, no *Agrobacterium* 16S rRNA fragment was detected in these distal tissues (Fig. 1C), supporting the interpretation that the distal fluorescence originated from mobile RNA rather than local bacterial contamination in this transient assay.

We next assessed whether 3WJ-4×Bro could report long-distance RNA movement in a grafting system. Transgenic *A. thaliana* lines expressing 3WJ-4×Bro, *ACTIN2*-3WJ-4×Bro, or *TCTP1*-3WJ-4×Bro under the *UBQ* promoter were generated and used as scions to graft onto wild-type (WT) rootstocks. At 14 days after grafting, recipient roots were imaged under identical confocal settings in the maturation zone, elongation zone, meristematic zone, and root cap. In WT recipient roots grafted with *TCTP1*-3WJ-4×Bro scions, fluorescence was detected predominantly in the phloem region and was most strongly enriched in the root tip. By contrast, no detectable fluorescence was observed in the corresponding tissues of 3WJ-4×Bro/WT or *ACTIN2*-3WJ-4×Bro/WT grafts (Fig. 1D). RT–PCR and sequencing of RNA from recipient roots further confirmed the presence of *TCTP1*-3WJ-4×Bro transcripts in WT rootstocks (Fig. EV1B,C).

To independently validate the localization reported by 3WJ-4×Bro, we performed fluorescence in situ hybridization (FISH) using a Cy3-labeled probe against *TCTP1* mRNA in stems and roots of *TCTP1*-3WJ-4×Bro-expressing *N. benthamiana*. The 3WJ-4×Bro signal showed strong co-localization with the Cy3-FISH signal in both tissues, with Pearson’s correlation coefficients of 0.80 in stems and 0.87 in roots (Fig. 1E). No specific Cy3 signal was detected in mock-infiltrated tissues under the same imaging conditions (Fig. EV1D). Together, these results establish 3WJ-4×Bro as a reliable tool for visualizing long-distance movement of mobile mRNAs in intact plants.

### *GAI* is a highly mobile transcript in Arabidopsis

**Figure 2:**
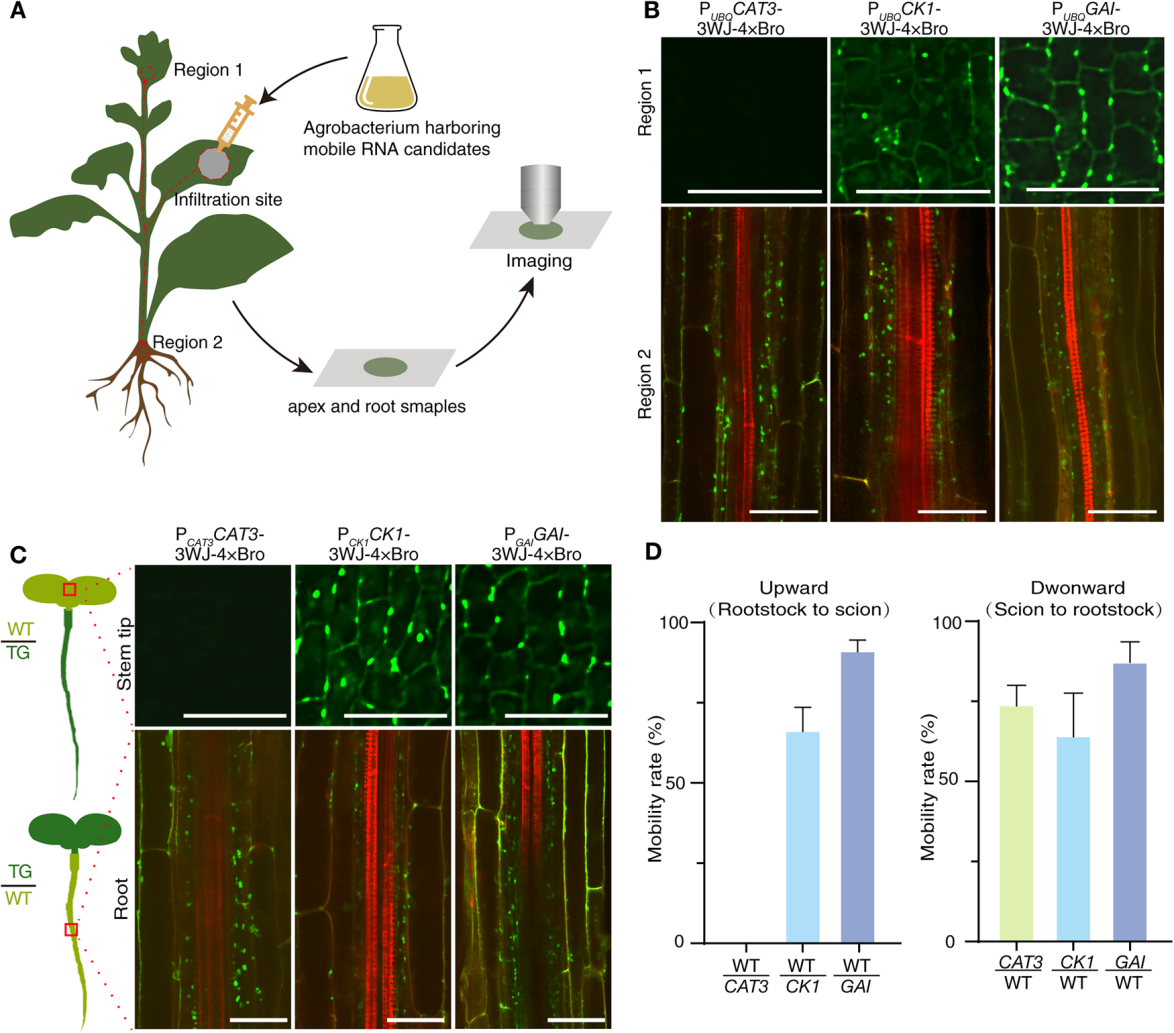
Identification of highly mobile mRNAs in *Arabidopsis* using 3WJ-4×Bro. (**A**) Workflow for screening RNA-seq-predicted mobile mRNAs. 3WJ-4×Bro-tagged candidate transcripts were transiently expressed in *N. benthamiana* leaves, and fluorescence was monitored in region 1 (stem tip 100 mm above the infiltration site) and region 2 (upper root phloem 100 mm below the infiltration site). (**B**) Representative confocal images showing distal fluorescence in roots and stem tips of *N. benthamiana* expressing *CK1*-, *CAT3*-, or *GAI*-3WJ-4×Bro fusions under the *pUBQ* promoter. Cell boundaries are indicated by PI staining (red). Scale bars, 50  μm (root), 50 μm (stem tip). (**C**) Confocal images of 3WJ-4×Bro signals in WT rootstocks and WT stem tips from reciprocal grafts between WT and transgenic *A. thaliana* lines expressing *CAT3*-, *CK1*-, or *GAI*-3WJ-4×Bro under their native promoters. Root samples were collected 1.5 cm below the graft junction at 14 days after grafting (> 2 independent transgenic lines per construct, each *n* > 10 plants). Cell boundaries are indicated by PI staining (red). Scale bars, 25 μm (root), 25 μm (stem tip). (**D**) Schematic summary of fluorescence detection frequencies in WT scions (WT/transgenic) and WT rootstocks (transgenic/WT) for each construct. In total, 15 independent grafts were examined for each construct under identical confocal imaging settings. A plant was scored as fluorescence-positive when a clear 3WJ-4×Bro signal was detected in the WT scion (left panel) and in the rootstock (right panel). The mobility rates were calculated as the percentage of fluorescence-positive plants among the total number of plants examined. Bars represent the mean ± SD. Five independent transformants were analyzed per construct, and all experiments were repeated three times with similar results. Source data are available online for this figure.

Having established 3WJ-4×Bro as a reliable reporter, we next sought to identify highly mobile mRNAs from a previously reported dataset comprising more than 918 predicted graft-mobile transcripts (Thieme et al, 2015). Because imaging-based validation of all candidates was not practically feasible, we randomly selected 100 transcripts for first-pass screening using the 3WJ-4×Bro system (Table EV1). Each candidate coding sequence was fused to 3WJ-4×Bro and transiently expressed in *N. benthamiana* leaves (Fig. 2A). Distal fluorescence was then monitored in stem tips (region 1) and upper roots (region 2) at 56 hpi. Among the 100 candidates tested, only three fusion transcripts—*GAI* (AT1G14920), *CK1* (AT1G71697), and *CAT3* (AT1G20620)—produced clear fluorescence in both distal regions, indicating reproducible long-distance movement under our assay conditions (Fig. 2B).

**Figure EV2:**
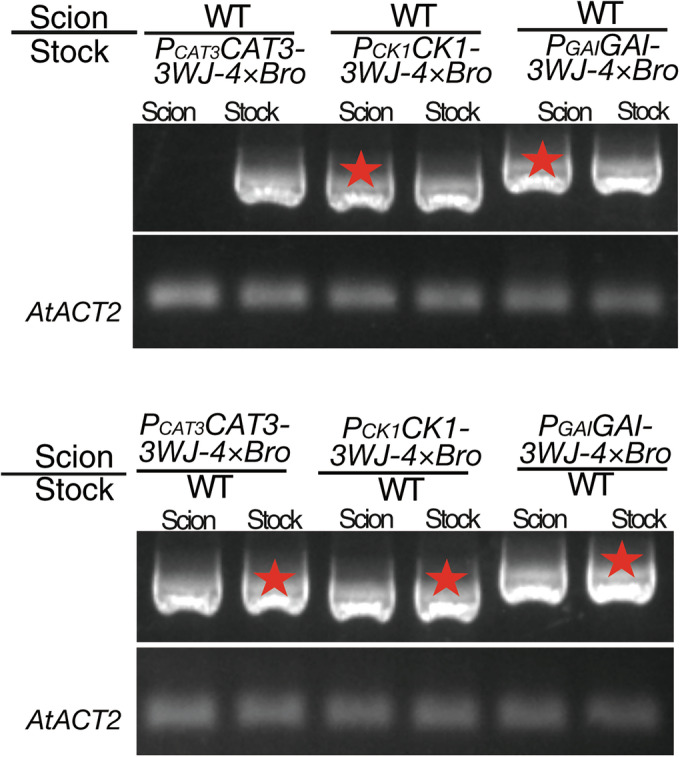
The RT–PCR analyses of 3WJ-4×Bro-tagged transcripts expressed under native promoters in *A. thaliana.* The RNAs were extracted from *A. thaliana* (Col-0) stocks (grafted with transgenic scions), and in the stem tip of wild-type scions (grafted onto transgenic stocks). The root samples were collected 1.5 cm away from the grafting junction at 14 days post-grafting. The gene *A. thaliana ACTIN2* (*AtACT2*) was used as a control. The red five-pointed stars are placed on the gel electrophoresis image to indicate where the fusion transcripts were successfully detected in wild-type plants.

To exclude the possibility that these signals reflected artifacts caused by high transcript abundance in the transient system, we generated transgenic *A. thaliana* lines expressing *CAT3*-3WJ-4×Bro, *CK1*-3WJ-4×Bro, or *GAI*-3WJ-4×Bro under their native promoters. Reciprocal grafting between these lines and WT plants showed that *CAT3*-3WJ-4×Bro fluorescence was detected only in WT rootstocks, consistent with rootward movement, whereas *CK1*-3WJ-4×Bro and *GAI*-3WJ-4×Bro signals were detected in both WT scions and WT rootstocks, indicating bidirectional movement across the graft junction (Fig. 2C). These movement patterns were further supported by RT–PCR analysis of the graft partners (Fig. EV2).

We then quantified the frequency of distal fluorescence detection in WT scions and WT rootstocks. Across five independent transgenic lines and multiple successful grafts, *GAI*-3WJ-4×Bro displayed higher detection frequencies in both directions than *CK1*-3WJ-4×Bro or *CAT3*-3WJ-4×Bro (Fig. 2D). Consistent with previous reports of *GAI* mRNA mobility (Huang and Yu, 2009), these results identify *GAI* as a highly efficient long-distance mobile transcript.

### Two distinct RNA elements confer *GAI* mRNA mobility

**Figure 3:**
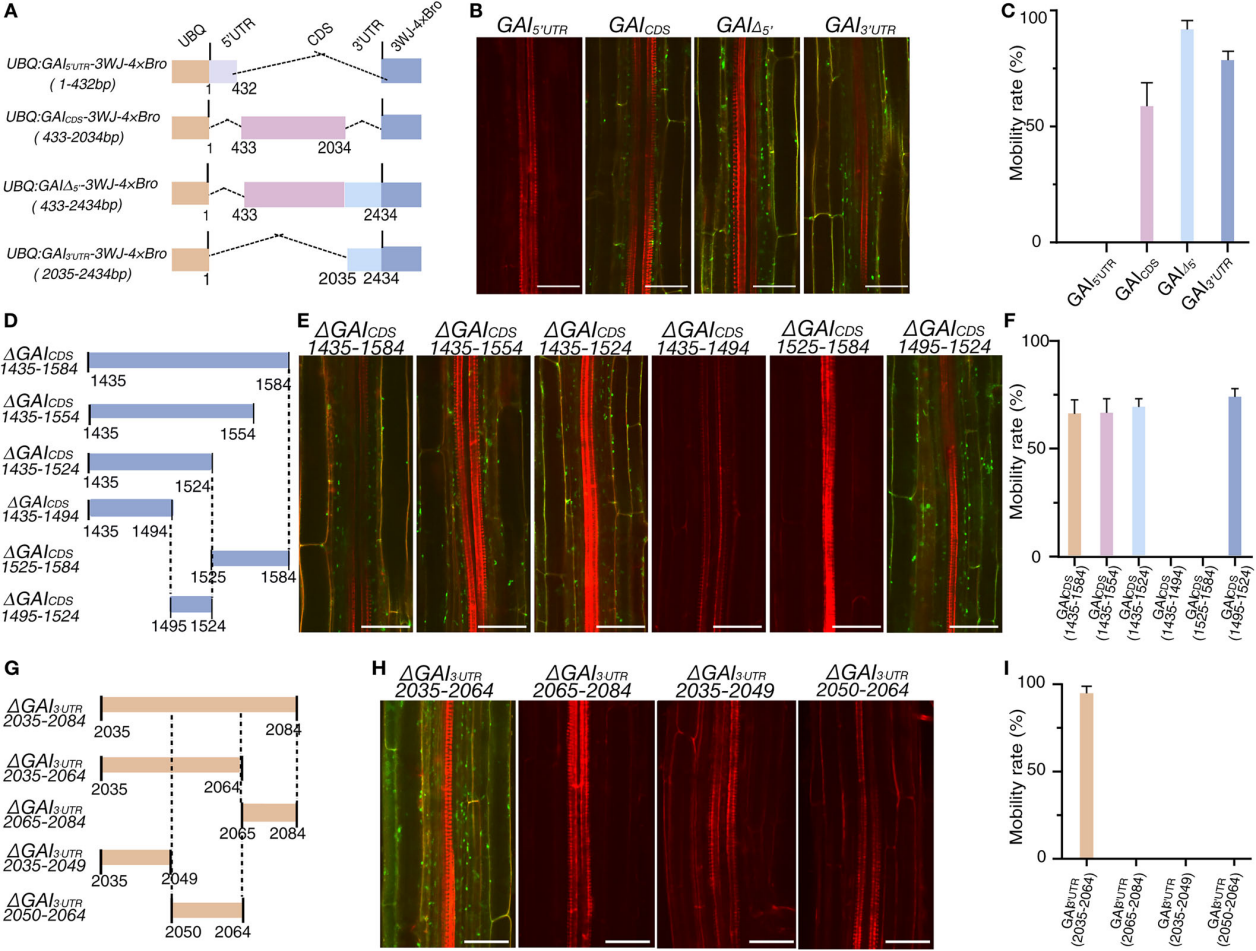
Mapping cis-elements required for *GAI* mRNA mobility. (**A**) Schematic representation of 3WJ-4×Bro-tagged *GAI* deletion constructs used for mobility assays. Orange, *pUBQ* promoter; purple, 5′UTR; blue, CDS; yellow, 3′UTR; green, 3WJ-4×Bro. Dashed lines indicate deleted regions. (**B**) Representative confocal images of 3WJ-4×Bro-tagged *GAI* deletion variants in WT rootstocks 1.5 cm below the graft junction. Cell boundaries are indicated by PI staining (red). Scale bars, 25 μm. (**C**) Quantification of fluorescence detection frequencies in WT rootstocks grafted with *pUBQ*::*GAI* deletion variant scions. (**D**) Schematic representation of the truncation strategy used to fine-map the mobility element within the GAI_CDS_. (**E**) Representative confocal images of 3WJ-4×Bro-tagged GAI_CDS_ truncation variants in WT rootstocks 1.5 cm below the graft junction. Cell boundaries are indicated by PI staining (red). Scale bars, 25 μm. (**F**) Quantification of fluorescence detection frequencies in WT rootstocks grafted with *pUBQ::GAI*_*CDS*_ truncation variant scions. (**G**) Schematic representation of the truncation strategy used to fine-map the mobility element within the GAI_3′UTR_. (**H**) Representative confocal images of 3WJ-4×Bro-tagged GAI_3’UTR_ truncation variants in WT rootstocks 1.5 cm below the graft junction. Cell boundaries are indicated by PI staining (red). Scale bars, 25 μm. (**I**) Quantification of fluorescence detection frequencies in WT rootstocks grafted with *pUBQ::GAI*_*3’UTR*_ truncation variant scions. For (**C**, **F**, **I**), 15 independent grafts were examined for each construct under identical confocal imaging settings. A plant was scored as fluorescence-positive when a clear 3WJ-4×Bro signal was detected in the WT rootstock. The mobility rates were calculated as the percentage of fluorescence-positive plants among the total number of plants examined. Data are shown as mean ± SD. Five independent transformants were analyzed per construct, and all experiments were repeated three times with similar results. Source data are available online for this figure.

**Figure EV3:**
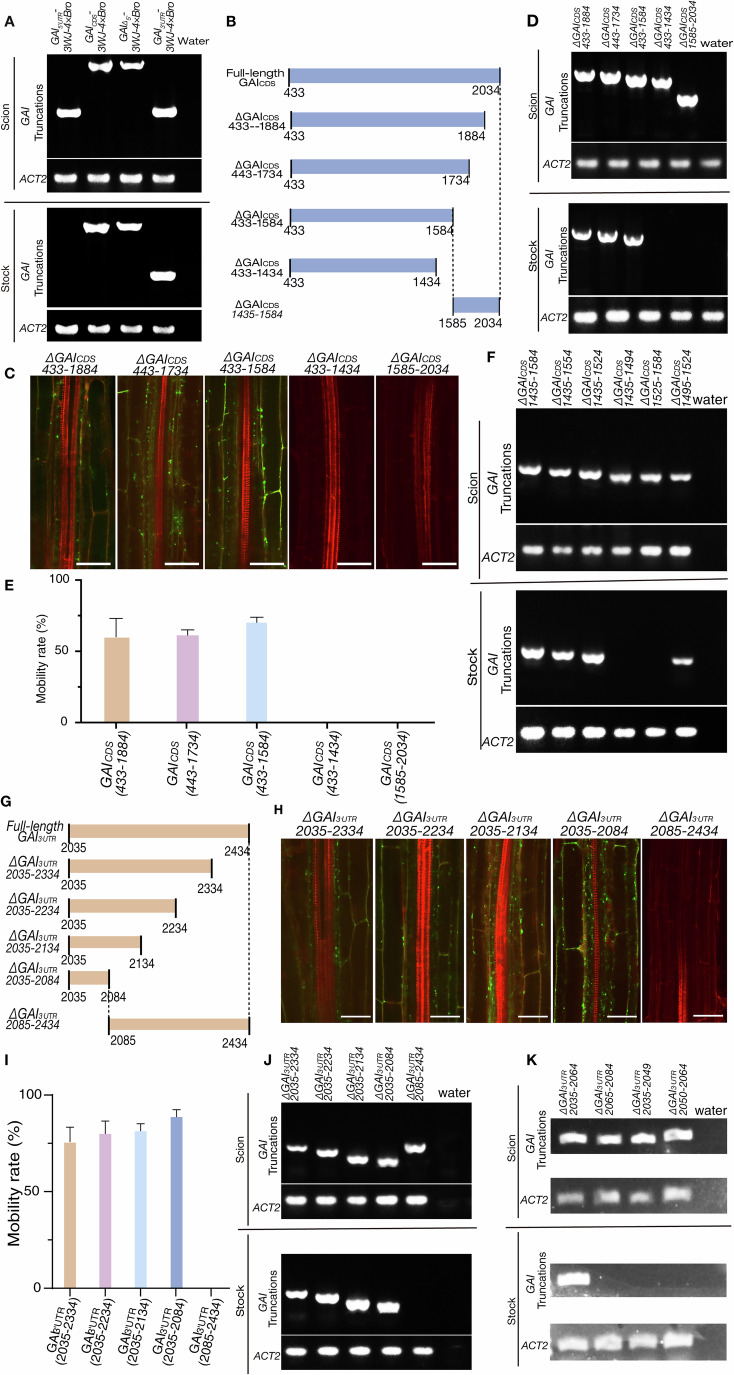
Identification of mobile motifs in *GAI* mRNA using the 3WJ-4×Bro tag. (**A**) RT–PCR detection of *GAI* deletion transcripts in WT *A. thaliana* rootstocks grafted with *pUBQ::GAI* deletion variant scions. Samples were collected 1.5 cm below the graft junction at 14 days post-grafting. *AtACT2* was used as the reference gene. (**B**) Schematic representation of the GAI_CDS_ truncation strategy. (**C**) Representative confocal images of GAI_CDS_ truncation variants in WT *A. thaliana* rootstocks grafted with *pUBQ::GAI* mutant scions. Cell boundaries are indicated by PI staining (red). Scale bars, 25  μm. (**D**) RT–PCR detection of GAI_CDS_ truncation transcripts in WT rootstocks. *AtACT2* was used as the reference gene. (**E**) Quantification of fluorescence detection frequencies in WT rootstocks grafted with *pUBQ::GAI*_*CDS*_ truncation variant scions. (**F**) RT–PCR detection of GAI_CDS_ fine-mapping constructs in WT rootstocks. *AtACT2* was used as the reference gene. (**G**) Schematic representation of the GAI_3′UTR_ truncation strategy. (**H**) Representative confocal images of GAI_3′UTR_ truncation variants in WT *A. thaliana* rootstocks grafted with *pUBQ::GAI* mutant scions. Cell boundaries are indicated by PI staining (red). Scale bars, 25  μm. (**I**) Quantification of fluorescence detection frequencies in WT rootstocks grafted with *pUBQ::GAI*_*3’UTR*_ truncation variant scions. (**J**) RT–PCR detection of GAI_3′UTR_ truncation transcripts in WT rootstocks. *AtACT2* was used as the reference gene. (**K**) RT–PCR detection of GAI_3′UTR_ fine-mapping constructs in WT rootstocks. *AtACT2* was used as the reference gene. For (**E**, **I**), 15 independent grafts were examined for each construct under identical confocal imaging settings. A plant was scored as fluorescence-positive when a clear 3WJ-4×Bro signal was detected in the WT rootstock. Mobility rates were calculated as the percentage of fluorescence-positive plants among the total number of plants examined. Data are shown as mean ± SD from five independent transformants. Each experiment was repeated three times with similar results.

Although *GAI* mRNA has previously been shown to move over long distances, the sequences responsible for this mobility have remained unresolved (Huang and Yu, 2009). To localize the mobility-conferring regions, we carried out a series of deletion analyses using 3WJ-4×Bro-tagged *GAI* variants expressed in *Arabidopsis*. Full-length *GAI* mRNA, its coding sequence (GAI_CDS_), 5’UTR, and 3’UTR were separately fused to 3WJ-4×Bro under the *UBQ* promoter (Fig. 3A). These transgenic lines were used as scions grafted onto WT rootstocks, and fluorescence in recipient WT roots was examined 1.5 cm below the graft junction at 14 days after grafting (Fig. 3B). Fluorescence was detected in WT rootstocks grafted with scions expressing GAI_CDS_-3WJ-4×Bro, GAI_△5’_-3WJ-4×Bro or GAI_3’UTR_ -3WJ-4×Bro, with detection rates of 71.79%, 97.56%, and 85.71%, respectively (Fig. 3C). In contrast, no signal was observed in WT rootstocks grafted with GAI_5’UTR_-3WJ-4×Bro scions (Fig. 3B,C). RT–PCR analysis of WT rootstocks yielded consistent results (Fig. EV3A). These data indicate that both the *GAI* coding region and 3’UTR contain *cis*-elements sufficient to drive long-distance transport, whereas the 5’UTR is dispensable for *GAI* mRNA trafficking.

To fine-map the mobility element within the coding region, we generated a series of 150-nt truncations spanning GAI_CDS_ (Fig. EV3B). Deletion of nucleotides 1435–1584 abolished long-distance movement, whereas the isolated fragment spanning nucleotides 1585–2034 was insufficient to confer mobility when fused to 3WJ-4×Bro (Fig. EV3C–E). Further refinement identified a 30-nt fragment encompassing nucleotides 1495–1524 that was both necessary and sufficient for long-distance movement in the grafting assay (Figs. 3D–F and  EV3F). We designated this element GME1.

A similar truncation strategy was then applied to the GAI_3’UTR_. This analysis identified a 30-nt region corresponding to nucleotides 2035–2064 as necessary for long-distance movement of the 3′UTR fragment in grafted plants (Fig. EV3G–J). Splitting this 30-nt segment into two 15-nt fragments abolished long-distance movement (Figs. 3G–I and  EV3K), indicating that the intact 30-nt sequence is required. We designated this element GME2. Together, these results define two distinct 30-nt cis-elements, GME1 within the coding region and GME2 within the 3′UTR, that independently confer long-distance mobility on *GAI*-derived transcripts.

### GME2 outperforms GME1 in long-distance transport

**Figure 4:**
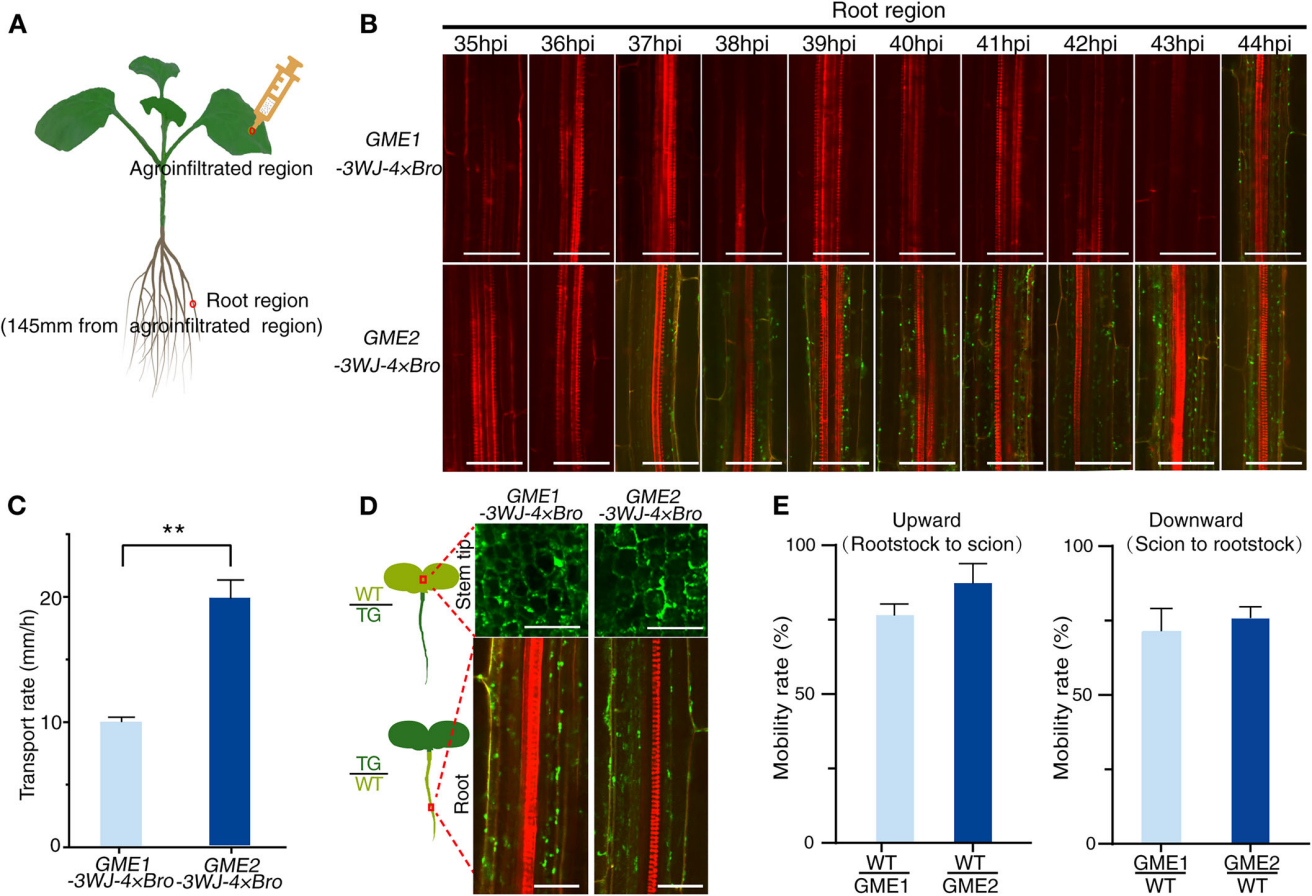
Comparative transport efficiency of GME1 and GME2. (**A**) Schematic of long-distance trafficking assays in *N. benthamiana*. Region 1 corresponds to the agroinfiltrated leaf, and the root region is 145 mm below the infiltration region. (**B**) Representative longitudinal optical section images of the root region showing the distribution of 3WJ-4×Bro fluorescence in vascular tissue identified based on its anatomical position within the root stele. Cell boundaries are indicated by PI staining (red). Scale bars, 50 μm. (**C**) Transport rates of GME1- and GME2-tagged RNAs into the root region. Bars represent mean ± SD. Statistical significance was assessed using unpaired two-tailed Student’s *t* test. **P = 0.0004. The transport rate was defined as the distance from the inoculation site to the designated distal region divided by the time after inoculation at which the first fluorescence-positive plant was detected in that region. (**D**) Representative confocal images of 3WJ-4×Bro signals in WT stem tips and rootstocks from reciprocal grafts between WT and transgenic *A. thaliana* lines expressing GME1-3WJ-4×Bro or GME2-3WJ-4×Bro. Cell boundaries are indicated by PI staining (red). Scale bars, 25 μm (stem tip), 20 μm (root). (**E**) Mobility rate comparison between GME2 and GME1 in *A. thaliana* grafts. In total, 15 independent grafts were examined for each construct under identical confocal imaging settings. A plant was scored as fluorescence-positive when a clear 3WJ-4×Bro signal was detected in the WT scion (left panel) and in the rootstock (right panel). The mobility rates were calculated as the percentage of fluorescence-positive plants among the total number of plants examined. Bars represent the mean ± SD. Five independent transformants were analyzed per construct, and all experiments were repeated three times with similar results. Source data are available online for this figure.

**Figure EV4:**
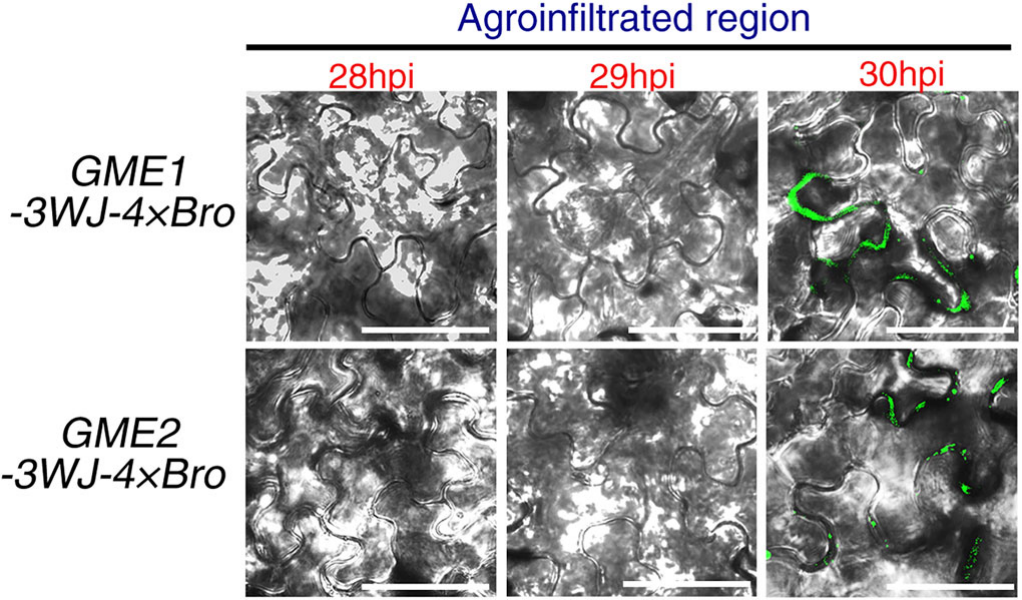
Representative imaging of 3WJ-4×Bro-tagged the two mobile motifs in the agroinfiltrated leaf region of *N. benthamiana* after Agrobacterium inoculation. Fluorescence monitoring was initiated at the *N. benthamiana* inoculation site starting at 28 h post-inoculation (hpi), with hourly observations thereafter. Scale bars represent 50 μm (leaf). The experiments above were repeated three times independently.

We next compared the transport properties of GME1 and GME2 in vivo. Each 30-nt element was fused individually to 3WJ-4×Bro and transiently expressed in *N. benthamiana* leaves (Fig. 4A). Expression of both fusion transcripts in the infiltrated region was confirmed by confocal imaging at 30 hpi (Fig. EV4).

To assess transport kinetics, we performed hourly time-lapse imaging of the root region located 145 mm from the agroinfiltration site. Fluorescence derived from GME2-3WJ-4×Bro was first detected in distal vascular tissue at 37 hpi, whereas GME1-3WJ-4×Bro fluorescence was first detected at 44 hpi (Fig. 4B). Quantification of transport rate revealed that GME2-3WJ-4×Bro moved significantly faster than GME1-3WJ-4×Bro to the same distal region (Fig. 4C).

We then examined directional mobility in *A. thaliana* grafts using transgenic lines expressing GME1-3WJ-4×Bro or GME2-3WJ-4×Bro under the *UBQ* promoter. Reciprocal grafting with WT plants showed fluorescence in both WT stem tips and WT rootstocks for each construct, indicating that both elements support bidirectional movement across the graft junction (Fig. 4D). However, quantitative analysis revealed that the mobility rate of GME2-3WJ-4×Bro in WT partners was consistently higher than that of GME1-3WJ-4×Bro (Fig. 4E). Thus, although both elements are mobile in both directions, GME2 shows superior transport rate and mobility rate, making it the more suitable candidate for engineering an RNA delivery module.

### 2×GME2 functions as an efficient RNA delivery module

**Figure 5:**
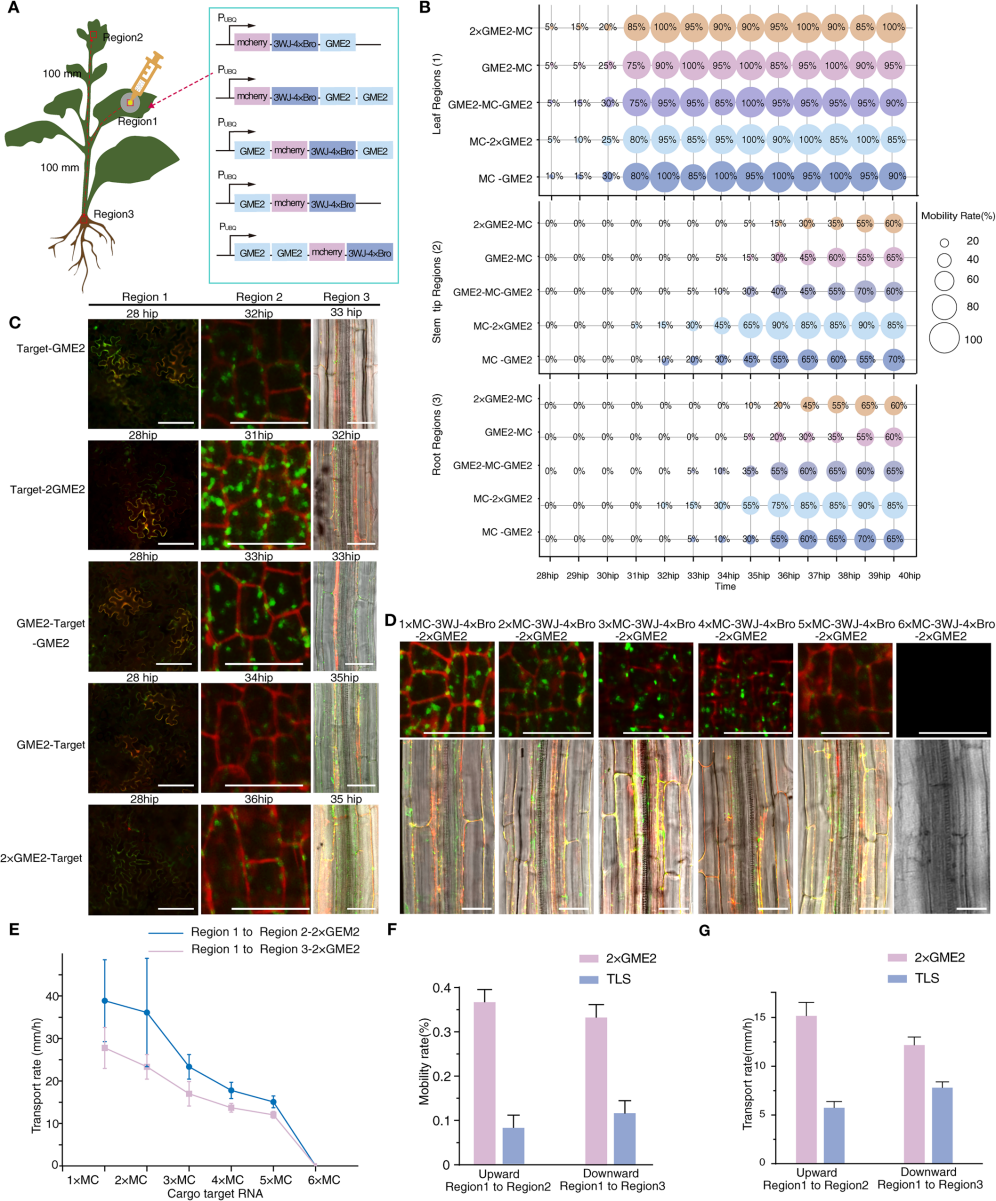
Performance of GME2 as a long-distance RNA delivery module. (**A**) Schematic of long-distance trafficking assays for mCherry transcripts fused to GME2-based delivery cassettes in *N. benthamiana*. Region 1 denotes the agroinfiltrated leaf, whereas region 2 and region 3 correspond to the stem tip and upper root phloem, respectively, each located 100 mm from region 1. Pink, mCherry coding sequence; blue-violet, 3WJ-4×Bro aptamer; light blue, GAI_3’UTR_2035-2064 (GME2). (**B**) Time-course analysis of mobility rates of GME2-derived constructs in agroinfiltrated *N. benthamiana*. Numbers within the circles indicate the percentage of plants with detectable distal fluorescence. Twenty independent inoculated plants were examined for each construct at each time point under identical confocal imaging settings in the predefined distal regions. A plant was scored as fluorescence-positive when a clear 3WJ-4×Bro signal was detected in the designated distal region. The mobility rates were calculated as the percentage of fluorescence-positive plants among the total number of plants examined. (**C**) Representative confocal images showing the first detectable fluorescence in regions 1–3 for each construct. The time post-inoculation at which the signals were first observed is indicated above each panel. Fluorescence signals of mCherry (red) and 3WJ-4×Bro (green) are shown. Scale bars, 100 μm (leaf), 25 μm (stem tip), 25 μm (root). (**D**) Confocal images showing distal fluorescence signals of mCherry (red) and 3WJ-4×Bro (green) in stem tips and roots at 56 hpi for constructs carrying increasing copy numbers of mCherry fused to the 2×GME2 cassette. Scale bars, 25 μm (stem tip), 25 μm (root). (**E**) Transport rates of size-various cargo (1× to 6× mCherry) mediated by 2×GME2 in the upward direction (region 1 to region 2) and the downward direction (region 1 to region 3). The transport rate was defined as the distance from the inoculation site to the designated distal region divided by the time after inoculation at which the first fluorescence-positive plant was detected in that region. Points and error bars represent mean ± SD. (**F**) Mobility rate of 5×mCherry cargo mediated by 2×GME2 or TLS in the upward direction (region 1 to region 2) and downward direction (region 1 to region 3). Bars represent mean ± SD. The mobility rate was calculated as in (**B**). (**G**) Transport rate of 5×mCherry cargo mediated by 2×GME2 or TLS in the upward direction (region 1 to region 2) and downward direction (region 1 to region 3). The transport rate was calculated as in (**E**). Bars represent mean ± SD. Source data are available online for this figure.

To optimize a GME2-based delivery system, we compared a set of GME2-derived constructs using mCherry-3WJ-4×Bro as a reporter, varying both the position and copy number of the motif. GME2 was inserted into the 5′UTR, the 3′UTR, or both ends of the reporter transcript, and additional constructs carrying tandem GME2 copies were also examined (Fig. 5A). All GME2-containing constructs generated detectable fluorescence in distal tissues, indicating that GME2 is sufficient to promote long-distance movement of the reporter RNA (Fig. 5B). However, the different configurations varied markedly in performance.

**Figure EV5:**
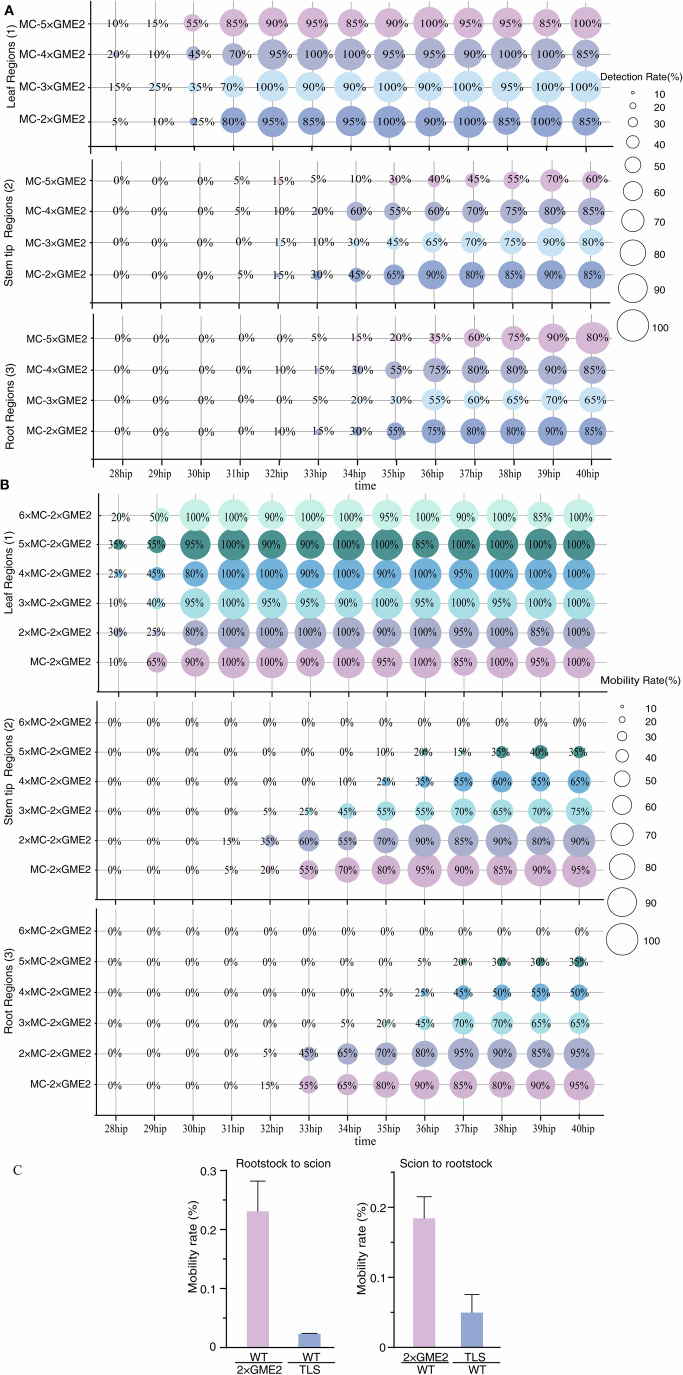
Performance of GME2 as a long-distance RNA delivery module. (**A**, **B**) Statistical analysis of fluorescence-detected rates at three *N. benthamiana* sampling sites (regions 1, 2, and 3) following *Agrobacterium* inoculation with different copy numbers of GME2/mCherry constructs. *X* axis: Time post-inoculation; *Y* axis: Agrobacterium inoculation with six constructs in leaves. *n* = 20 plants per construct per time point. Mobility rates were calculated as described in “Methods”. (**C**) 15 independent grafts were examined for each construct under identical confocal imaging settings. A plant was scored as fluorescence-positive when a clear 3WJ-4×Bro signal was detected in the WT scion (left panel) and in the rootstock (right panel). The mobility rates were calculated as the percentage of fluorescence-positive plants among the total number of plants examined. Bars represent the mean ± SD. Five independent transformants were analyzed per construct, and all experiments were repeated three times with similar results.

Among the positional designs tested, constructs carrying GME2 in the 3′UTR showed the strongest overall transport performance. In particular, the mCherry-3WJ-4×Bro-2×GME2 construct produced the earliest detectable distal fluorescence, with signals first observed at 31 hpi in stem tips (region 2) and 32 hpi in roots (region 3). By 36 hpi in region 2 and 37 hpi in region 3, more than 85% of inoculated plants showed detectable fluorescence (Fig. 5C). Increasing the copy number from one to two improved delivery efficiency, whereas additional copies (3×–5×) provided no obvious further benefit (Fig. EV5A). We therefore selected the 3′UTR-2×GME2 configuration for subsequent experiments.

We next evaluated the cargo capacity of the 2×GME2 system by increasing transcript size through tandem duplication of the mCherry coding sequence. Constructs encoding one to six copies of mCherry, each fused to 2×GME2 in the 3′UTR, were transiently expressed in *N. benthamiana* leaves (Fig. 5D). Time-course imaging showed that 2×GME2 supported transport of transcripts up to approximately 3540–4248 nt to distal stem and root tissues (Fig. 5D). As RNA length increased, however, transport rate progressively declined, reaching maximal rates of approximately 39 mm h⁻¹ from region 1 to region 2 and 28 mm h⁻¹ from region 1 to region 3 for the largest constructs (Fig. 5E). Consistent with this trend, the mobility rate, defined as the percentage of plants showing detectable distal fluorescence, decreased with increasing cargo size. For the 5×mCherry cargo, the mobility rate was approximately 35% (Fig. EV5B). These data indicate that 2×GME2 supports multi-kilobase cargo transport, although transport efficiency decreases as transcript length increases.

Finally, we compared 2×GME2 with a representative TLS-based delivery module using the same 5×mCherry cargo. In both the upward and downward directions, 2×GME2 showed a higher mobility rate than TLS (Fig. 5F). Likewise, 2×GME2 mediated faster transport than TLS in both directions (Figs. 5G and  EV5C). Thus, under our assay conditions, 2×GME2 supports both faster and more efficient long-distance transport of the same RNA cargo than the representative TLS module tested here.

### 2×GME2 delivers functional mRNAs to improve stress tolerance in grafted plants

**Figure 6:**
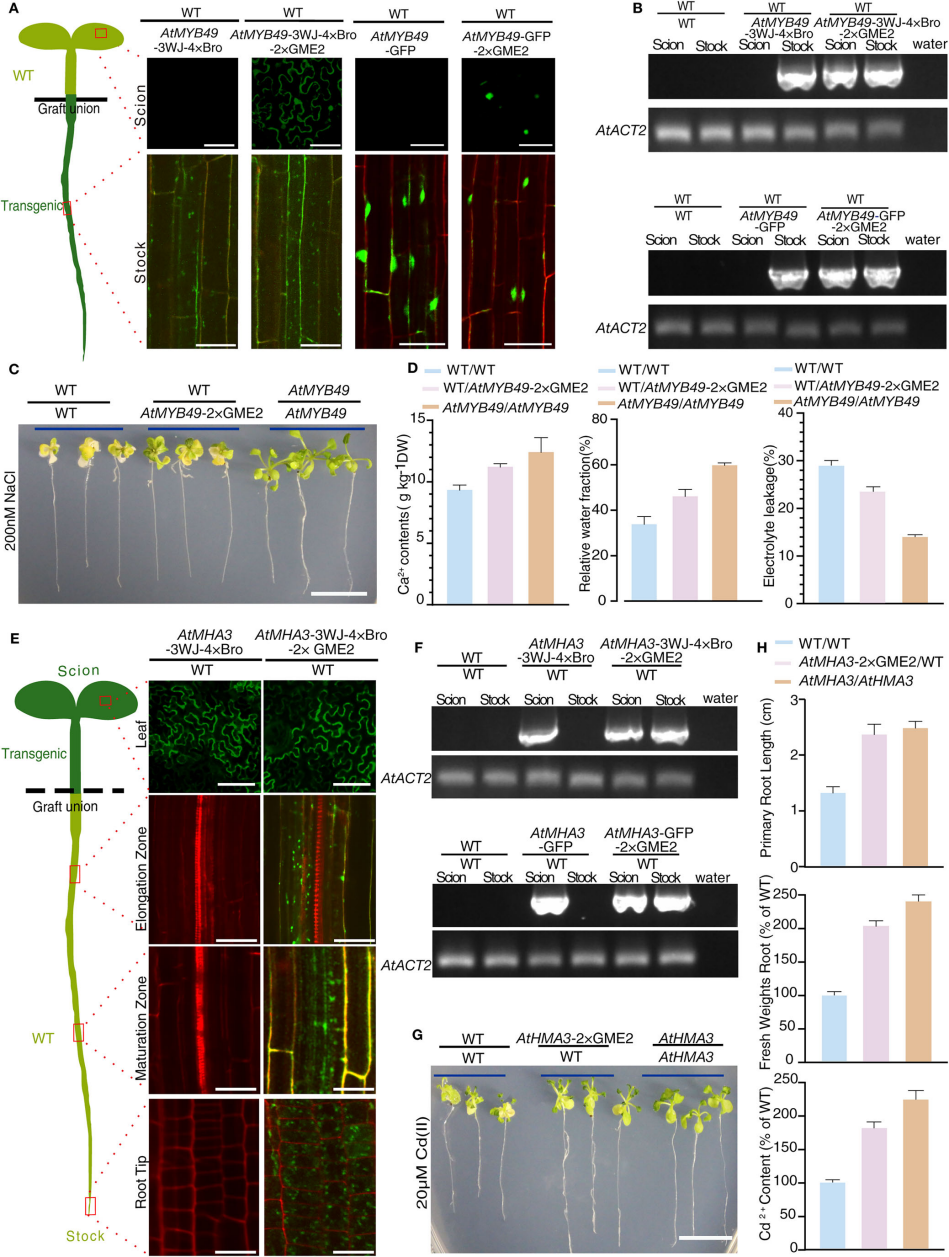
2×GME2-mediated delivery of functional mRNAs enhances stress tolerance in grafted *A. thaliana.* (**A**) Representative confocal images of scion and rootstock showing distribution of 3WJ-4×Bro or GFP fluorescence in *AtMYB49*-related grafted plants. Root samples were collected 1.5 cm below the graft junction, and leaf samples ≥1 cm above the junction at 14 days after grafting (> 2 independent transgenic lines per construct, each *n* > 10 plants). Scale bars, 100 μm (leaves), 25 μm (roots). (**B**) RT–PCR detection of 3WJ-4×Bro- or GFP- tagged *AtMYB49* RNA in rootstocks and scions. *AtACT2* was used as the reference gene for RNA quality control, and water was used as the negative template control. (**C**) Phenotypes of graft combinations WT/WT, WT/*AtMYB49*-2×GME2, and *AtMYB49*/*AtMYB49* grown on 1/2 MS medium supplemented with 200 mM NaCl for 14 days. Scale bars, 15 mm. (**D**) Quantification of leaf Ca²⁺ content (g kg^−^¹ DW), relative water fraction (RWC, %), and electrolyte leakage (%) in the three graft combinations after 10 days of NaCl treatment (*n* = 3 biological replicates; 12 plants per genotype per replicate). (**E**) Representative confocal images of different root regions showing the distribution of the mobile *HMA3-*3WJ-4×Bro RNA in the roots of grafted plants. (**F**) RT–PCR detection of 3WJ-4×Bro- or GFP-tagged *HMA3* RNA in rootstocks and scions. *AtACT2* was used as the reference gene for RNA quality control, and water was used as the negative template control. (**G**) Phenotypes of graft combinations WT/WT, *AtHMA3*-2×GME2/WT, *AtHMA3*/*AtHMA3* grown on 1/2 MS medium containing 20 μM Cd for two weeks. Scale bars, 15 mm. (**H**) Quantification of primary root length (cm), root fresh weight (g), and root Cd²⁺ content for the indicated graft combinations after two weeks of treatment (*n* = 3 biological replicates; 12 plants per genotype per replicate). All experiments were repeated three times with similar results. Source data are available online for this figure.

We next asked whether 2×GME2 could deliver functional, otherwise non-mobile mRNAs across graft unions and thereby alter recipient phenotypes. To test upward delivery from transgenic rootstock to WT scion, we selected *AtMYB49*, a transcription factor whose overexpression in leaves enhances salt tolerance (Zhang et al, 2020). For transcript and protein visualization, 3WJ-4×Bro or *GFP* was fused to *AtMYB49*, respectively (Fig. 6A). In rootstocks, strong 3WJ-4×Bro fluorescence and nucleus-localized GFP fluorescence were observed irrespective of the presence of 2×GME2. In WT scions, however, both 3WJ-4×Bro and GFP signals were detected only when the rootstock carried the *AtMYB49*-2×GME2 construct, but not when 2×GME2 was absent (Fig. 6A). RT–PCR analysis further confirmed the presence of the corresponding tagged *AtMYB49* transcripts in recipient scions (Fig. 6B). These results support upward movement of *AtMYB49* mRNA and its translation in recipient tissues when coupled to 2×GME2.

**Figure EV6:**
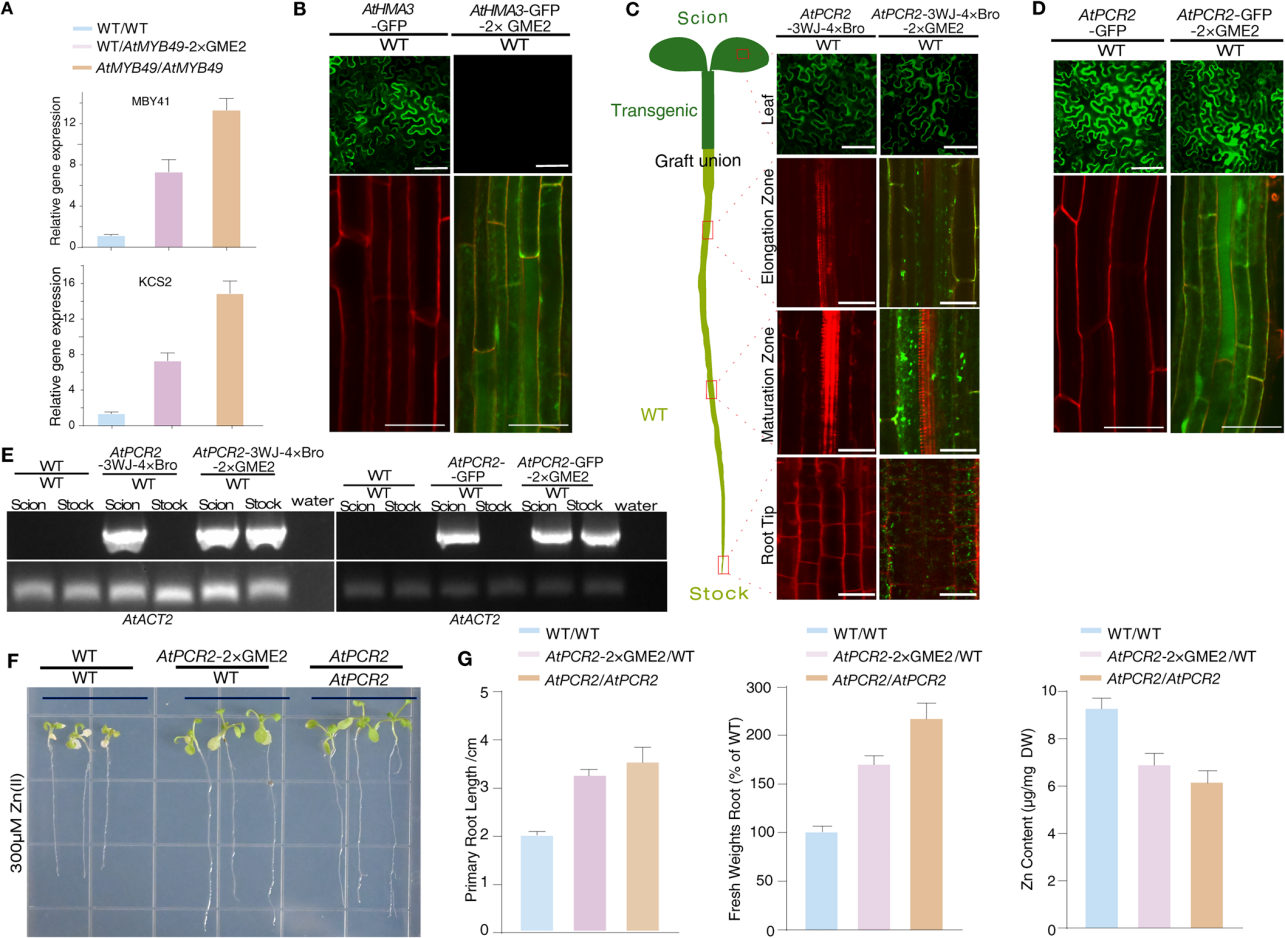
2×GME2-mediated delivery of functional mRNAs enhances stress tolerance in grafted *A. thaliana.* (**A**) Quantitative RT–PCR detection of the expression levels of two *MYB49*-regulated genes *MYB41* and *KCS2* in scions. *AtACT2* was used as the reference gene. (**B**) Representative confocal images of scion and rootstock showing the distribution of *HMA3*-GFP protein in grafted plants. (**C**) Representative confocal images of different root regions showing the distribution of the mobile *PCR2*-3WJ-4×Bro RNA in the roots of grafted plants. (**D**) Representative confocal images of scion and rootstock showing the distribution of *PCR2*-GFP protein grafted in plants. (**E**) RT–PCR detection of 3WJ-4×Bro- or GFP- tagged *PCR2* RNA in rootstocks and scions. *AtACT2* was used as the reference gene for RNA quality control, and water was used as the negative template control. (**F**) Phenotypes of graft combinations WT/WT, *AtPCR2*-2×GME2/WT, and *AtPCR2*/*AtPCR2* grown on 1/2 MS medium containing 300 μM Zn for two weeks. Scale bars, 15 mm. (**G**) Quantification of primary root length (cm), root fresh weight (g), and root Zn²⁺ content for the indicated graft combinations after two weeks of treatment (*n* = 3 biological replicates; 12 plants per genotype per replicate). All experiments were repeated three times with similar results.

We then examined whether the transfer of *AtMYB49* mRNA affected salt tolerance in grafted plants. Compared with WT/WT grafts, WT/*AtMYB49*-2×GME2 grafts displayed markedly improved performance under NaCl treatment and resembled *AtMYB49*/*AtMYB49* grafts (Fig. 6C). This improvement was accompanied by increased leaf Ca²⁺ content, higher relative water content, and lower electrolyte leakage (Fig. 6D). In addition, quantitative RT–PCR showed that expression of the known *AtMYB49* target genes *AtMYB41* and *AtKCS2* was increased in recipient leaves of WT/*AtMYB49*-2×GME2 grafts relative to WT/WT controls (Fig. EV6A), consistent with functional activity of the mobile transcript in the scion.

To test downward delivery from transgenic scion to WT rootstock, we selected *AtHMA3* (Morel et al, 2009) and *AtPCR2* (Song et al, 2010) as cargos. Fluorescence imaging showed that 3WJ-4×Bro-tagged *AtHMA3* and *AtPCR2* transcripts, as well as their GFP-tagged protein signals, were detected in WT rootstocks only when the donor scion contained 2×GME2 (Figs. 6E and EV6B–D). RT–PCR analysis was consistent with these observations (Figs. 6F and  EV6E).

We next assessed the phenotypic consequences of downward delivery. When *AtHMA3*-2×GME2 scions were grafted onto WT rootstocks, recipient WT roots displayed enhanced tolerance to cadmium stress. Relative to WT/WT controls, *AtHMA3*-2×GME2/WT grafts showed longer primary roots, higher root fresh weight, and increased Cd²⁺ accumulation in roots (Fig. 6G,H). Similarly, under zinc stress, *AtPCR2*-2×GME2/WT grafts exhibited longer primary roots, higher root fresh weight, and reduced Zn²⁺ accumulation relative to WT/WT controls (Fig. EV6F,G), consistent with enhanced Zn tolerance.

Together, these results show that 2×GME2 can confer long-distance mobility on non-mobile mRNAs, support their expression in recipient tissues, and enable graft-mediated modulation of the related traits.

## Discussion

Graft-mediated RNA delivery provides an attractive route for non-transgenic trait manipulation because mobile RNA cargos can be produced in donor tissues while recipient organs remain genetically unmodified (Yang et al, 2023). In this study, we identified a compact *GAI* (Huang and Yu, 2009)-derived mobility element, GME2, and engineered it into a tandem 2×GME2 module that supports efficient long-distance transport of diverse RNA cargos. Combined with quantitative 3WJ-4×Bro imaging, our results establish a framework for identifying and evaluating sequence-defined RNA mobility elements and support 2×GME2 as a useful non-viral module for graft-mediated RNA delivery.

One important advance of this study is methodological. Candidate mobile mRNAs have largely been inferred from graft-transcriptome datasets, but such datasets are prone to contamination, ambiguous read assignment, and overestimation of bona fide long-distance movement (Heeney and Frank, 2023; Fu et al, 2024). Fluorescent protein- and MS2-based reporters have helped address some of these limitations, but they remain translation-dependent and are often constrained by background fluorescence and the difficulty of capturing infrequent long-distance transport events in intact plants (Heeney and Frank, 2023). By contrast, the 3WJ-4×Bro system enables direct visualization of RNA transport in living plants without relying on translation of the tagged transcript. Within this framework, only three of the 100 candidates tested here showed reproducible long-distance movement under our experimental conditions, underscoring the importance of direct experimental validation when assessing predicted mobile RNAs.

Our data further indicate that *GAI* contains at least two separable cis-elements capable of promoting long-distance transport. GME1, located in the coding region, appears to display context-dependent mobility and may act together with surrounding sequence features in the native transcript. By contrast, GME2, located in the 3′UTR, behaves as a compact and transferable mobility element: its activity is retained when duplicated and fused to heterologous cargos, and it functions in our assay without recognizable TLS-like structural features or obvious methylation-related sequence signatures associated with other mobile RNAs (Zhang et al, 2016; Li et al, 2025; Yang et al, 2019). These observations support the view that selective long-distance transport can be specified by short sequence elements in addition to previously described epitranscriptomic marks. Whereas m^5^C modification of *TCTP1* and combined m^5^C/m^6^A marks on pumpkin *CK1* exemplify modification-associated mobility (Li et al, 2025; Yang et al, 2019), our results show that a short and structurally simple motif can also be sufficient to promote systemic movement in an engineered context.

**Figure EV8:**
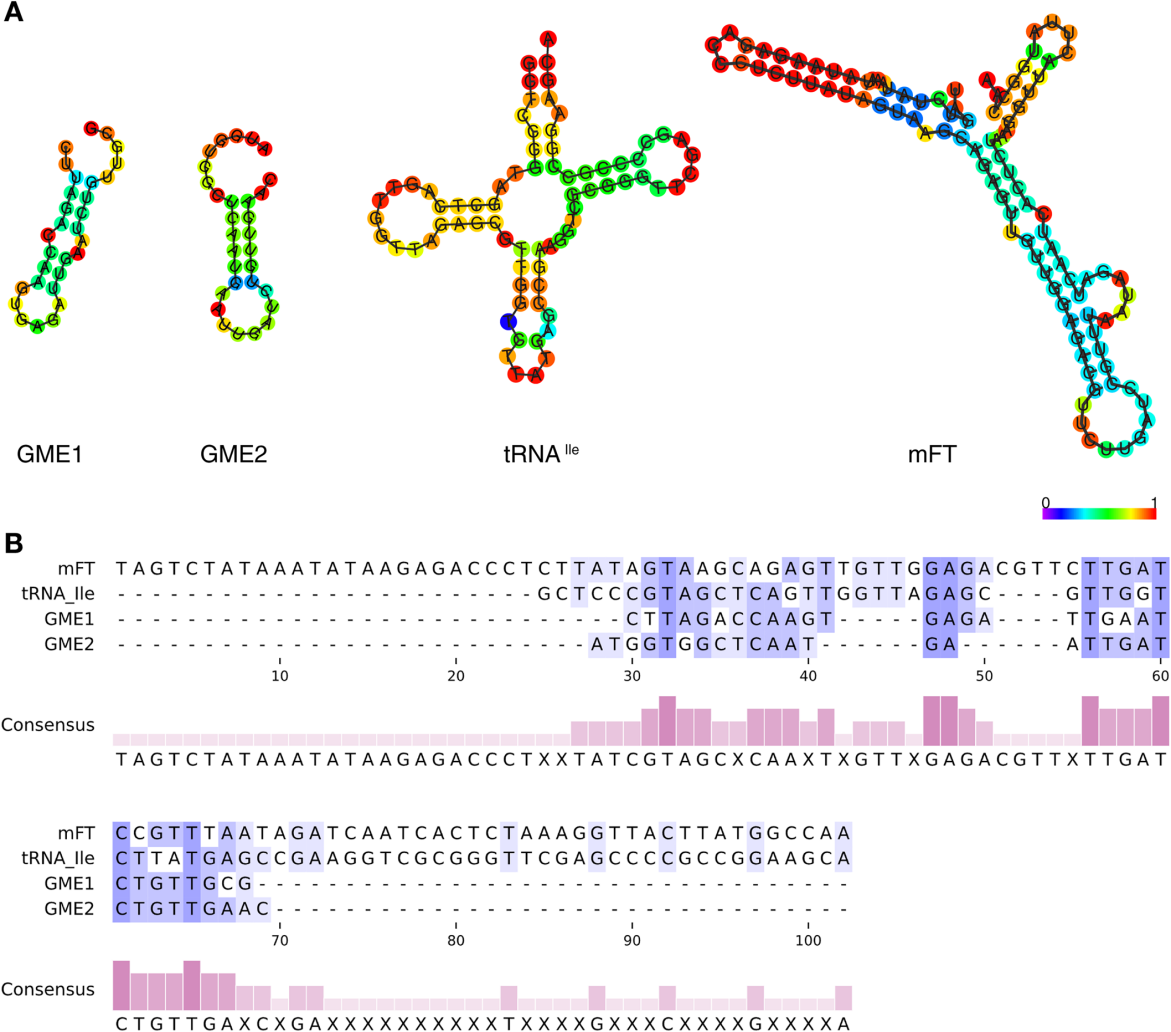
Predicted RNA secondary structures and nucleotide sequence alignment of GME1, GME2, tRNA^Ile^, and mFT. (**A**) RNA secondary structures of GME1, GME2, tRNA^Ile^, and mFT were predicted using the RNAfold web server (http://rna.tbi.univie.ac.at/cgi-bin/RNAWebSuite/RNAfold.cgi). (**B**) Multiple nucleotide sequence alignment of mFT, tRNA^Ile^, GME1, and GME2. Conserved or similar nucleotides are highlighted in blue, and gaps introduced for alignment are indicated by dashes. The consensus sequence and histogram below summarize nucleotide conservation across aligned positions.

Compared with existing RNA delivery strategies, 2×GME2 appears to provide a complementary solution rather than a universal replacement. TLS- and mFT-based mobile RNA modules have been particularly valuable in virus-enabled editing systems, where they can promote the movement of sgRNAs or other cargos toward upper or meristem-associated tissues and, in favorable contexts, support heritable editing (Ellison et al, 2020; Li et al, 2021, Oh et al, 2025). Virus-based systems can also achieve strong systemic spread and high cargo accumulation, but their performance is often influenced by host-virus compatibility, insert-size constraints, vector stability, and the need for further optimization of Cas expression, vector design, environmental conditions, or regeneration procedures (Ma et al, 2020; Ariga et al, 2020; Yoshida et al, 2024). Although 2×GME2 shares partial sequence similarity with TLS and mFT modules (Fig. EV8), 2×GME2 is a new non-viral mobility cassette that can be directly appended to heterologous mRNA cargos and, in the systems tested here, supports rapid long-distance transport, multi-kilobase cargo delivery, translation in recipient tissues, and graft-mediated phenotypic effects. We therefore view 2×GME2 as a modular non-viral RNA delivery element that is particularly well suited for graft-mediated transfer of functional mRNAs, whereas TLS- and virus-based platforms remain especially valuable when systemic infection or meristem-targeted editing is the primary objective.

**Figure EV9:**
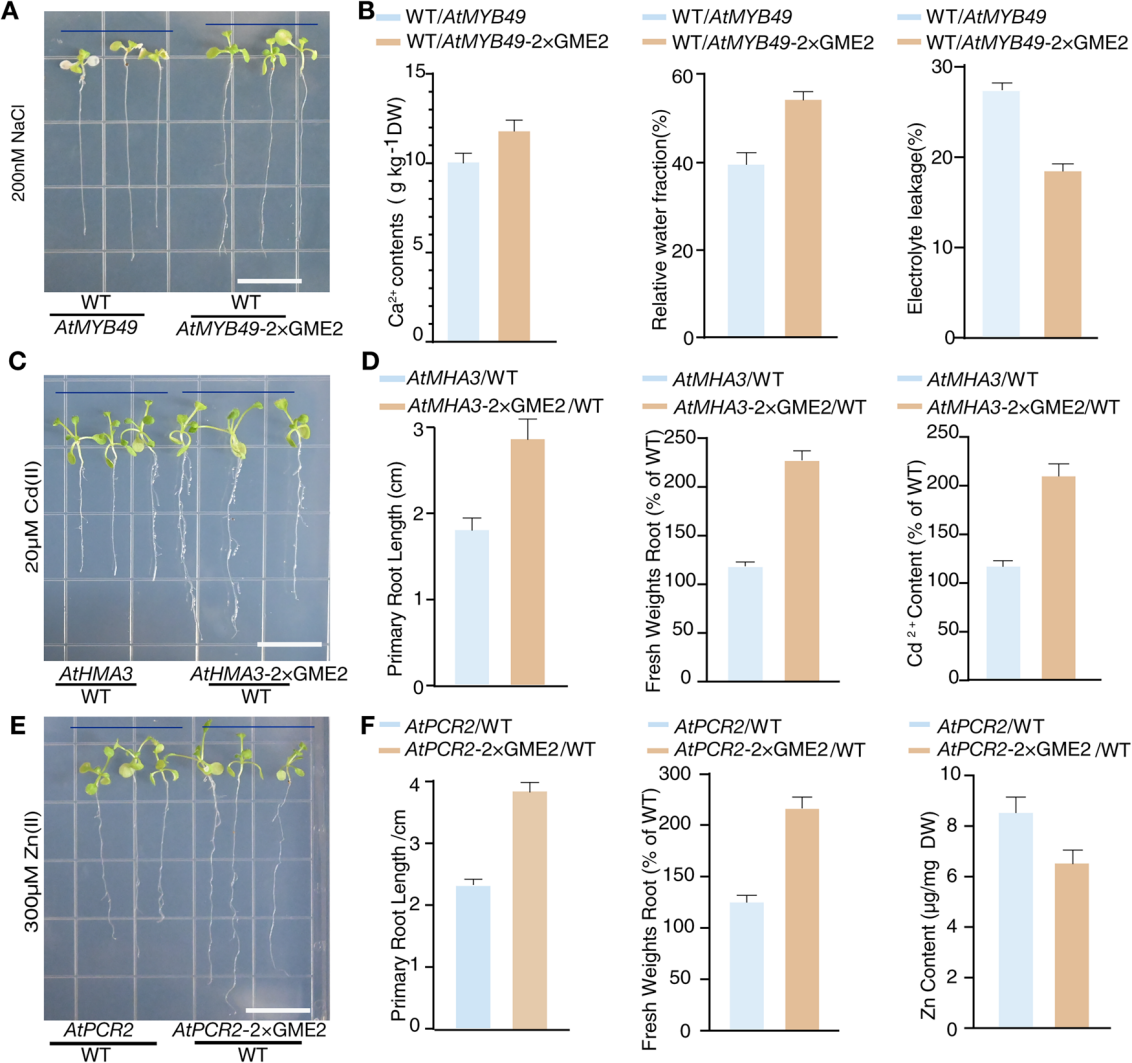
Comparison of 2×GME2-tagged graft combinations with corresponding grafts lacking 2×GME2 under stress conditions. (**A**) Phenotypes of WT/*AtMYB49* and WT/*AtMYB49*-2×GME2 graft combinations grown on 1/2 MS medium supplemented with 200 mM NaCl for 14 days. Scale bar, 10 mm. (**B**) Quantification of leaf Ca²⁺ content, relative water content, and electrolyte leakage in WT/*AtMYB49* and WT/*AtMYB49*-2×GME2 graft combinations after NaCl treatment. (**C**) Phenotypes of *AtHMA3*/WT and *AtHMA3*-2×GME2/WT graft combinations grown on 1/2 MS medium containing 20 μM Cd for two weeks. Scale bar, 10 mm. (**D**) Quantification of primary root length, root fresh weight, and root Cd content in *AtHMA3*/WT and *AtHMA3*-2×GME2/WT graft combinations after Cd treatment. (**E**) Phenotypes of *AtPCR2*/WT and *AtPCR2*-2×GME2/WT graft combinations grown on 1/2 MS medium containing 300 μM Zn for two weeks. Scale bar, 10 mm. (**F**) Quantification of primary root length, root fresh weight, and root Zn content in *AtPCR2*/WT and *AtPCR2*-2×GME2/WT graft combinations after Zn treatment. Data are shown as mean  ± s.d.; *n *= 3 biological replicates, with 12 plants per graft combination per replicate. All experiments were repeated three times with similar results.

From an applied perspective, 2×GME2 broadens the toolkit for graft-mediated manipulation of stress-related traits. Here, fusion of 2×GME2 to *AtMYB49*, *AtHMA3*, or *AtPCR2* enabled delivery of these otherwise non-mobile mRNAs across graft junctions and was associated with detectable functional outputs in recipient tissues. Compared with graft combinations expressing the same mRNA cargo without the mobility cassette, the corresponding 2×GME2-tagged graft combinations showed more pronounced improvements in stress-related phenotypes, with phenotypic improvement trends more closely resembling those observed in the corresponding overexpression lines (Fig. EV9). These results indicate that 2×GME2 is compatible with multiple cargos and can support phenotypic effects in graft partners after long-distance RNA transport. The compact size of the cassette and the absence of viral sequences further make this module an attractive starting point for the development of non-viral RNA delivery strategies in graft-compatible plants.

**Figure EV7:**
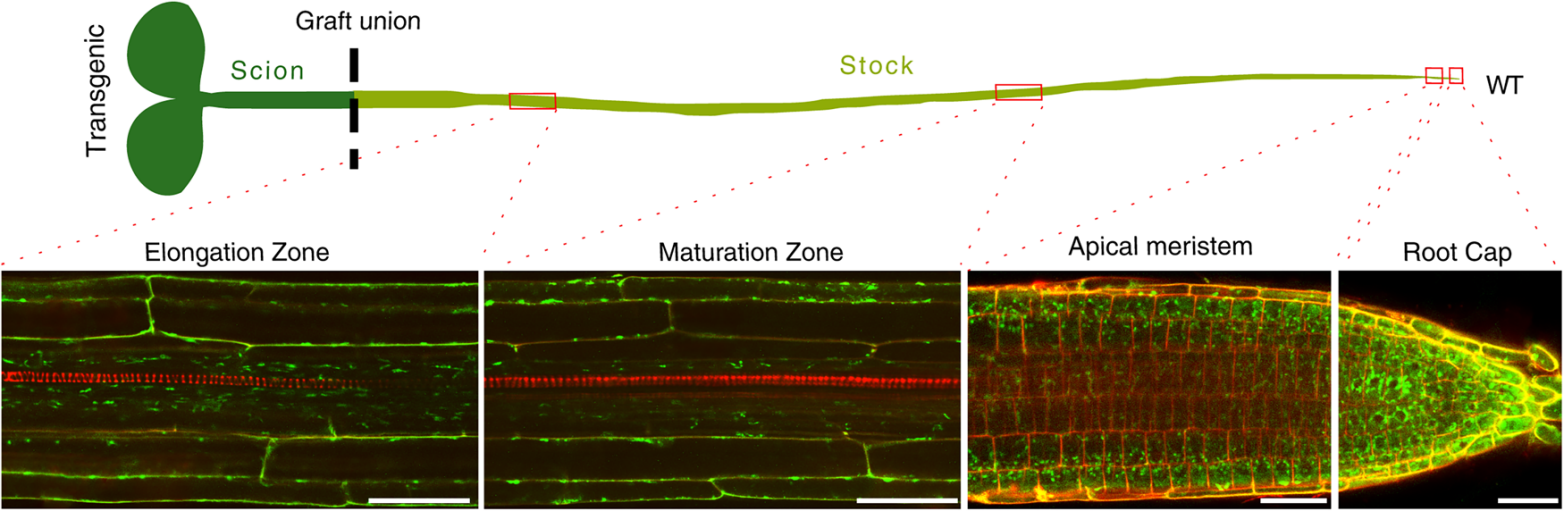
Confocal fluorescence detection of GME2-3WJ-4×Bro RNA in recipient roots after grafting. Representative longitudinal optical section images of roots showing the distribution of 3WJ-4×Bro-GME2 RNA in different root regions at 14 days post-grafting (> 2 independent transgenic lines per construct, each *n* > 10 plants). Cell boundaries are indicated by PI staining (red). Scale bars, 25 μm.

Several questions remain open. Our imaging suggests that GME2-directed cargos are not confined to the transport stream after long-distance movement: in recipient roots, GME2-tagged transcripts were mainly associated with the vascular region in mature root zones, but were also detectable in more peripheral tissues near the root tip, similar to the pattern observed for *TCTP1* (Fig. EV7). Although these data are consistent with unloading into distal sink tissues, the precise radial route of movement remains unresolved. It will be important to identify host factors that interact with GME2 and to determine whether the motif primarily affects phloem loading, stability during transport, unloading, or multiple steps in combination (Yang et al, 2019; Kehr et al, 2022). It will also be necessary to assess how broadly the module performs across different transcript contexts and graft combinations, and whether further engineering of motif copy number, spacing, or orientation can improve delivery efficiency or tissue specificity (Zhang et al, 2016; Yang et al, 2019; Zheng et al, 2020). Even with these limitations, the combination of 3WJ-4×Bro and 2×GME2 provides a useful framework for dissecting RNA mobility and for developing non-viral strategies for graft-mediated RNA delivery.

## Methods

**Table:** Reagents and Tools Table

Reagent/resource	Reference or source	Identifier or catalog number
**Experimental models**		
*Agrobacterium tumefaciens*/GV3101	This study	N/A
*Arabidopsis thaliana*/Col-0	This study	N/A
*Nicotiana benthamiana*	This study	N/A
**Recombinant DNA**		
PBI121-HAM3-GFP-2xGME2	This study	10.6019/S-BSST2877
PBI121-MBY49-GFP-2xGME2	This study	10.6019/S-BSST2877
PBI121-PCR2-GFP-2xGME2	This study	10.6019/S-BSST2877
PBI121-HAM3-3WJ-4×Bro-2xGME2	This study	10.6019/S-BSST2877
PBI121-MBY49-3WJ-4×Bro-2xGME2	This study	10.6019/S-BSST2877
PBI121-PCR2-3WJ-4×Bro-2xGME2	This study	10.6019/S-BSST2877
**Oligonucleotides and other sequence-based reagents**		
*TCTP1*forward(5’-3’): CACGGGGGACTCTAGAATGTTGGTGTACCAAGATCTTCTCA	This study	N/A
*TCTP1*-reverse(5’-3’): CATACACATGACAAGGTACCGCACTTGACCTCCTTCAAACCA	This study	N/A
*ACTIN2*-forward(5’-3’): CACGGGGGACTCTAGAATGGCTGAGGCTGATGATATTC	This study	N/A
*ACTIN2*-reverse(5’-3’): CATACACATGACAAGGTACCGAAACATTTTCTGTGAACGATTCC	This study	N/A
Kan-forward(5’-3’): CAGGATCTCCTGTCATCTCACC	This study	N/A
Kan-reverse(5’-3’): CAACGCTATGTCCTGATAGCGG	This study	N/A
4B-forward(5’-3’): CGGGGGACTCTAGAGGTACCTTG	This study	N/A
4B-reverse(5’-3’): CGAGCTCCGGCCGCCAGTGTGATGGATA	This study	N/A
*NtACT97*-forward(5’-3’): CTTACCGAGCGTGGTTACAT	This study	N/A
*NtACT97*-reverse(5’-3’): GAAGAGCTGGTCTTTGCAGT	This study	N/A
16S-forward(5’-3’): AGGTGAAATTCGTAGATATTCGGAGGAAC	This study	N/A
16S-reverse(5’-3’): GACGGCTAACATTCATCGTTTACG	This study	N/A
GME1 nucleotide sequence(5’-3’): CTTAGACCAAGTGAGATTGAATCTGTTGCG	This study	N/A
GME2 nucleotide sequence(5’-3’): ATGGTGGCTCAATGAATTGATCTGTTGAAC	This study	N/A
3WJ-4×Bro nucleotide sequence(5’-3’) CTTGTCATGTGTATGTTGGGGAGACGGTCGGGTCCAGATATTCGTATCTGTCGAGTAGAGTGTGGGCTCCCCACATACTTTGTTGACCGAGACGGTCGGGTCCAGATATTCGTATCTGTCGAGTAGAGTGTGGGCTCGGTCAATCATGGCAAGGATCCACTAGTAACGGCCGCCAGTGTGCTGGAATTCTTGTCATGTGTATGTTGGGGAGACGGTCGGGTCCAGATATTCGTATCTGTCGAGTAGAGTGTGGGCTCCCCACATACTTTGTTGACCGAGACGGTCGGGTCCAGATATTCGTATCTGTCGAGTAGAGTGTGGGCTCGGTCAATCATGGCAAGATATCCATCACACTGGCGGCCG	This study	N/A
TLS(tRNA^Met^) nucleotide sequence(5’-3’) AACAAAGCACCAGTGGTCTAGTGGTAGAATAGTACCCTGCCACGGTACAGACCCGGGTTCGATTCCCGGCTGGTGCA	This study	N/A
**Chemicals, enzymes, and other reagents**		
DFHBI-1T	MEC	CAS: 1539318-36-9
EDTA-2Na	Solarbio	CAS: 6381-92-6
Silwet L-77	Merck	Cat#S9430
In-Fusion Snap Assembly Master Mix	Takara	Cat#638947
MES	Euromedex	Cat# EU0033
DMSO	Sigma-Aldrich	Cat# D4540
Propidium iodide Staining Solution (PI staining)	Yeasen	Cat# 25535-16-4
**Software**		
ImageJ	https://imagej.nih.gov/	
Graphpad Prism 8	https://www.graphpad.com/	
Adobe Photoshop CS5	https://www.adobe.com	
Adobe Illustrator	https://www.adobe.com/	
**Other**		
Leica M205 FA	Leica	
Olympus SpinFV-COMB	Olympus	

### Plasmids construction

To generate targeted transcripts fused with the 2×GME2 RNA delivery system, reporter genes 3WJ-4×Bro and GFP were used. The mRNA sequences of *TCTP1* (At3G16640), *CAT3* (AT1G20620), *CK1* (AT1G71697), *GAI* (AT1G14920), *AtMYB49* (AT5G54230), *AtHMA3* (AT4G30120), and *AtPCR2* (AT1G14870) were amplified from the cDNA library and cloned into the pBI121 vector using an In-Fusion cloning kit. All the constructed vectors were verified using Sanger sequencing.

### Plant materials and growth conditions

Plant materials used in this study included *Arabidopsis thaliana* (Col-0) and *Nicotiana benthamiana*. *Arabidopsis thaliana* ecotype Columbia-0 (Col-0) and *Nicotiana benthamiana* seeds used in this study were maintained in our laboratory. Seeds of *N. benthamiana* and *A. thaliana* were sown in potting soil with a nutrient soil to vermiculite ratio of 2:1 and placed in a growth chamber for cultivation. The growth conditions included a light intensity of 100–150 μmol/m^2^·s, a photoperiod of 16 h light and 8 h dark, a temperature of 23 °C during the light period and 20 °C during the dark period, and a relative humidity of 70%.

### Expression of reporter RNA in *N. benthamiana* leaves

The recombinant pBI121 binary plasmids were transformed into *Agrobacterium tumefaciens* strain GV3101 via chemical transformation. Following the method described by Bai et al (2020), the transformed bacterial suspension was infiltrated into *N. benthamiana* leaves. Fluorescence signals were detected using a confocal microscope at 48 h post-infiltration.

### Fluorescence in situ hybridization (FISH)

For probe design, five probes targeting various sites of the *TCTP*-3WJ-4×Bro fused transcript were designed using Stellaris® Probe Designer version 2.0 from Biosearch Technologies (http://singlemoleculefish.com). Before ordering pre-labeled probes from Biosearch Technologies, an NCBI BLAST query was run for each sequence to ensure target specificity (https://blast.ncbi.nlm.nih.gov/Blast.cgi). The oligonucleotides of each probe, labeled with Cy3 at the 5′ terminus, were synthesized by Sangon Biotech Co., Ltd. The *TCTP1*-3WJ-4×Bro construct was transiently expressed in *N. benthamiana* via agroinfiltration. At 56 h post-agroinfiltration, the stem sections and root tissues were incubated with 10 μM DFHBI-1T via infiltration for 5 min and 30 min, respectively. Following the incubation, the excess dye liquid was blotted away. Fluorescent signals were immediately detected using a confocal microscope (objective HCX PL APO CS 20.0 × 0.70 DRY UV, excitation 488 nm, emission 527 nm). Following fluorescence imaging, stem sections and root tissues of the *N. benthamiana* were immediately immersed in a fixative solution prepared with DEPC-treated water and fixed for a minimum of 12 h. Post-fixation, the samples underwent dehydration through a graduated ethanol series, then were embedded in paraffin under vacuum conditions using a vacuum pump. These paraffin-embedded specimens were sectioned with a microtome, with the sections collected on a slide warmer. The slides were then incubated at 62 °C for 2 h. Following incubation, the sections underwent deparaffinization in two sequential clearing solutions for 15 min each, followed by two 5-mine rinses in absolute ethanol. After air-drying, the sections were rehydrated in DEPC-treated water. Digestion with 20 μg/mL Proteinase K (Target Mol, USA, T8936) was conducted at 37 °C for a specified time. The sections were then rinsed in water, followed by three 5-min PBS washes. Prehybridization solution was applied and incubated at 37 °C for 1 h, after which it was removed. Hybridization proceeded overnight at 37 °C using a Cy3-labeled probe-specific hybridization solution at 6 ng/μL. Post hybridization, the sections were washed with 2×SSC at 37 °C for 10 min, twice with 1×SSC at 37 °C for 5 min each, and once with 0.5×SSC at room temperature for 10 min, with the option to add formamide washes for high background signal reduction. Examination and imaging of the samples were performed using the fluorescence microscope. ImageJ software was utilized for analyzing the co-localization of the signal from the probes with the 3WJ-4×Bro fluorescence.

### Transformation of *Arabidopsis thaliana*

Agrobacterium GV3101 carrying recombinant plasmids was cultured to OD_600_ = 1.6 (28 °C, 180 rpm), centrifuged, and resuspended in infiltration medium (5% sucrose and 0.02% Silwet L-77) to OD_600_ = 1.0 for 1 h incubation in darkness. Inflorescences of wild-type *Arabidopsis thaliana* plants (Col-0) were dipped twice in the Agrobacterium bacterial suspension (10 s each) and then covered with plastic bags. After 16 h of dark incubation, plants were transferred to a growth chamber (16-h light/8-h dark cycle). T_0_ seeds were selected on kanamycin-containing MS medium (30 μg/mL). Two-week-old kanamycin-resistant seedlings were grown for 10 days either in soil or on MS medium, followed by RT–PCR analysis with 35S/NOS primer pairs and fluorescence imaging using a confocal laser scanning microscope.

### Micrografting of *Arabidopsis thaliana* plants

*A. thaliana* seeds were vertically grown on plates containing 1/2 MS salts, 1% sucrose, and 1% micro agar under short-day conditions in a controlled environmental chamber (Percival) for 7–8 days. Seedlings of similar size and with evenly elongated hypocotyls were cut in the upper half of the hypocotyl with a sterilized razor blade. The junctions of the graft were supported using silicon microtubing (0.3 mm internal diameter). Grafted plants were then transferred to new plates and grown vertically under short-day conditions. Adventitious roots that formed above the junction were removed every two days (Yang et al, 2019). Two weeks after grafting, 3WJ-4×Bro fluorescence was detected in the scions and stocks of the plants via CLSM. The scion and root tissues were harvested for RNA isolation and detection of transcripts by RT–PCR using oligo(dT) primers for RT–PCR and specific primers for PCR amplification.

### Time-dependent imaging of long-distance mRNA movement

An equivalent amount of *Agrobacterium tumefaciens* solution was infiltrated into the leaf epidermal cells of 3-week-old *N. benthamiana* plants with uniform growth. After 30 h of infiltration, the samples of the root region (the root region located 145 mm from the agroinfiltration site) were taken every hour. The root tissues were spread flat on glass slides and incubated with 10 μM DFHBI-1T for 30 min in the dark, and then immediately subjected to fluorescence imaging using an Olympus SpinSR10 super-resolution confocal microscope to detect the 3WJ-4×Bro signal. The sampling and detection methods were consistent for each trial.

### Quantification of mobility rate and transport rate

Twenty independent plants were examined for each construct at each time point under identical confocal imaging settings in the predefined distal regions. A plant was scored as fluorescence-positive when a clear 3WJ-4×Bro signal was detected in the designated distal region. The mobility rates were calculated as the percentage of fluorescence-positive plants among the total number of plants examined. The transport rate was defined as the distance from the inoculation site to the designated distal region divided by the time after inoculation at which the first fluorescence-positive plant was detected in that region.

### Ion content analysis

Wild-type (WT) and transgenic *A. thaliana* seedlings were cultured on 1/2 MS medium for 7 days, after which uniformly grown seedlings were selected for micrografting using silicone support collars. Grafted seedlings with established vascular connections were transferred to 1/2 MS medium supplemented with either 200 mM NaCl, 20 μM Cd, or 300 μM Zn for stress treatment. Following treatment, plants were immersed in 1 mM EDTA solution for 30 min, rinsed five times with distilled water, and then separated into leaves and roots before drying at 65 °C for 3 days. The contents of Ca²⁺, Cd²⁺, and Zn²⁺ were determined by ICP-MS (iCAP Q, Thermo Fisher Scientific, Waltham, MA, USA).

### Determination of leaf relative water content and electrolyte leakage

Three graft combinations (WT/WT, WT/*pUBQ: AtMYB49*, and WT/*pUBQ: AtMYB49-2×GME2*) were treated with 200 mM NaCl for 14 days. The relative water content (RWC) of scion leaves was determined following Negrão et al (2017). Electrolyte leakage was quantified according to Nishiyama et al (2011) by comparing pre-boiling (C₁) and post-boiling (C₂) conductivity measurements, with electrolyte leakage calculated as (C₁/C₂) × 100%.

### Statistical analysis

All statistical analyses were performed using GraphPad Prism. Data were presented as means ± SD. *n* represents independent biological replicates. Statistical significance between two groups was assessed using unpaired two-tailed Student’s *t* test (Oh et al, 2025).

## Supplementary information

Table EV1

Peer Review File

Source data Fig. 1

Source data Fig. 2

Source data Fig. 3 part 1

Source data Fig. 3 part 2

Source data Fig. 3 Part 3

Source data Fig. 4 part 1

Source data Fig. 4 part 2

Source data Fig. 5 part 1

Source data Fig. 5 part 2

Source data Fig. 6

Expanded View Figures

## Data Availability

All data needed to evaluate the conclusions of this study are present in the paper or the Supplementary Materials. The source data for this paper have been collected in the BioStudies database record (https://www.ebi.ac.uk/biostudies/bioimages/studies/S-BIAD3150?key=e6706cc3-6415-403f-b585-42c0a47bf8d9). The sequence information for all of the constructs has been deposited in the BioStudies database record (https://www.ebi.ac.uk/biostudies/studies/S-BSST2877?key=0ca6e59d-88dd-433f-b392-062196ed4bf6). The source data of this paper are collected in the following database record: biostudies:S-SCDT-10_1038-S44319-026-00819-z.

## References

[CR1] Akhiyarova G, Finkina EI, Zhang K, Veselov D, Vafina G, Ovchinnikova TV, Kudoyarova G (2024) The long-distance transport of some plant hormones and possible involvement of lipid-binding and transfer proteins in hormonal transport. Cells 13:1–16 10.3390/cells13050364 PMC10931013 38474328

[CR2] Albacete A, Martínez-Andújar C, Martínez-Pérez A, Thompson AJ, Dodd IC, Pérez-Alfocea F (2015) Unravelling rootstock×scion interactions to improve food security. J Exp Bot 66:2211–2226 25754404 10.1093/jxb/erv027 PMC4986720

[CR3] Ariga H, Toki S, Ishibashi K (2020) Potato Virus X vector-mediated DNA-free genome editing in plants. Plant Cell Physiol 61:1946–1953 32991731 10.1093/pcp/pcaa123 PMC7758033

[CR4] Bai J, Luo Y, Wang X, Li S, Luo M, Yin M, Zuo Y, Li G, Yao J, Yang H et al (2020) A protein-independent fluorescent RNA aptamer reporter system for plant genetic engineering. Nat Commun 11:1–14 32737299 10.1038/s41467-020-17497-7 PMC7395781

[CR5] Bakirbas A, Castro-Rodriguez R, Walker EL (2023) The small RNA component of *Arabidopsis thaliana* phloem sap and its response to iron deficiency. Plants 12:1–13 10.3390/plants12152782 PMC10421156 37570935

[CR6] Banerjee AK, Chatterjee M, Yu Y, Suh S-G, Miller WA, Hannapel DJ (2006) Dynamics of a mobile RNA of potato involved in a long-distance signaling pathway. Plant Cell 18:3443–3457 17189340 10.1105/tpc.106.042473 PMC1785412

[CR7] Bletsos FA (2006) Grafting and calcium cyanamide as alternatives to methyl bromide for greenhouse eggplant production. Sci Hortic 107:325–331

[CR8] Carbonnel S, Cornelis S, Hazak O (2023) The CLE33 peptide represses phloem differentiation via autocrine and paracrine signaling in *Arabidopsis*. Commun Biol 6:1–9 37280369 10.1038/s42003-023-04972-2 PMC10244433

[CR9] Cho SK, Sharma P, Butler NM, Kang IH, Shah S, Rao AG, Hannapel DJ (2015) Polypyrimidine tract-binding proteins of potato mediate tuberization through an interaction with *StBEL5* RNA. J Exp Bot 66:6835–6847 26283046 10.1093/jxb/erv389 PMC4623692

[CR10] Colleoni PE, van Es SW, Winkelmolen T, Immink RGH, van Esse GW (2024) Flowering time genes branching out. J Exp Bot 75:4195–4209 38470076 10.1093/jxb/erae112 PMC11263490

[CR11] Ellison EE, Nagalakshmi U, Gamo ME, Huang P-J, Dinesh-Kumar SP, Voytas DF (2020) Multiplexed heritable gene editing using RNA viruses and mobile single guide RNAs. Nat Plants 6:620–624 32483329 10.1038/s41477-020-0670-y

[CR12] Feng M, Augstein F, Kareem A, Melnyk CW (2024) Plant grafting: molecular mechanisms and applications. Mol Plant 17:75–91 38102831 10.1016/j.molp.2023.12.006

[CR13] Fu M, Xu Z, Ma H, Hao Y, Tian J, Wang Y, Zhang X, Xu X, Han Z, Wu T (2024) Characteristics of long-distance mobile mRNAs from shoot to root in grafted plant species. Hortic Plant J 10:25–37

[CR14] Hao P, Lv X, Fu M, Xu Z, Tian J, Wang Y, Zhang X, Xu X, Wu T, Han Z (2022) Long-distance mobile mRNA *CAX3* modulates iron uptake and zinc compartmentalization. EMBO Rep 23:e53698 35254714 10.15252/embr.202153698 PMC9066076

[CR15] Haywood V, Yu TS, Huang NC, Lucas WJ (2005) Phloem long-distance trafficking of *GIBBERELLIC ACID-INSENSITIVE* RNA regulates leaf development. Plant J 42:49–68 15773853 10.1111/j.1365-313X.2005.02351.x

[CR16] Heeney M, Frank MH (2023) The mRNA mobileome: challenges and opportunities for deciphering signals from the noise. Plant Cell 35:1817–1833 36881847 10.1093/plcell/koad063 PMC10226602

[CR17] Huang NC, Yu TS (2009) The sequences of Arabidopsis *GA-INSENSITIVE* RNA constitute the motifs that are necessary and sufficient for RNA long-distance trafficking. Plant J 59:921–929 19453448 10.1111/j.1365-313X.2009.03918.x

[CR18] Itzkovitz S, van Oudenaarden A (2011) Validating transcripts with probes and imaging technology. Nat Methods 8:S12–S19 21451512 10.1038/nmeth.1573 PMC3158979

[CR19] Kang B, Lee S, Ko DH, Venkatesh J, Kwon JK, Kim H, Kang BC (2025) Virus-induced systemic and heritable gene editing in pepper (*Capsicum annuum* L.). Plant J 122:e70257 40499557 10.1111/tpj.70257 PMC12158543

[CR20] Kehr J, Morris RJ, Kragler F (2022) Long-distance transported RNAs: from identity to function. Annu Rev Plant Biol 73:457–474 34910585 10.1146/annurev-arplant-070121-033601

[CR21] Kitagawa M, Tran TM, Jackson D (2024) Traveling with purpose: cell-to-cell transport of plant mRNAs. Trends Cell Biol 34:48–57 37380581 10.1016/j.tcb.2023.05.010

[CR22] Li C, Gu M, Shi N, Zhang H, Yang X, Osman T, Liu Y, Wang H, Vatish M, Jackson S et al (2011) Mobile *FT* mRNA contributes to the systemic florigen signalling in floral induction. Sci Rep 1:1–6 22355592 10.1038/srep00073 PMC3216560

[CR23] Li C, Zhang K, Zeng X, Jackson S, Zhou Y, Hong Y (2009) A cis element within *flowering locus T* mRNA determines its mobility and facilitates trafficking of heterologous viral RNA. J Virol 83:3540–3548 19193810 10.1128/JVI.02346-08 PMC2663265

[CR24] Li T, Hu J, Sun Y, Li B, Zhang D, Li W, Liu J, Li D, Gao C, Zhang Y et al (2021) Highly efficient heritable genome editing in wheat using an RNA virus and bypassing tissue culture. Mol Plant 14:1787–1798 34274523 10.1016/j.molp.2021.07.010

[CR25] Li XJ, Wang CC, Chen Y, Liu WQ, Zhang M, Wang NN, Xiang CG, Gao LH, Dong YH, Zhang WN (2025) m5C and m6A modifications regulate the mobility of pumpkin *CHOLINE KINASE 1* mRNA under chilling stress. Plant Physiol 197:kiae511 39325727 10.1093/plphys/kiae511

[CR26] Loupit G, Brocard L, Ollat N, Cookson SJ (2023) Grafting in plants: recent discoveries and new applications. J Exp Bot 74:2433–2447 36846896 10.1093/jxb/erad061

[CR27] Lucas WJ, Bouché-Pillon S, Jackson DP, Nguyen L, Baker L, Ding B, Hake S (1995) Selective trafficking of KNOTTED1 homeodomain protein and its mRNA through plasmodesmata. Science 270:1980–1983 8533088 10.1126/science.270.5244.1980

[CR28] Ma X, Zhang X, Liu H, Li Z (2020) Highly efficient DNA-free plant genome editing using virally delivered CRISPR–Cas9. Nat Plants 6:773–779 32601419 10.1038/s41477-020-0704-5

[CR29] Morel M, Crouzet J, Gravot A, Auroy P, Leonhardt N, Vavasseur A, Richaud P (2009) *AtHMA3*, a P1B-ATPase allowing Cd/Zn/Co/Pb vacuolar storage in Arabidopsis. Plant Physiol 149:894–904 19036834 10.1104/pp.108.130294 PMC2633814

[CR30] Negrão S, Schmöckel SM, Tester M (2017) Evaluating physiological responses of plants to salinity stress. Ann Bot 119:1–11 27707746 10.1093/aob/mcw191 PMC5218372

[CR31] Nishiyama R, Watanabe Y, Fujita Y, Le DT, Kojima M, Werner T, Vankova R, Yamaguchi-Shinozaki K, Shinozaki K, Kakimoto T et al (2011) Analysis of cytokinin mutants and regulation of cytokinin metabolic genes reveals important regulatory roles of cytokinins in drought, salt and abscisic acid responses, and abscisic acid biosynthesis. Plant Cell 23:2169–2183 21719693 10.1105/tpc.111.087395 PMC3160038

[CR32] Oh Y, Nagalakshmi U, Dahlbeck D, Koehler N, Cho MJ, Dinesh-Kumar SP, Staskawicz BJ (2025) Heritable virus-induced germline editing in tomato. Plant J 122:e70115 40163287 10.1111/tpj.70115 PMC11956848

[CR33] Paajanen P, Tomkins M, Hoerbst F, Veevers R, Heeney M, Thomas HR, Apelt F, Saplaoura E, Gupta S, Frank MH et al (2025) Re-analysis of mobile mRNA datasets raises questions about the extent of long-distance mRNA communication. Nat Plants 11:977–984 40240650 10.1038/s41477-025-01979-x PMC12095074

[CR34] Panth M, Hassler SC, Baysal-Gurel F (2020) Methods for management of soilborne diseases in crop production. Agriculture 10:1–21

[CR35] Qin Y, Yang C, Xia J, He J, Ma X, Yang C, Zheng Y, Lin X, He Z, Huang Z et al (2014) Effects of dual/threefold rootstock grafting on the plant growth, yield and quality of watermelon. Not Bot Horti Agrobot Cluj Napoca 42:495–500

[CR36] Song WY, Choi KS, Kim DY, Geisler M, Park J, Vincenzetti V, Schellenberg M, Kim SH, Lim YP, Noh EW et al (2010) Arabidopsis *PCR2* is a zinc exporter involved in both zinc extrusion and long-distance zinc transport. Plant Cell 22:2237–2252 20647347 10.1105/tpc.109.070185 PMC2929092

[CR37] Thieme CJ, Rojas-Triana M, Stecyk E, Schudoma C, Zhang W, Yang L, Miñambres M, Walther D, Schulze WX, Paz-Ares J et al (2015) Endogenous Arabidopsis messenger RNAs transported to distant tissues. Nat Plants 1:1–9 10.1038/nplants.2015.25 27247031

[CR38] Wang L, Liao Y, Liu J, Zhao T, Jia L, Chen Z (2024) Advances in understanding the graft healing mechanism: a review of factors and regulatory pathways. Hortic Res 11:1–13 10.1093/hr/uhae175 PMC11301322 39108577

[CR39] Wang YQ (2011) Plant grafting and its application in biological research. Chin Sci Bull 56:3511–3517

[CR40] Xiao Y, Chen Y-M, Zou Z, Ye C, Dou X, Wu J, Liu C, Liu S, Yan H, Wang P et al (2024) Profiling of RNA-binding protein binding sites by in situ reverse transcription-based sequencing. Nat Methods 21:247–258 38200227 10.1038/s41592-023-02146-w PMC10864177

[CR41] Yang L, Machin F, Wang S, Saplaoura E, Kragler F (2023) Heritable transgene-free genome editing in plants by grafting of wild-type shoots to transgenic donor rootstocks. Nat Biotechnol 41:958–967 36593415 10.1038/s41587-022-01585-8 PMC10344777

[CR42] Yang L, Perrera V, Saplaoura E, Apelt F, Bahin M, Kramdi A, Olas J, Mueller-Roeber B, Sokolowska E, Zhang W et al (2019) m(5)C methylation guides systemic transport of messenger RNA over graft junctions in plants. Curr Biol 29:2465–2476.e5 31327714 10.1016/j.cub.2019.06.042

[CR43] Yoshida T, Ishikawa M, Toki S, Ishibashi K (2024) Heritable tissue-culture-free gene editing in *Nicotiana benthamiana* through viral delivery of SpCas9 and sgRNA. Plant Cell Physiol 65:1743–1750 39215594 10.1093/pcp/pcae100 PMC11631083

[CR44] Zhang DH, Meng ZH, Xiao WM, Wang XC (2002) Graft-induced inheritable variation in mungbean and its application in mungbean breeding. Acta Bot Sin 44:832–837

[CR45] Zhang M, Liu W, Wang C, Lin S, Chen Y, Cui H, Xiang C, Ma Y, Li X, Lu Y et al (2025) Root-to-shoot mobile mRNA CmoKARI1 promotes JA-Ile biosynthesis to confer chilling tolerance in grafted cucumbers. Nat Commun 16:1–16 40835858 10.1038/s41467-025-63228-1 PMC12368190

[CR46] Zhang P, Wang R, Yang X, Ju Q, Li W, Lü S, Tran LP, Xu J (2020) The R2R3-MYB transcription factor *AtMYB49* modulates salt tolerance in Arabidopsis by modulating cuticle formation and antioxidant defence. Plant Cell Environ 43:1925–1943 32406163 10.1111/pce.13784

[CR47] Zhang W, Thieme CJ, Kollwig G, Apelt F, Yang L, Winter N, Andresen N, Walther D, Kragler F (2016) tRNA-related sequences trigger systemic mRNA transport in plants. Plant Cell 28:1237–1249 27268430 10.1105/tpc.15.01056 PMC4944404

[CR48] Zheng HX, Sun X, Zhang XS, Sui N (2020) m6A editing: new tool to improve crop quality? Trends Plant Sci 25:859–867 32376086 10.1016/j.tplants.2020.04.005

