# Supplementary figures and images for: Developing a mobile RNA delivery system for grafting trait improvement
Source: EMBO Rep. 2026 Jun 10;27(13):3863–89. doi: 10.1038/s44319-026-00819-z (PMC13354801; doi:10.1038/s44319-026-00819-z)

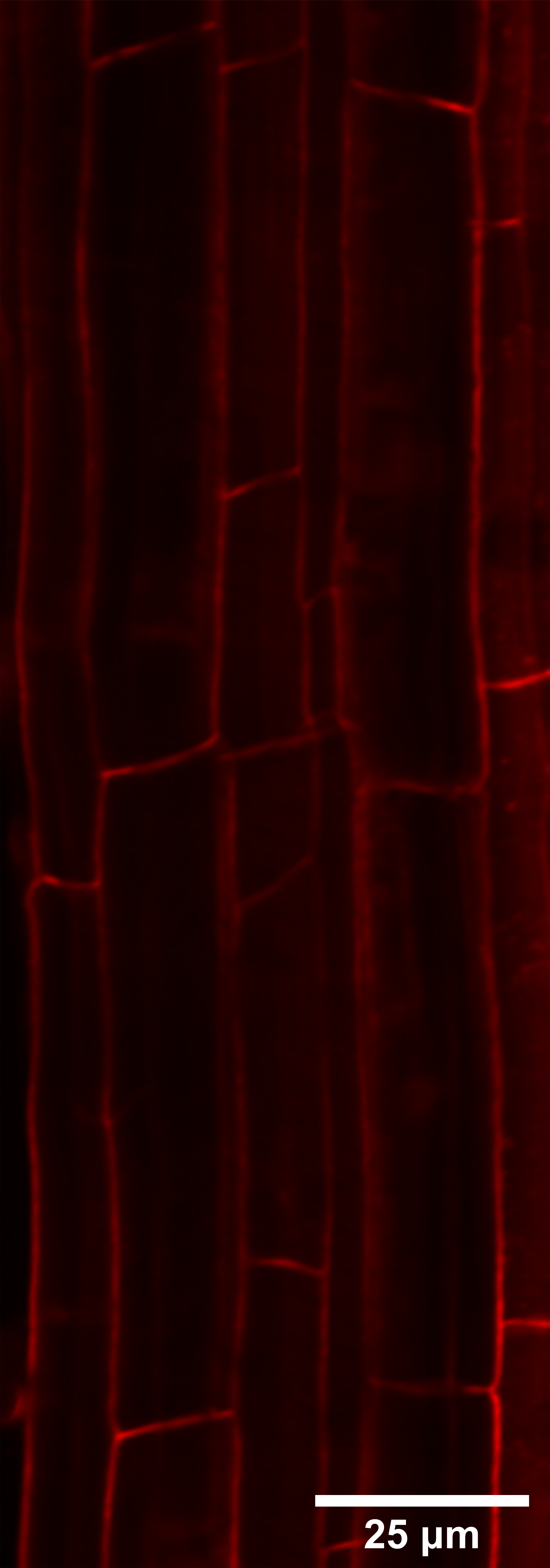

Supplement: Supplementary file 3 — Source data Fig. 1 [file 44319_2026_819_MOESM3_ESM.zip › FiG1/1B/Region 2/3WJ-4xBro.tif]

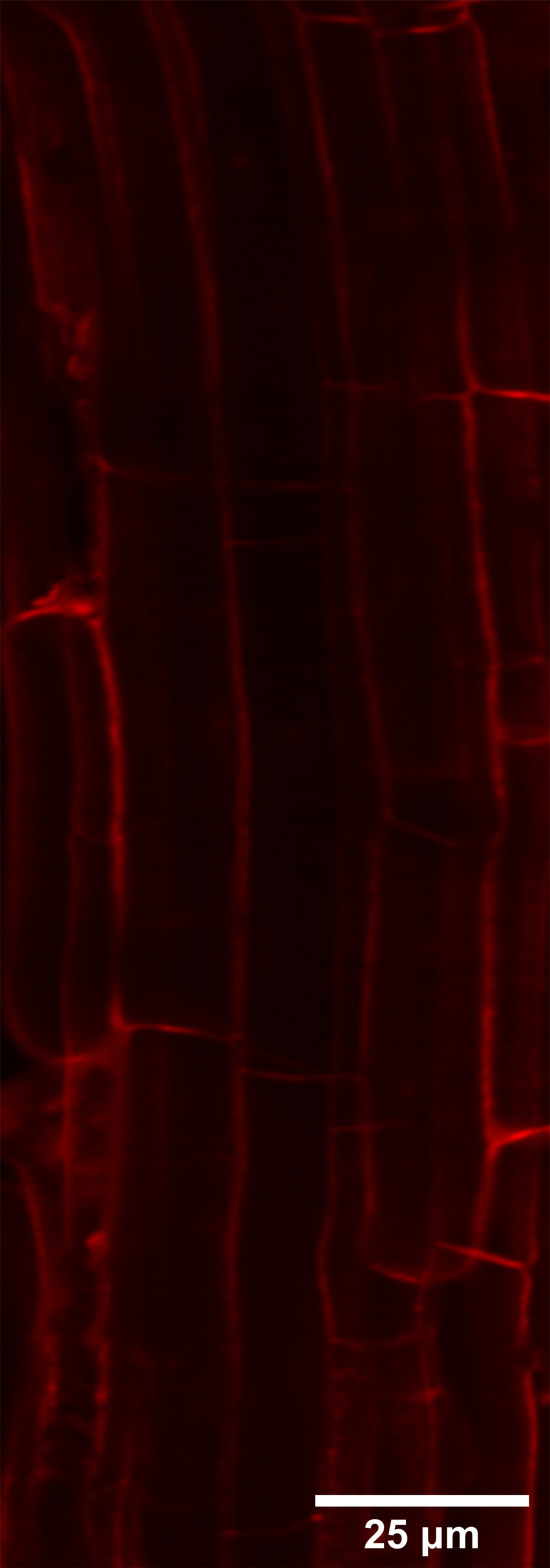

Supplement: Supplementary file 3 — Source data Fig. 1 [file 44319_2026_819_MOESM3_ESM.zip › FiG1/1B/Region 2/ACTIN2-3WJ-4xBro.tif]

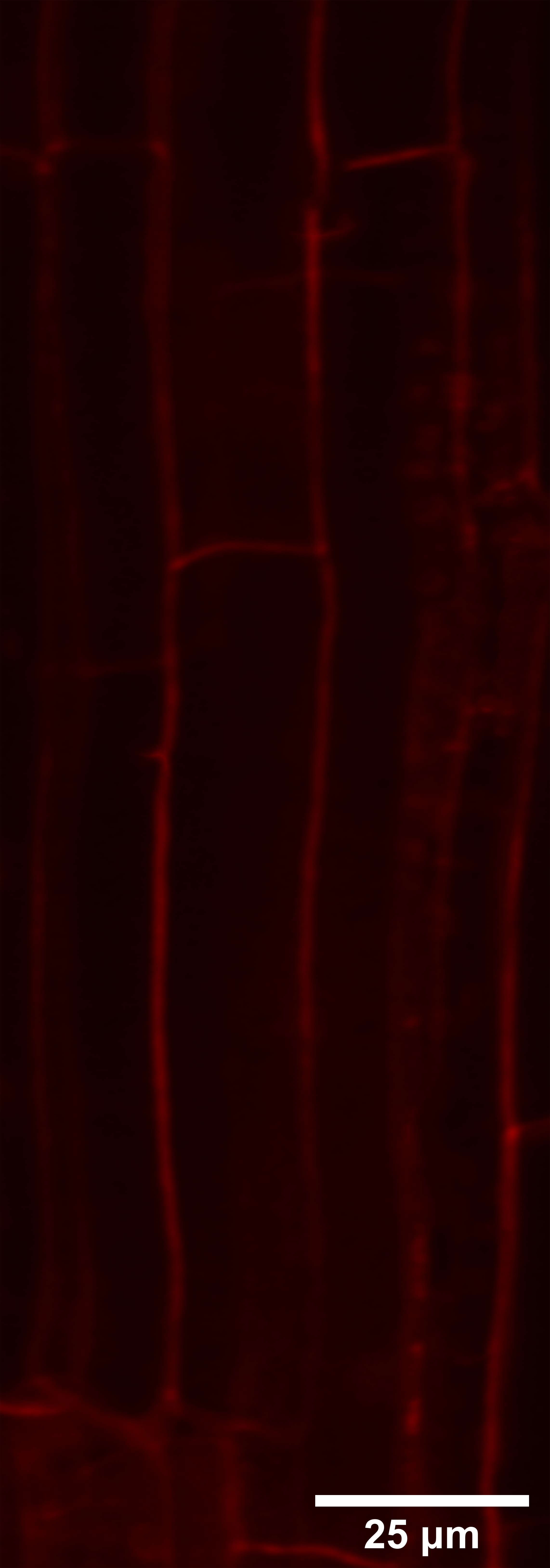

Supplement: Supplementary file 3 — Source data Fig. 1 [file 44319_2026_819_MOESM3_ESM.zip › FiG1/1B/Region 2/DFHBI.tif]

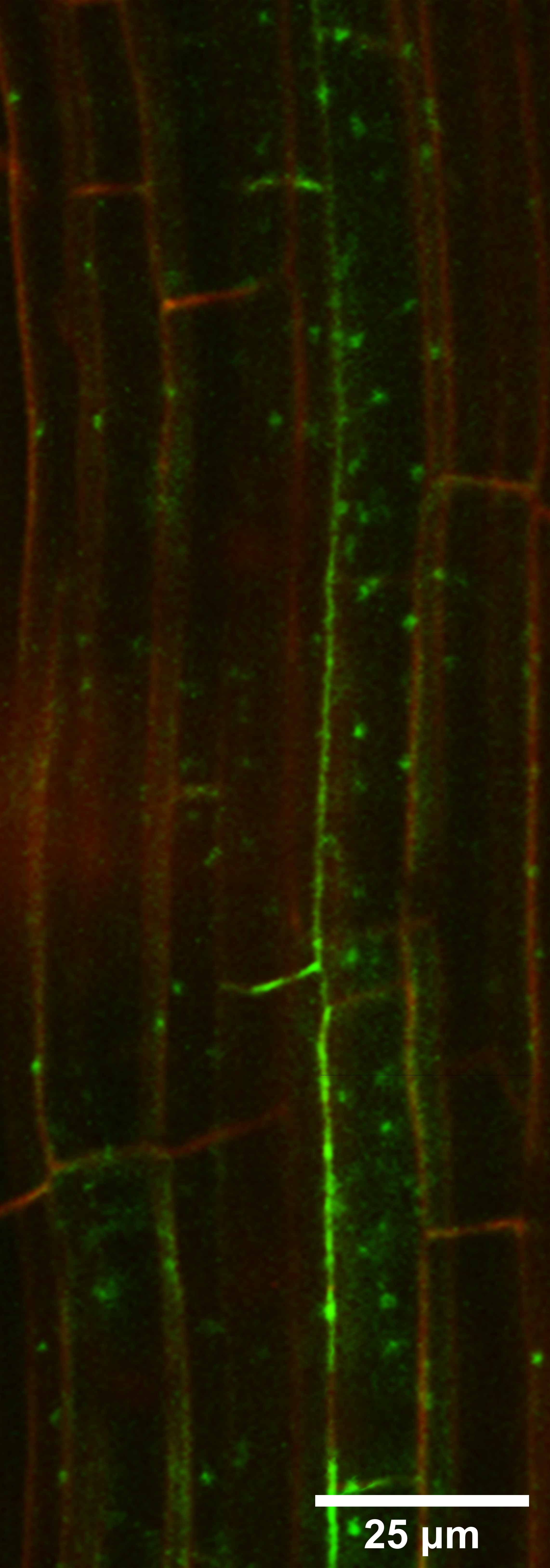

Supplement: Supplementary file 3 — Source data Fig. 1 [file 44319_2026_819_MOESM3_ESM.zip › FiG1/1B/Region 2/TCTP1-3WJ-4xBro.tif]

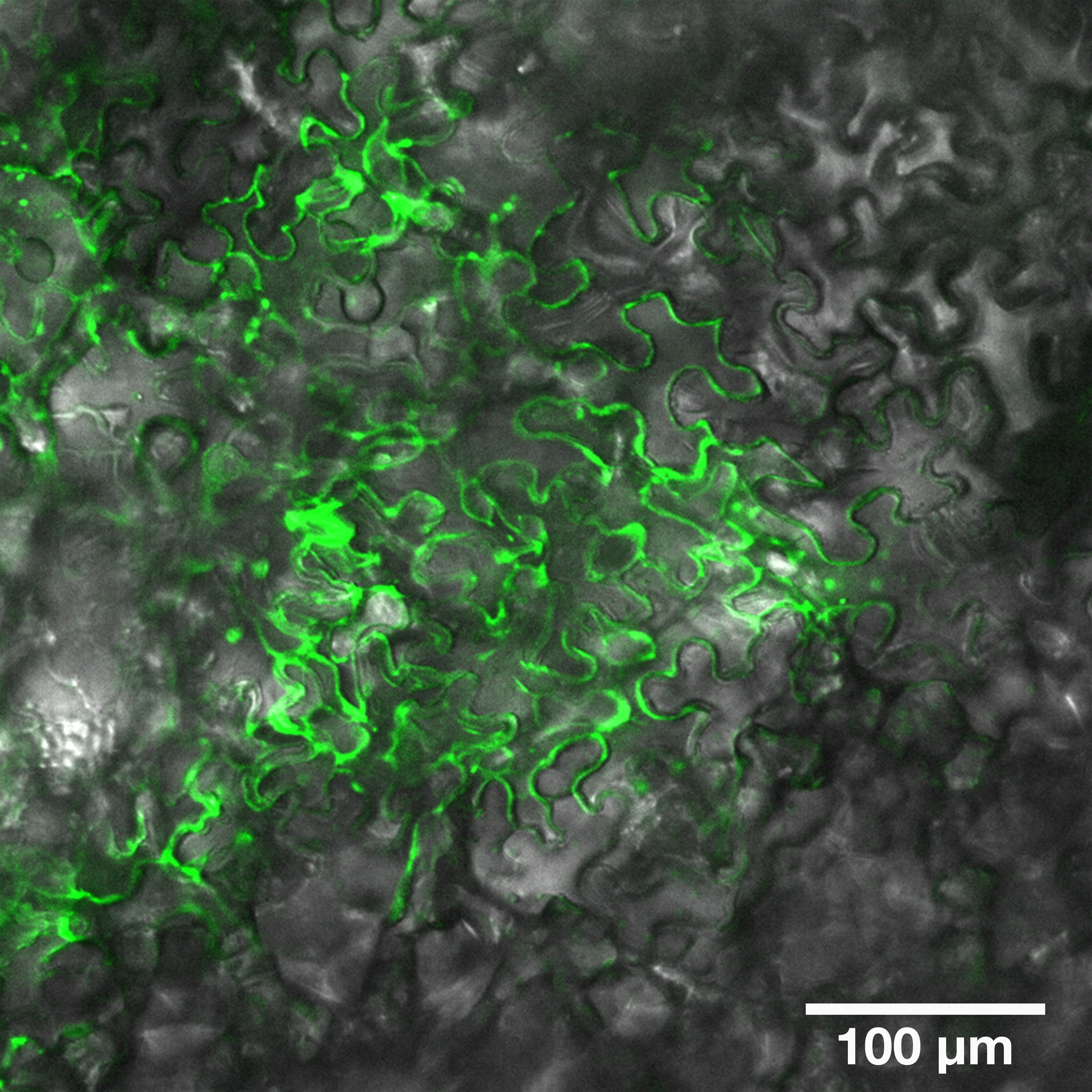

Supplement: Supplementary file 3 — Source data Fig. 1 [file 44319_2026_819_MOESM3_ESM.zip › FiG1/1B/Region1/3WJ-4×Bro.png]

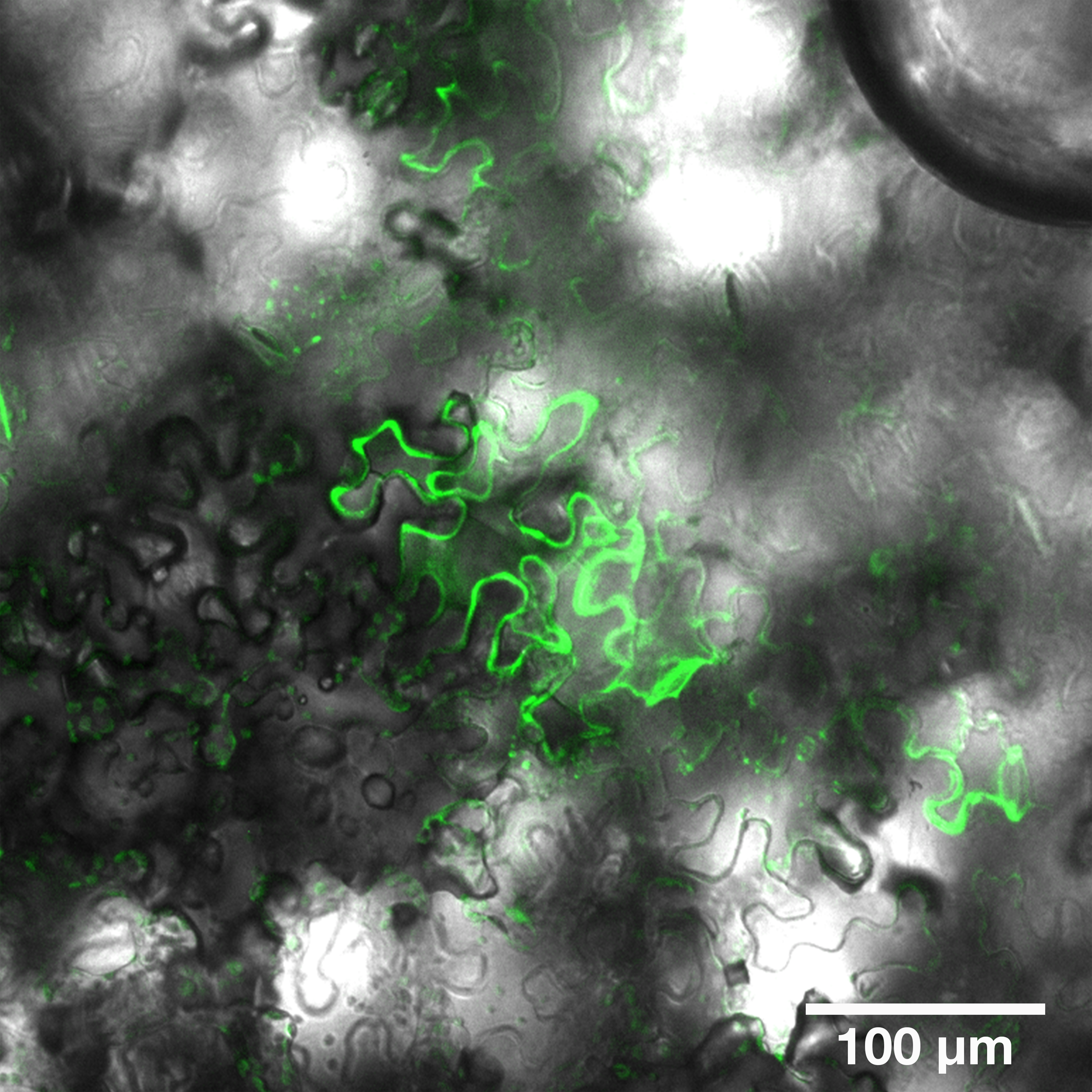

Supplement: Supplementary file 3 — Source data Fig. 1 [file 44319_2026_819_MOESM3_ESM.zip › FiG1/1B/Region1/ACTIN2-3WJ-4×Bro.png]

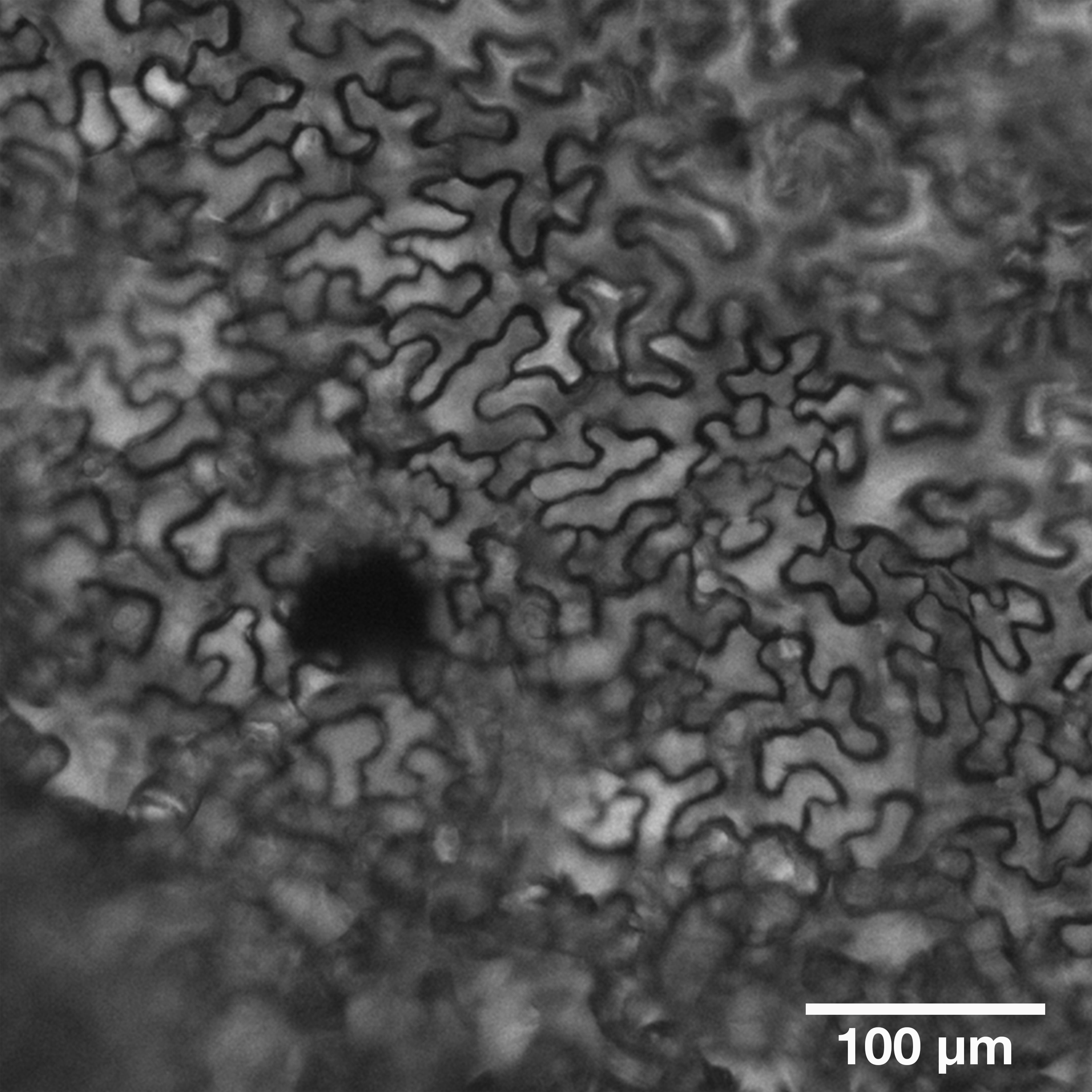

Supplement: Supplementary file 3 — Source data Fig. 1 [file 44319_2026_819_MOESM3_ESM.zip › FiG1/1B/Region1/DFHBI.png]

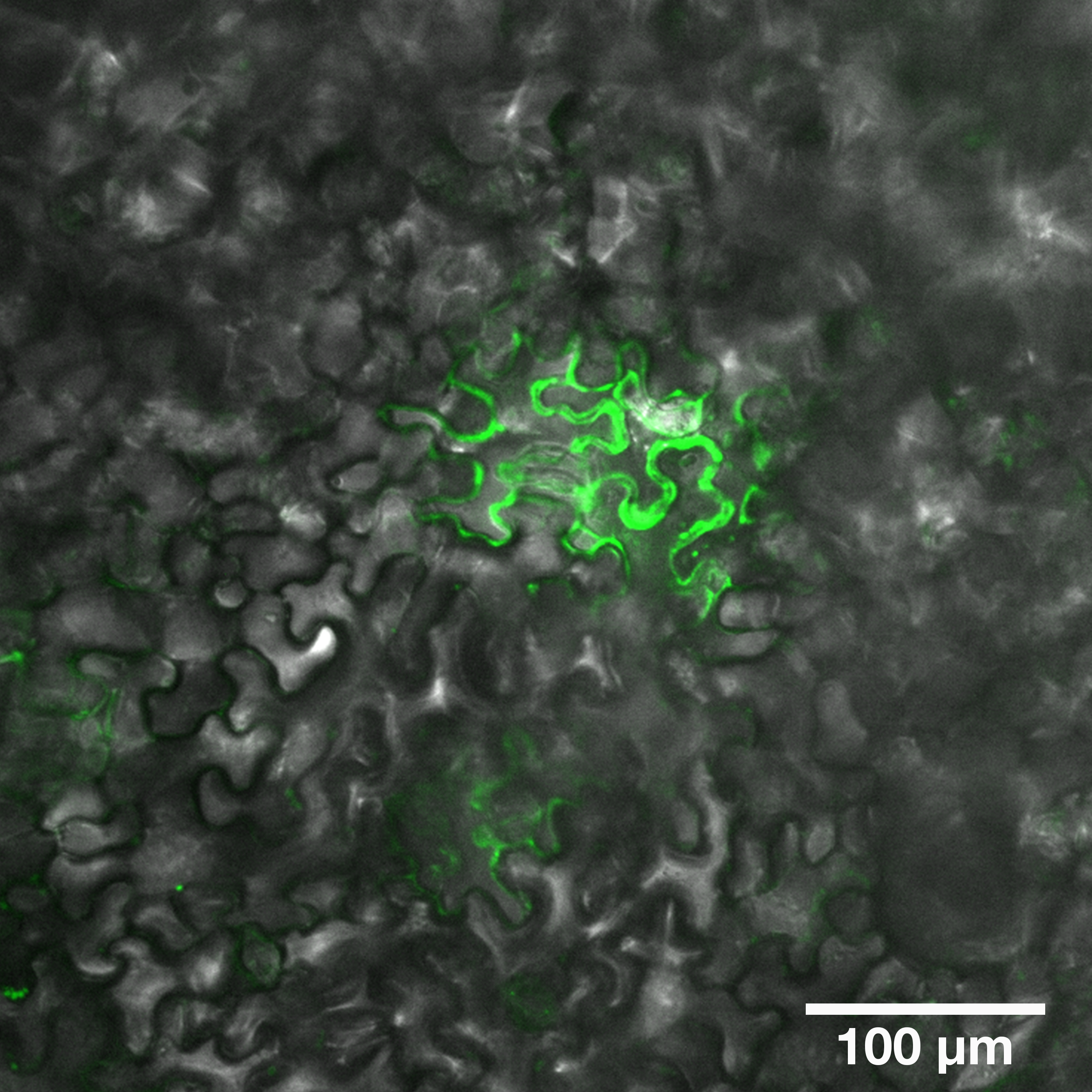

Supplement: Supplementary file 3 — Source data Fig. 1 [file 44319_2026_819_MOESM3_ESM.zip › FiG1/1B/Region1/TCTP1-3WJ-4×Bro.png]

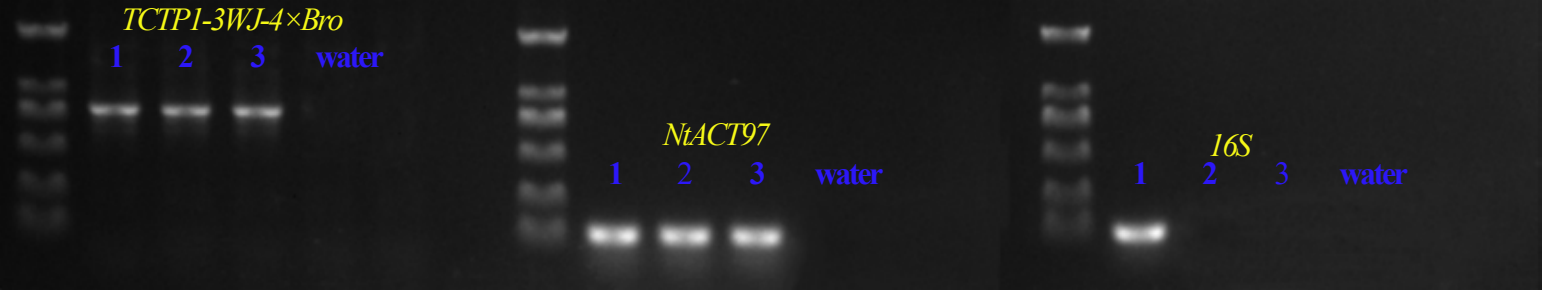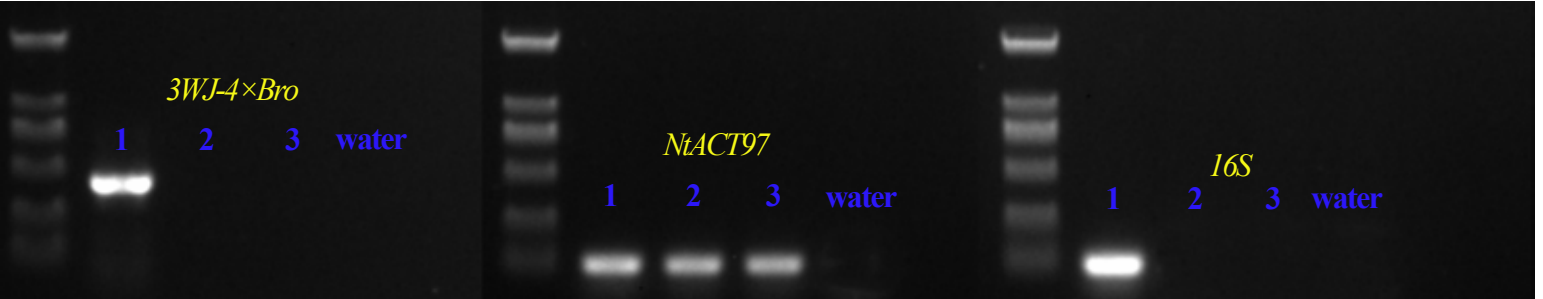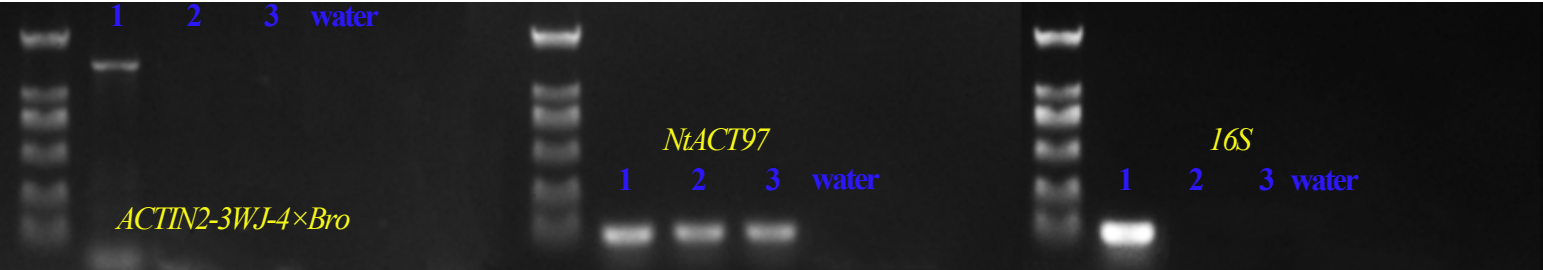

Supplement: Supplementary file 3 — Source data Fig. 1 [file 44319_2026_819_MOESM3_ESM.zip › FiG1/1C/胶图.pdf]

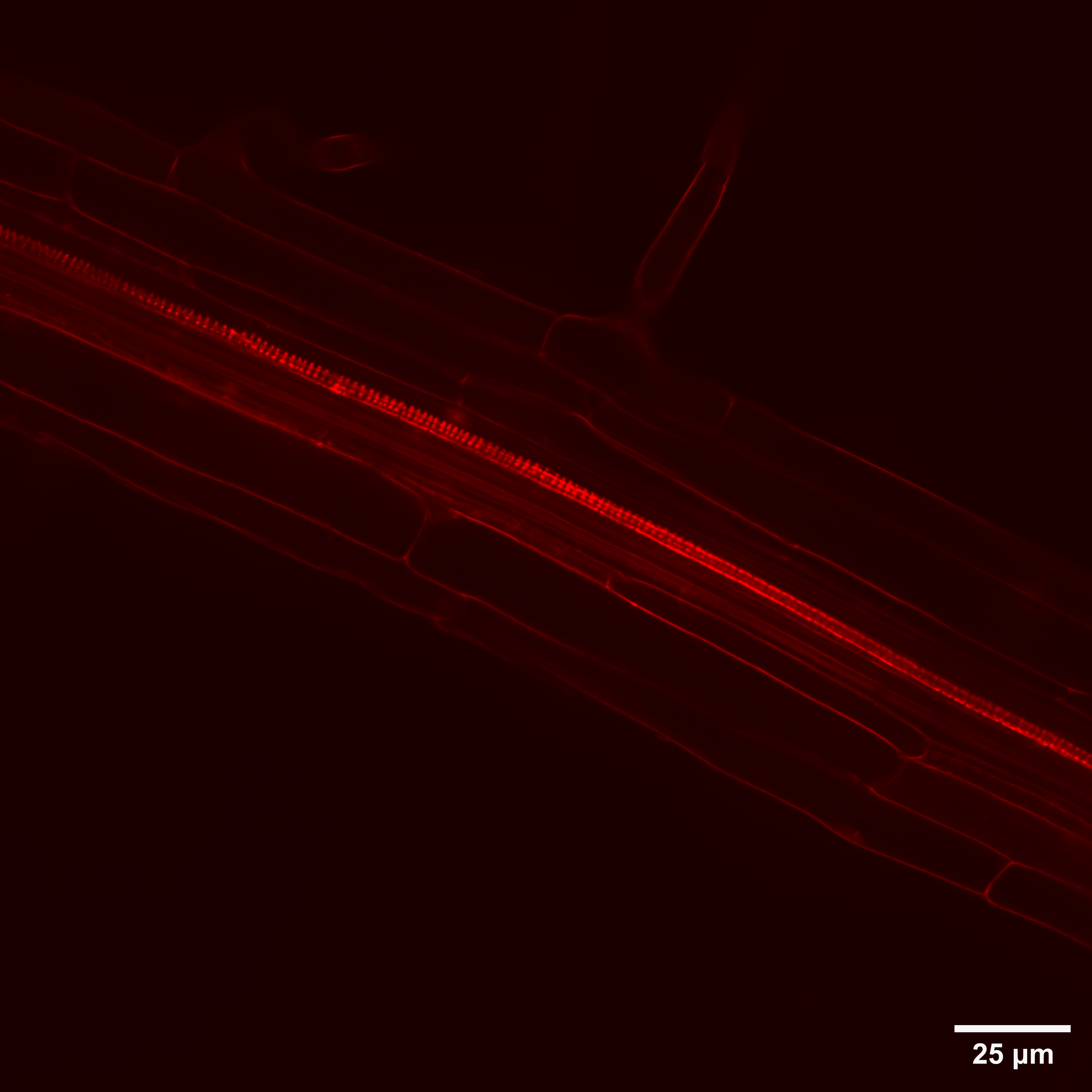

Supplement: Supplementary file 3 — Source data Fig. 1 [file 44319_2026_819_MOESM3_ESM.zip › FiG1/1D/3WJ-4xBro-1.png]

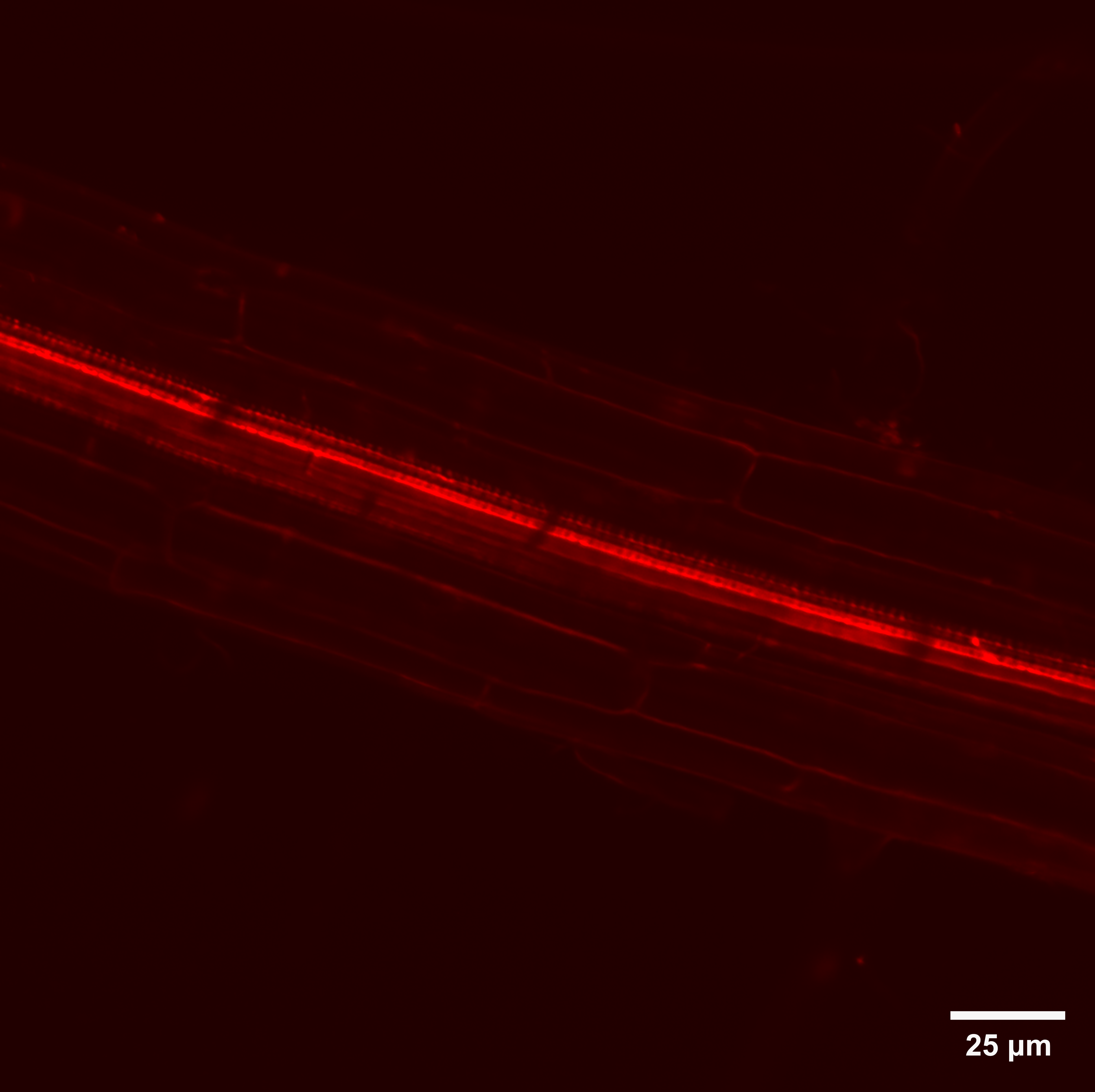

Supplement: Supplementary file 3 — Source data Fig. 1 [file 44319_2026_819_MOESM3_ESM.zip › FiG1/1D/3WJ-4xBro-2.png]

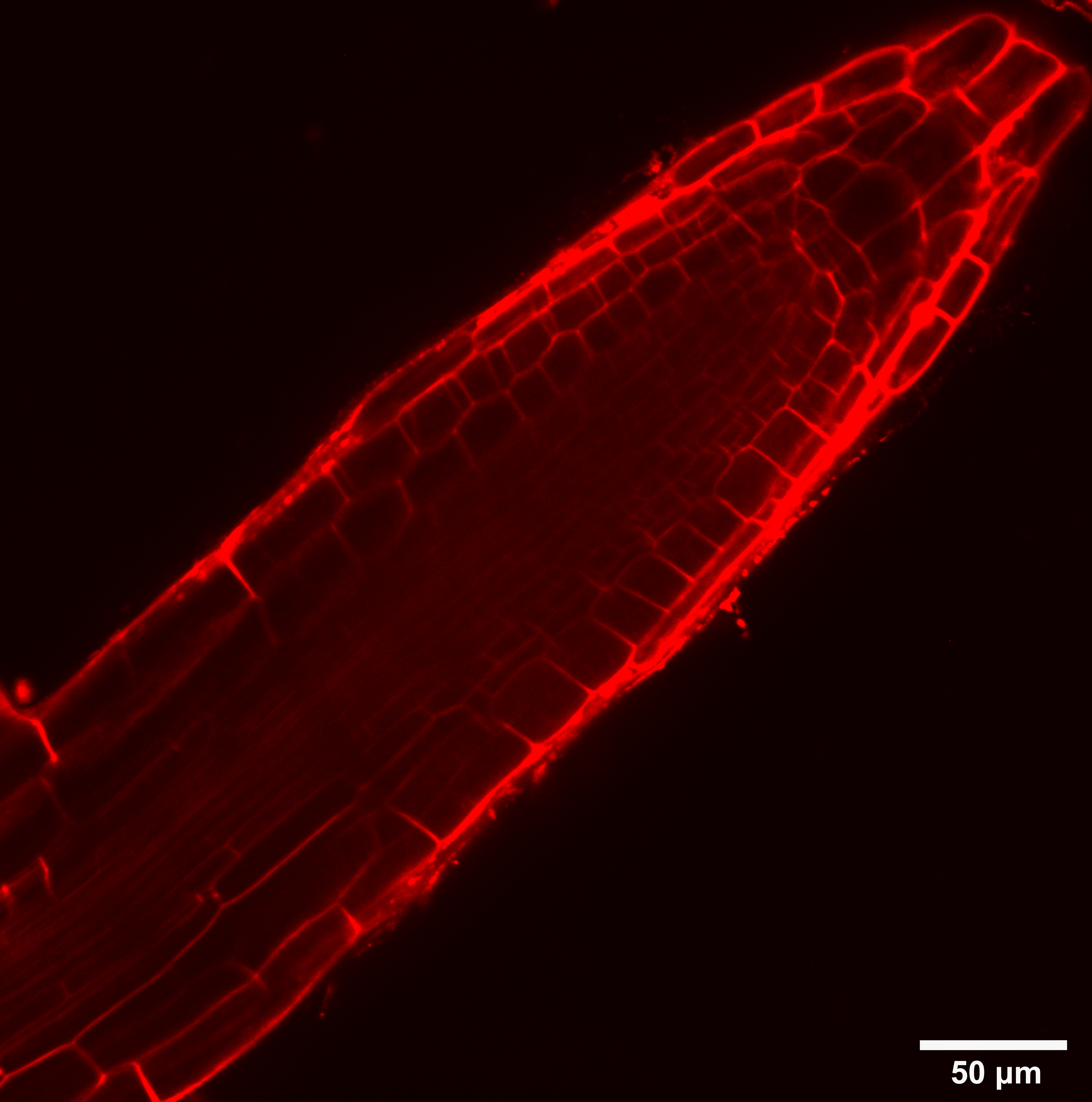

Supplement: Supplementary file 3 — Source data Fig. 1 [file 44319_2026_819_MOESM3_ESM.zip › FiG1/1D/3WJ-4xBro.png]

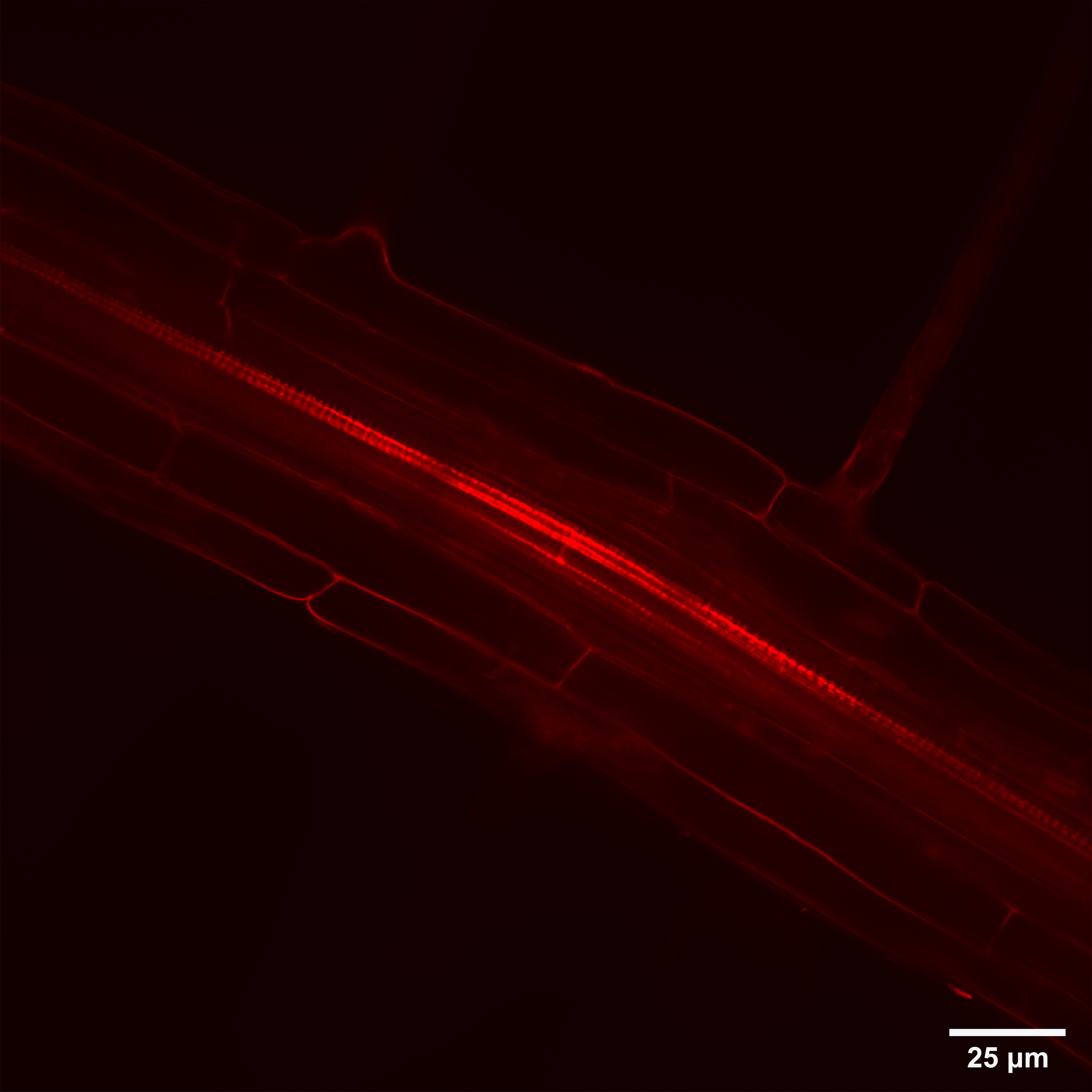

Supplement: Supplementary file 3 — Source data Fig. 1 [file 44319_2026_819_MOESM3_ESM.zip › FiG1/1D/ACTIN2-3WJ-4xBro-1.png]

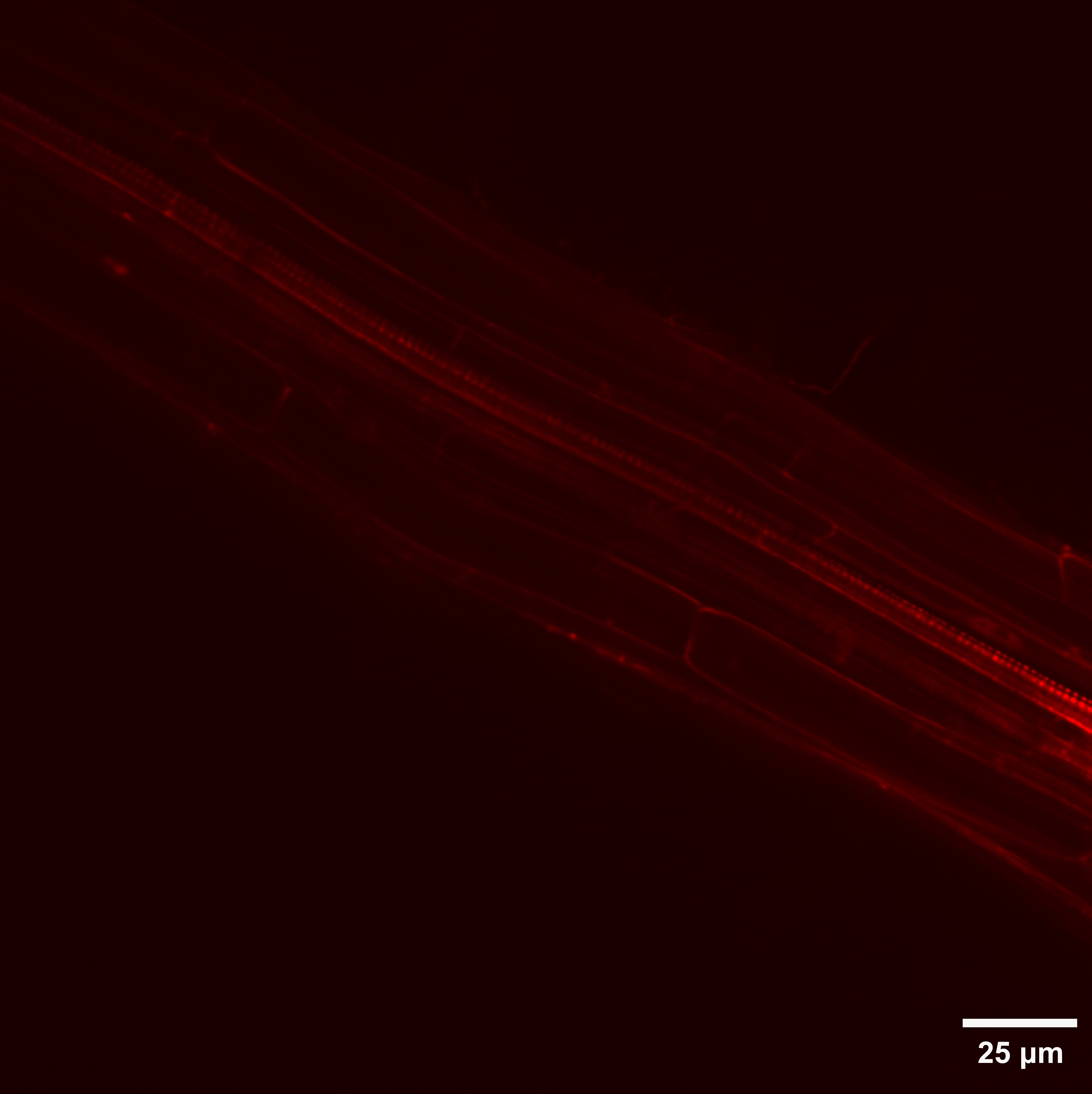

Supplement: Supplementary file 3 — Source data Fig. 1 [file 44319_2026_819_MOESM3_ESM.zip › FiG1/1D/ACTIN2-3WJ-4xBro-2.png]

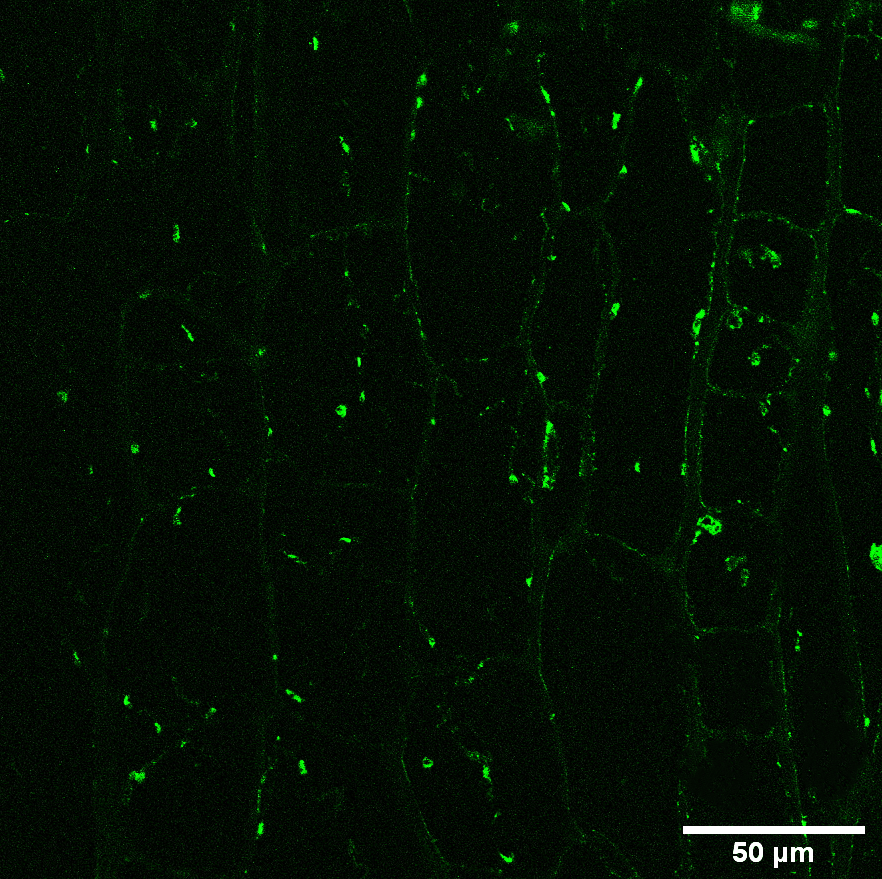

Supplement: Supplementary file 3 — Source data Fig. 1 [file 44319_2026_819_MOESM3_ESM.zip › FiG1/1E/3WJ-4xBro-Root.tif]

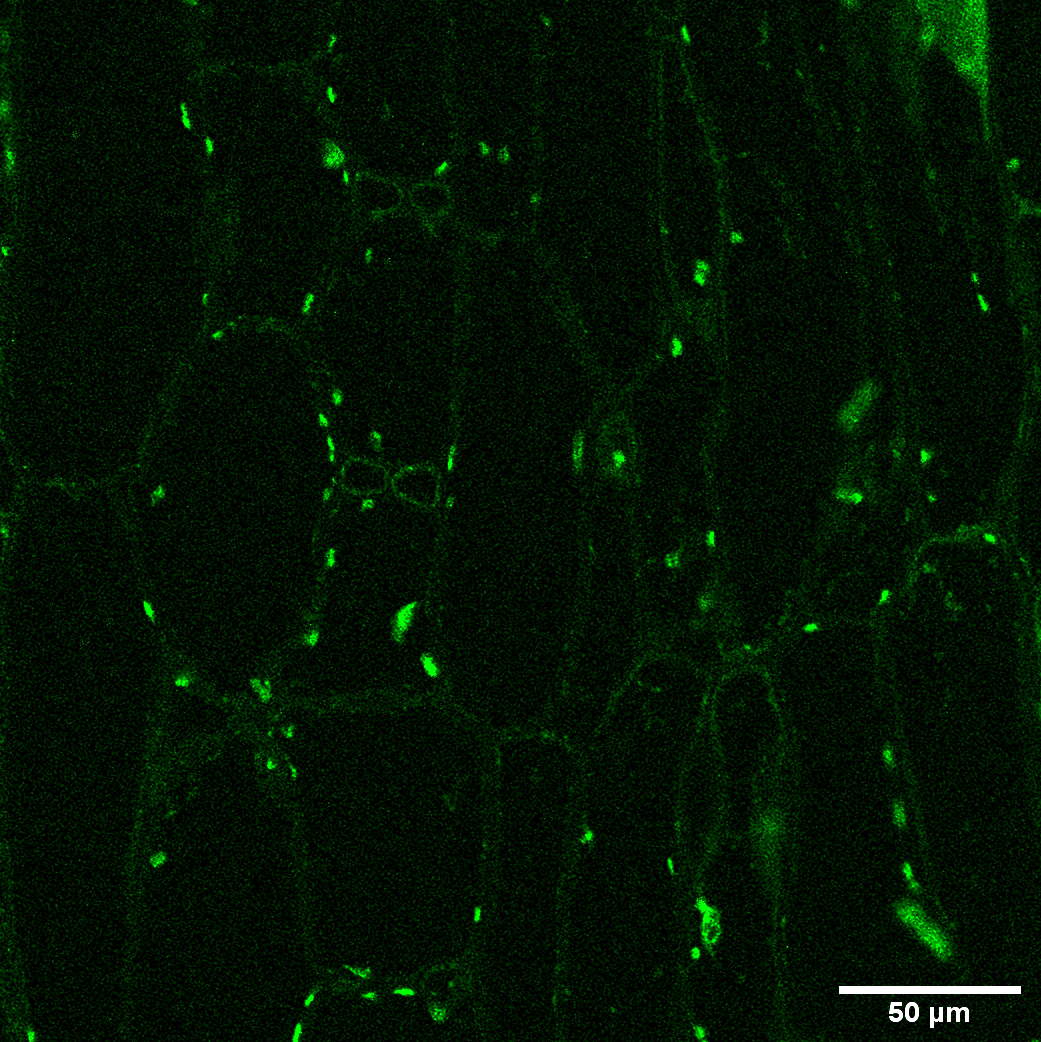

Supplement: Supplementary file 3 — Source data Fig. 1 [file 44319_2026_819_MOESM3_ESM.zip › FiG1/1E/3WJ-4xBro-Stem.tif]

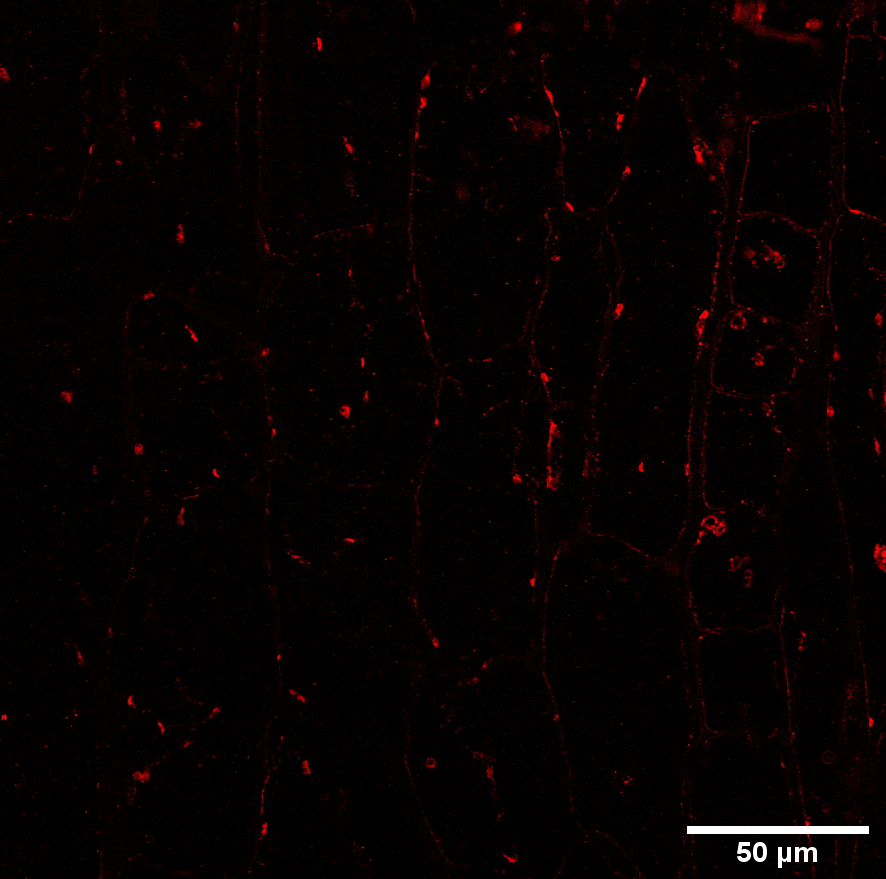

Supplement: Supplementary file 3 — Source data Fig. 1 [file 44319_2026_819_MOESM3_ESM.zip › FiG1/1E/FISH-Root.tif]

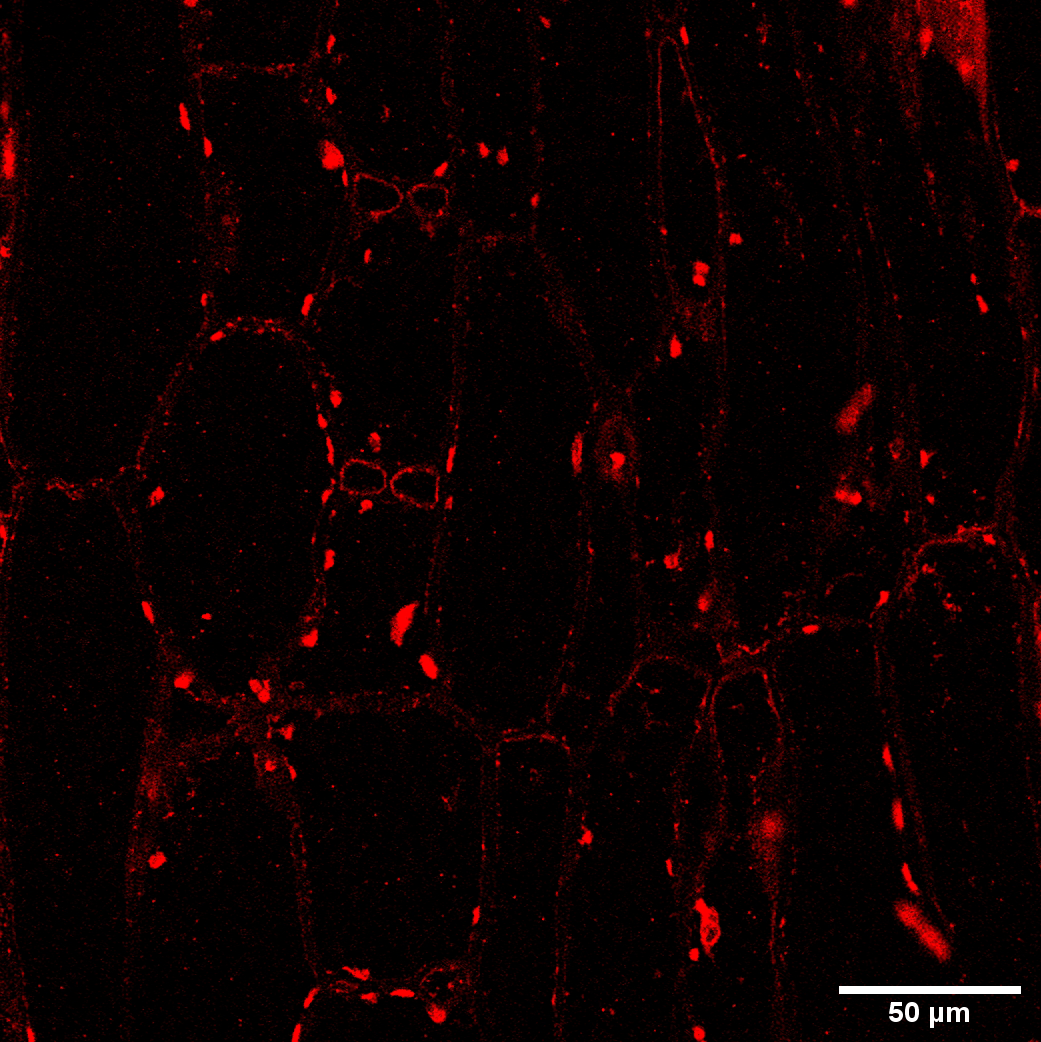

Supplement: Supplementary file 3 — Source data Fig. 1 [file 44319_2026_819_MOESM3_ESM.zip › FiG1/1E/FISH-Stem.tif]

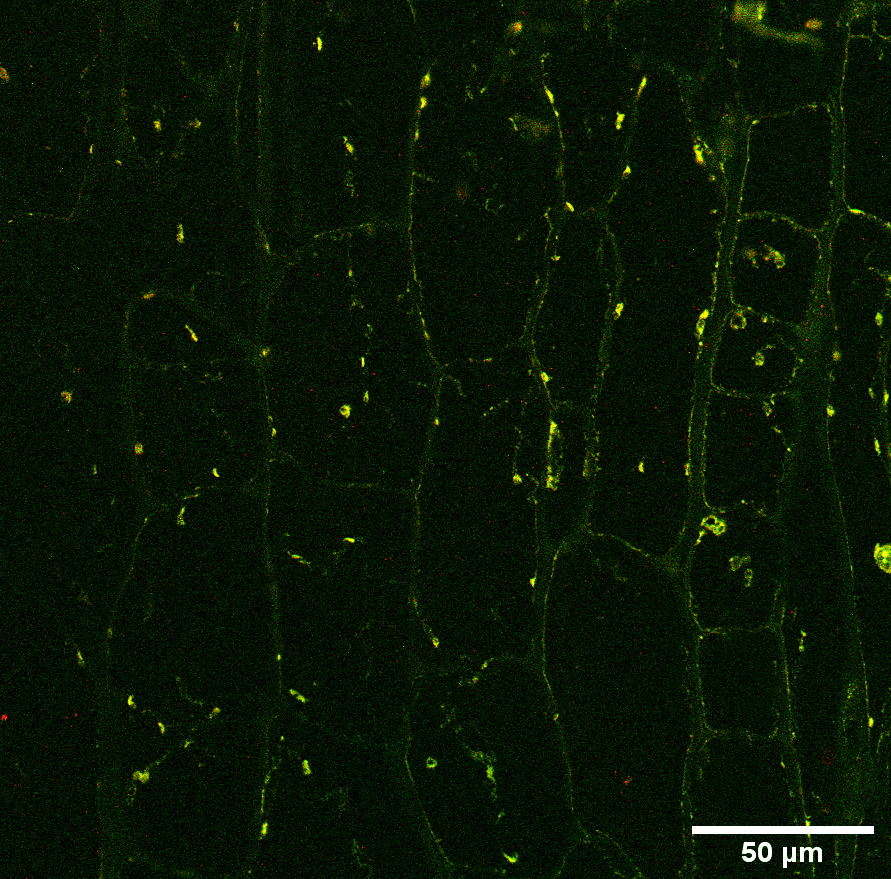

Supplement: Supplementary file 3 — Source data Fig. 1 [file 44319_2026_819_MOESM3_ESM.zip › FiG1/1E/Merge-Root.tif]

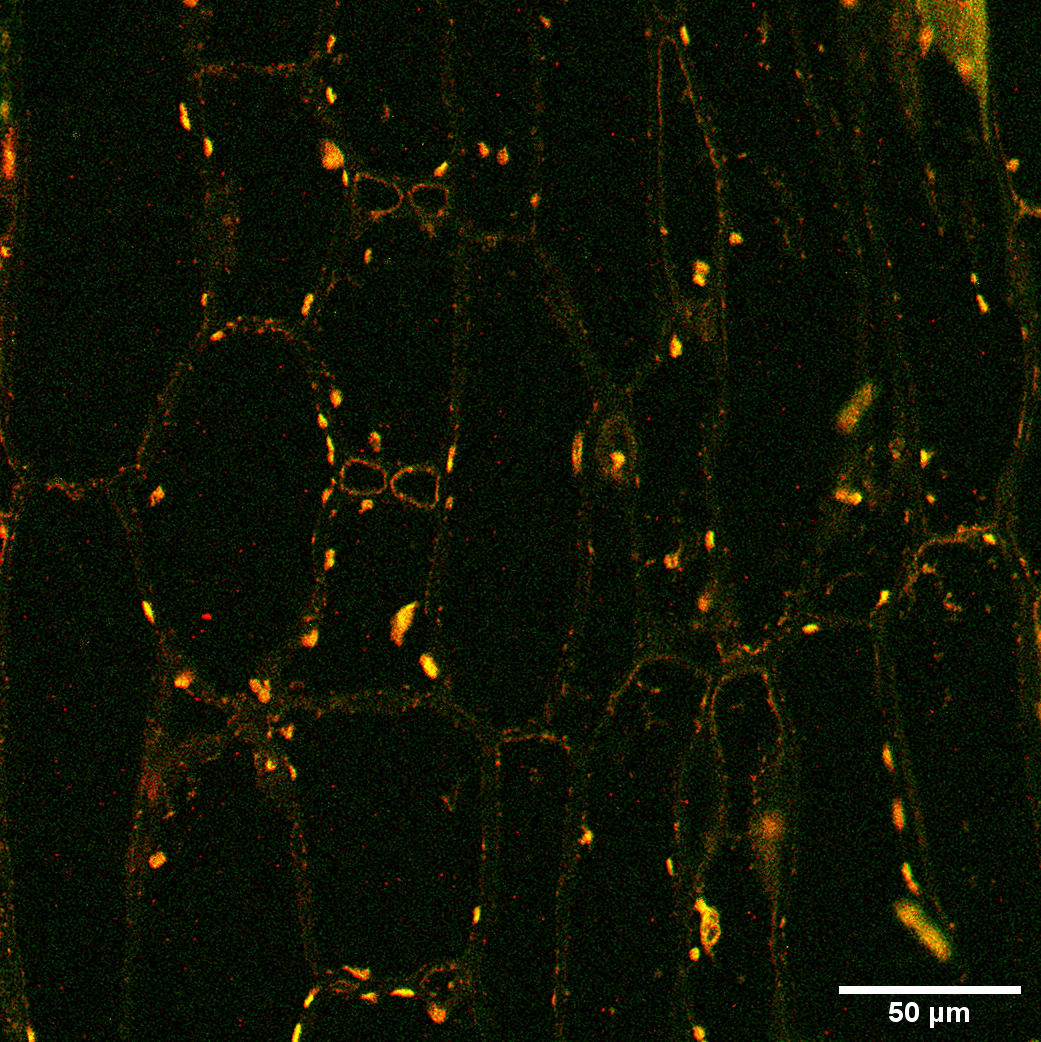

Supplement: Supplementary file 3 — Source data Fig. 1 [file 44319_2026_819_MOESM3_ESM.zip › FiG1/1E/Merge-Stem.tif]

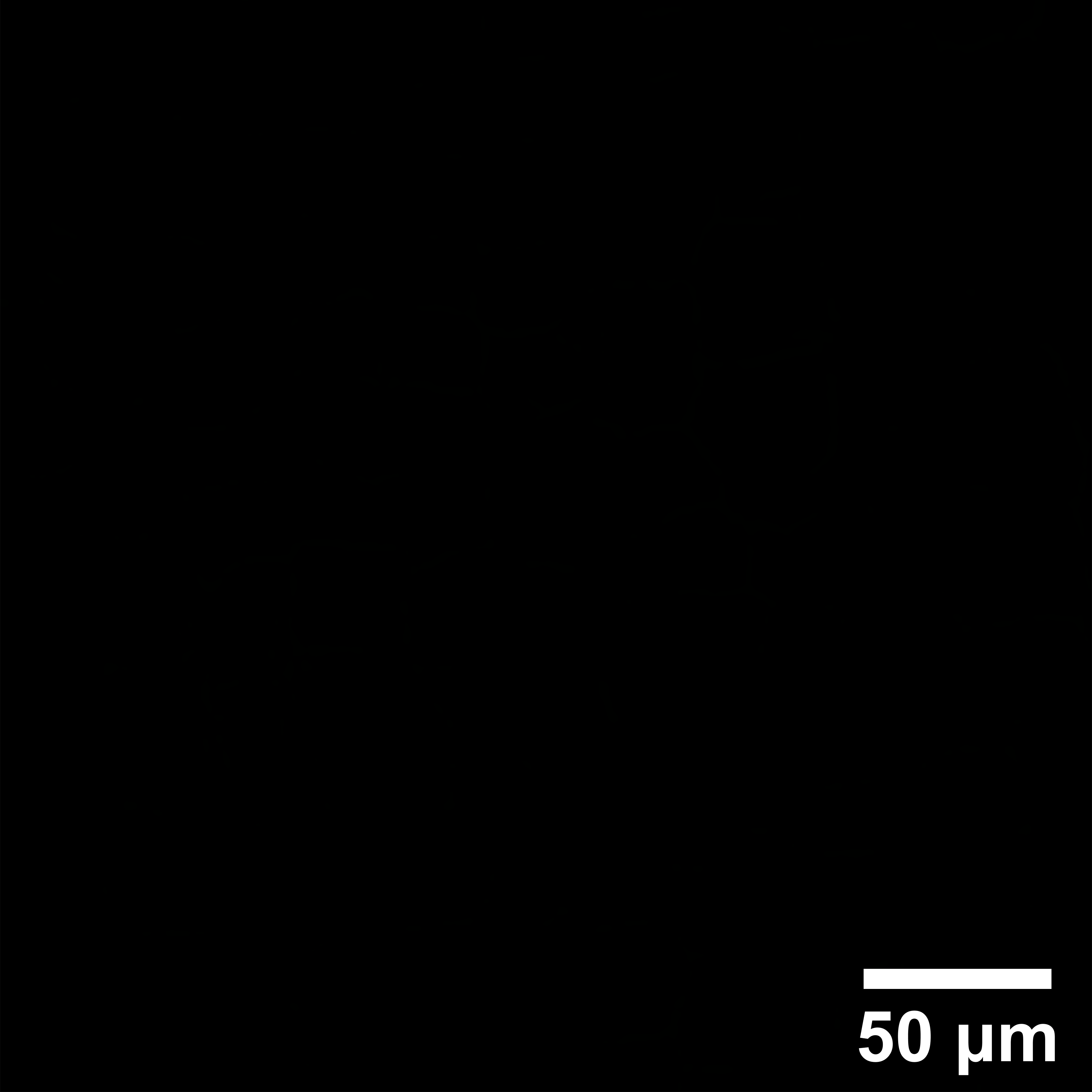

Supplement: Supplementary file 4 — Source data Fig. 2 [file 44319_2026_819_MOESM4_ESM.zip › Figure 2 Source Date/2B/CAT3-Region1.tif]

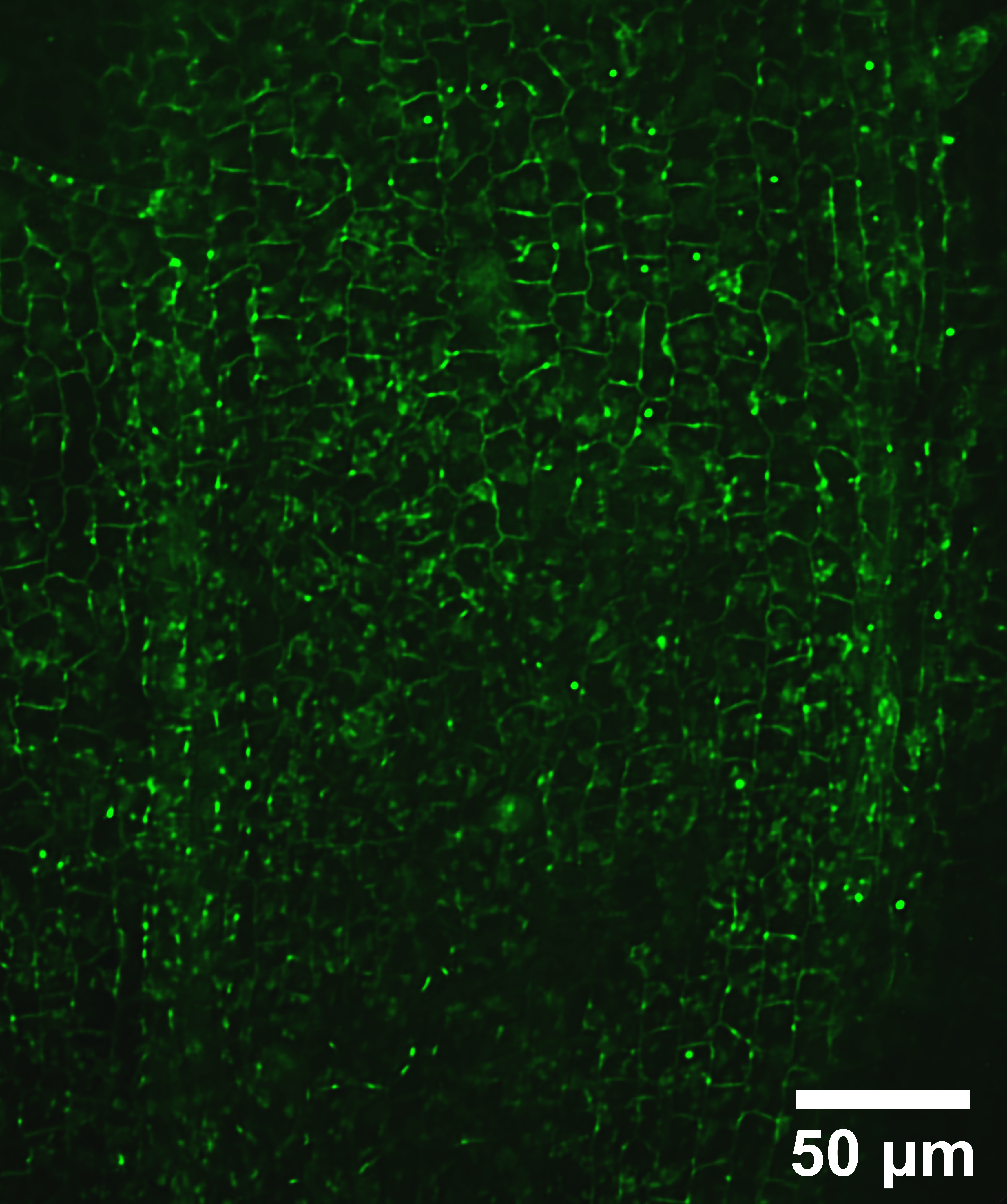

Supplement: Supplementary file 4 — Source data Fig. 2 [file 44319_2026_819_MOESM4_ESM.zip › Figure 2 Source Date/2B/CK1-Region1.tif]

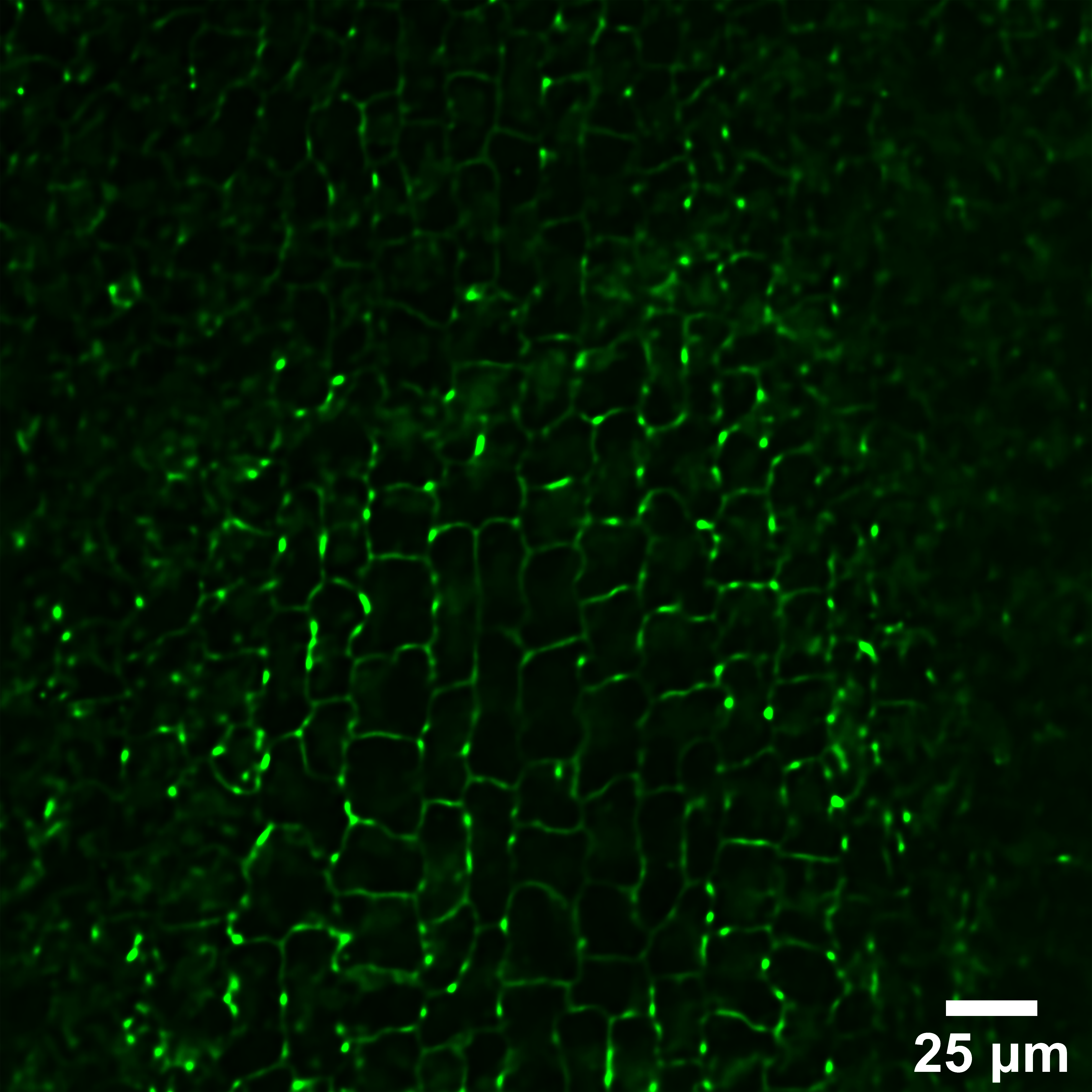

Supplement: Supplementary file 4 — Source data Fig. 2 [file 44319_2026_819_MOESM4_ESM.zip › Figure 2 Source Date/2B/GAI-Region1.tif]

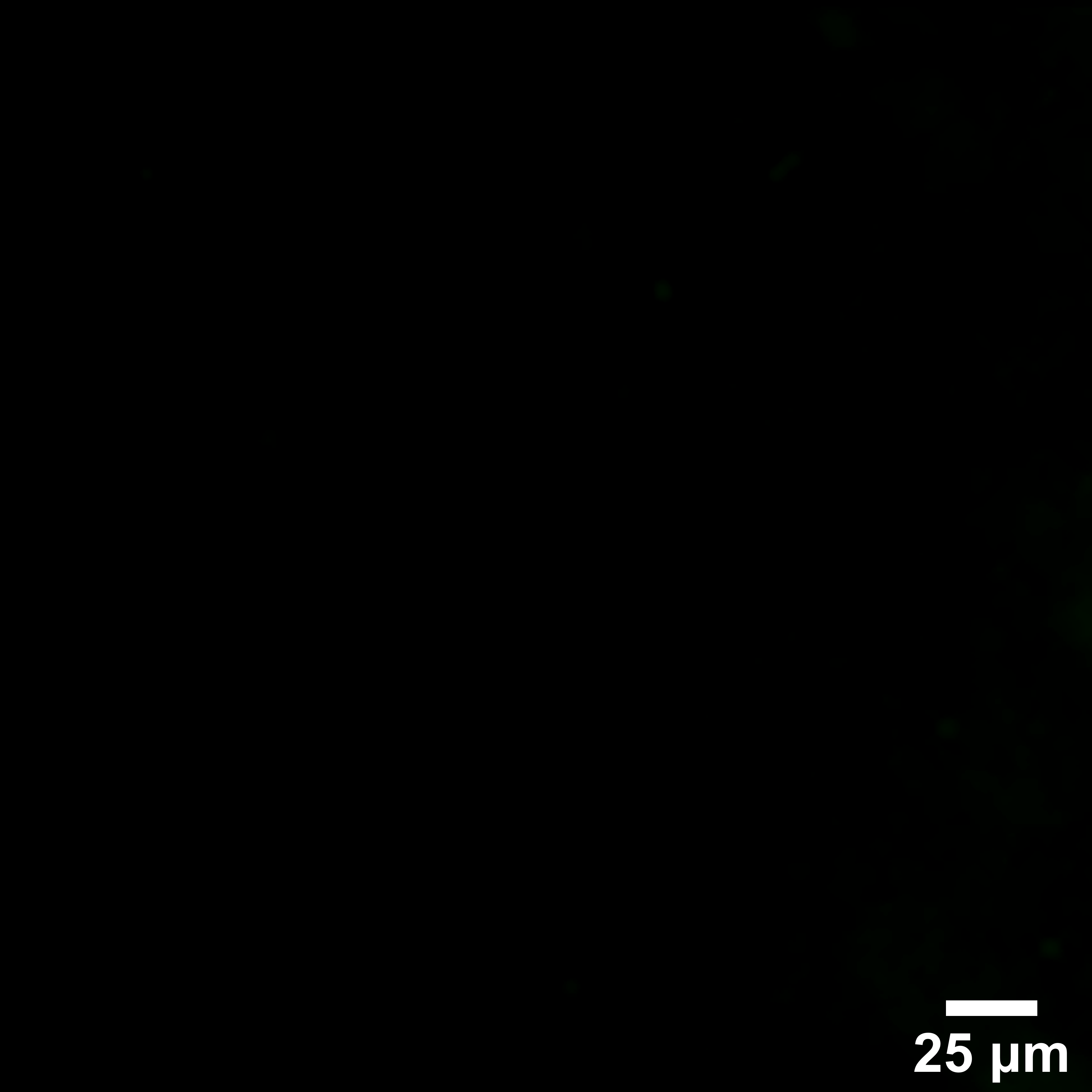

Supplement: Supplementary file 4 — Source data Fig. 2 [file 44319_2026_819_MOESM4_ESM.zip › Figure 2 Source Date/2C/CAT3-tip.tif]

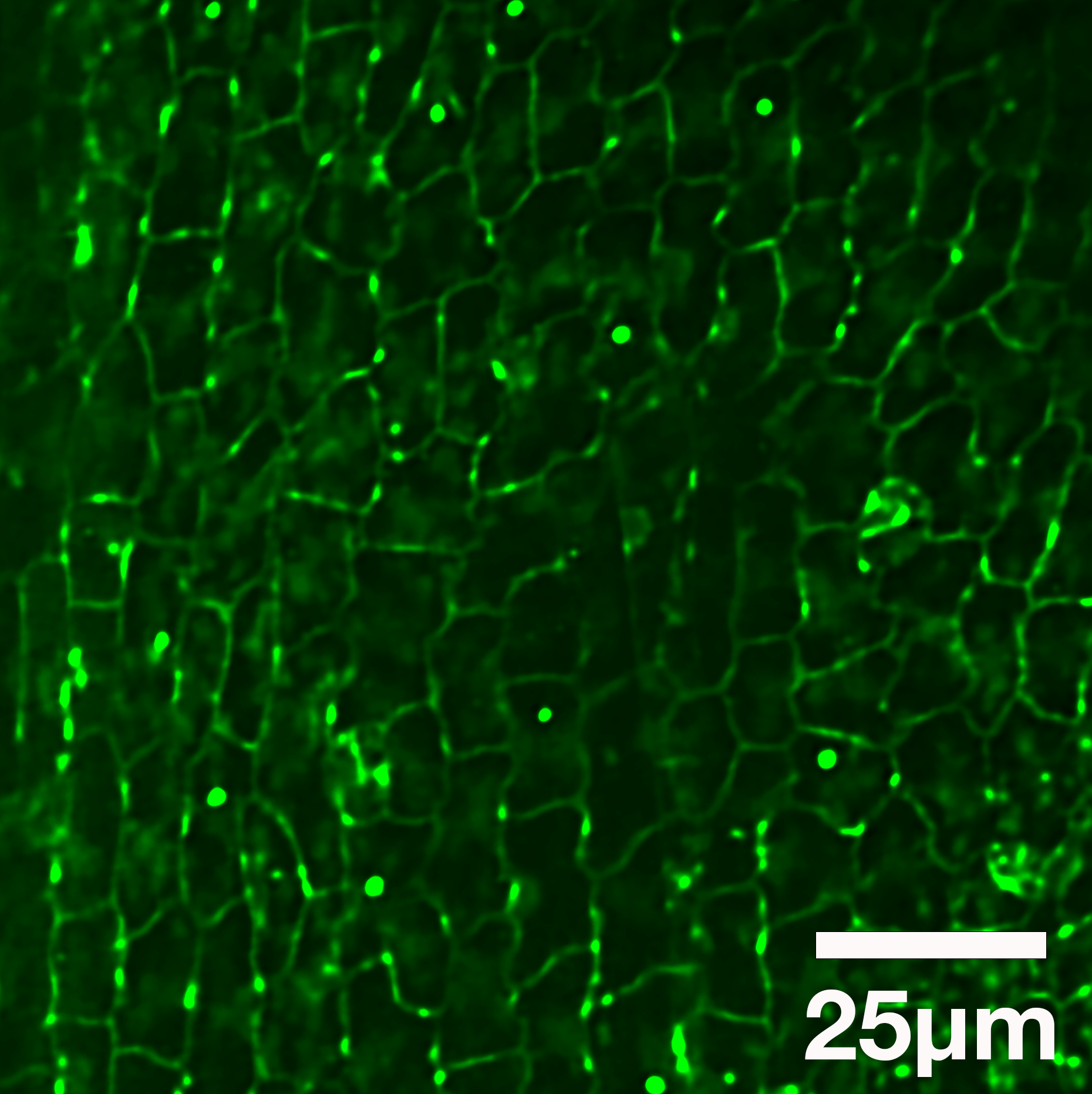

Supplement: Supplementary file 4 — Source data Fig. 2 [file 44319_2026_819_MOESM4_ESM.zip › Figure 2 Source Date/2C/CK-tip.tif.png]

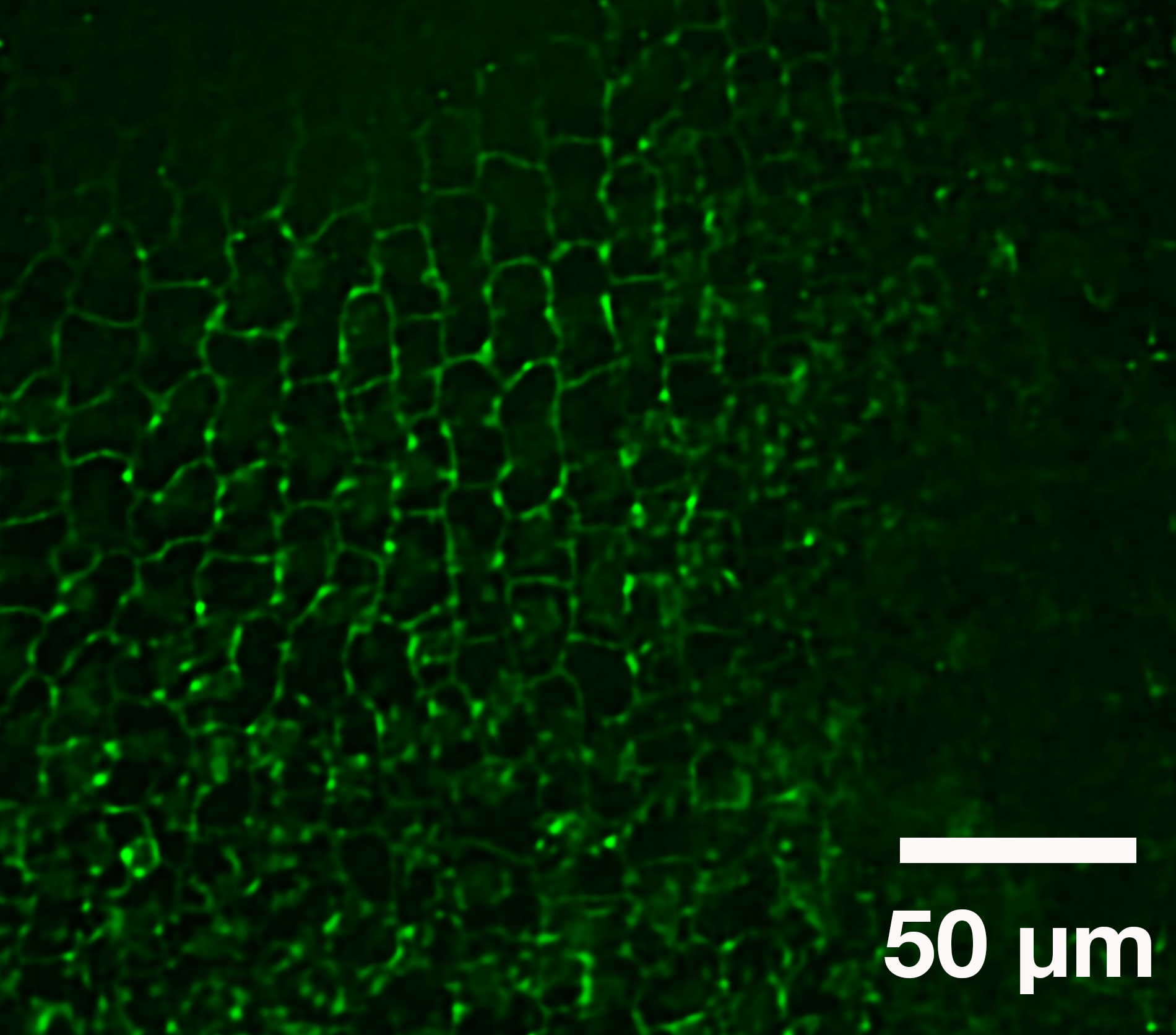

Supplement: Supplementary file 4 — Source data Fig. 2 [file 44319_2026_819_MOESM4_ESM.zip › Figure 2 Source Date/2C/GAI-tip.png]

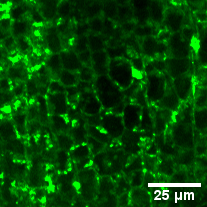

Supplement: Supplementary file 9 — Source data Fig. 4 part 2 [file 44319_2026_819_MOESM9_ESM.zip › Source data Fig. 4 part 2/4D/GME1-Stem tip.tif]

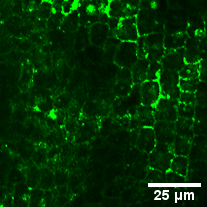

Supplement: Supplementary file 9 — Source data Fig. 4 part 2 [file 44319_2026_819_MOESM9_ESM.zip › Source data Fig. 4 part 2/4D/GME2-Stem tip.tif]

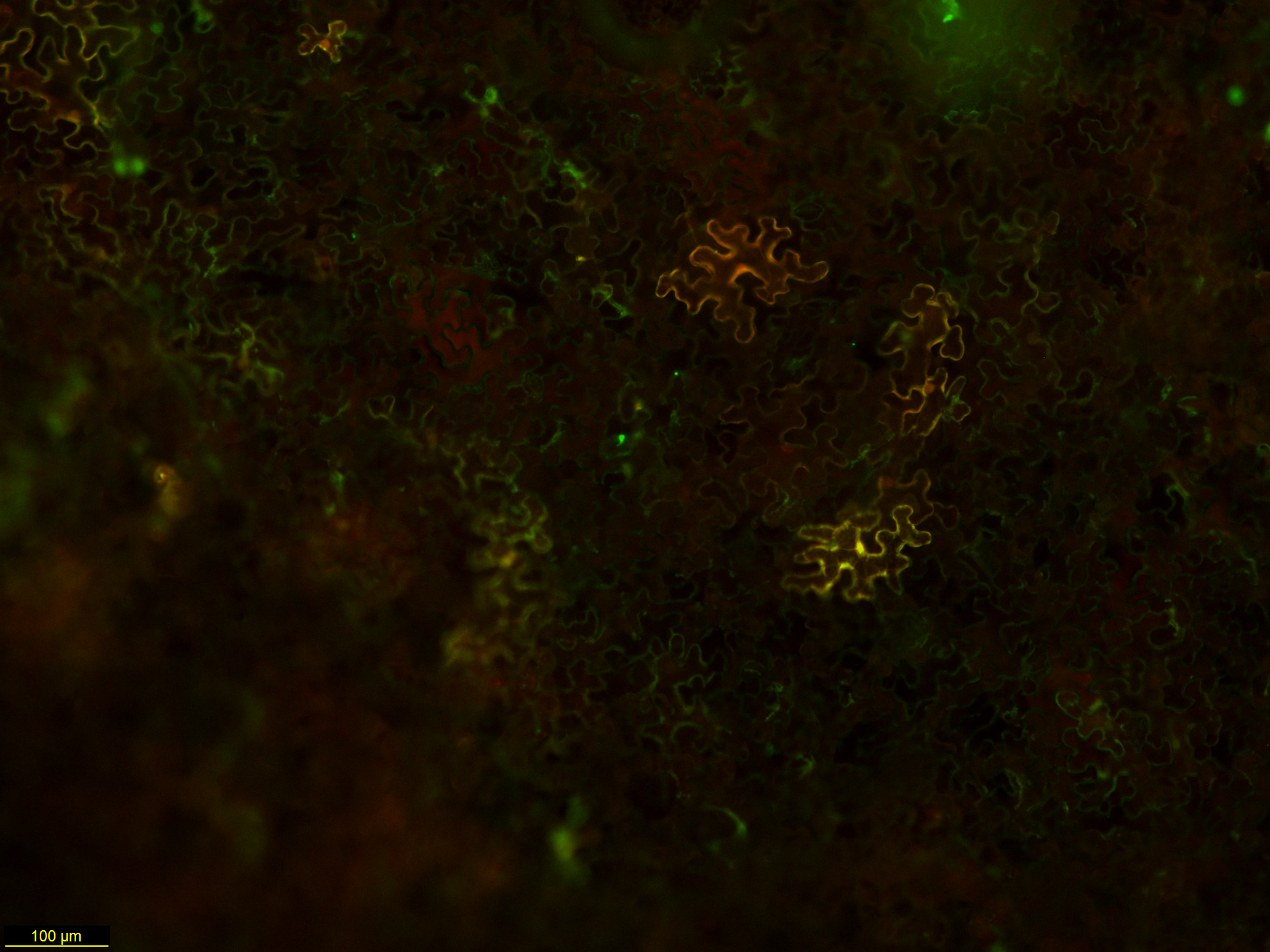

Supplement: Supplementary file 10 — Source data Fig. 5 part 1 [file 44319_2026_819_MOESM10_ESM.zip › Source data Fig. 5 part 1/5C/Region1/GME2-Target-GME2.tif]

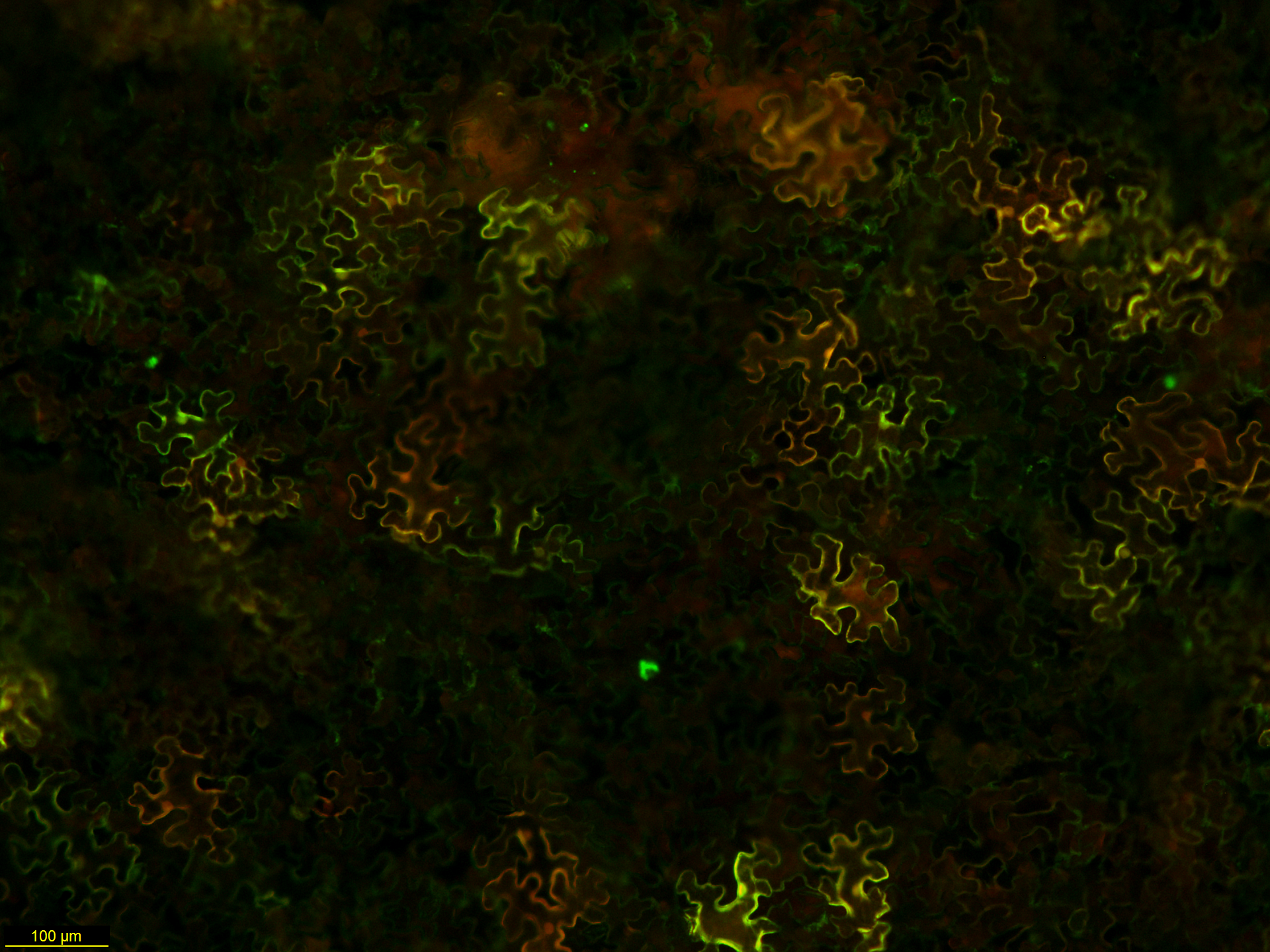

Supplement: Supplementary file 10 — Source data Fig. 5 part 1 [file 44319_2026_819_MOESM10_ESM.zip › Source data Fig. 5 part 1/5C/Region1/Targe-GME2.tif]

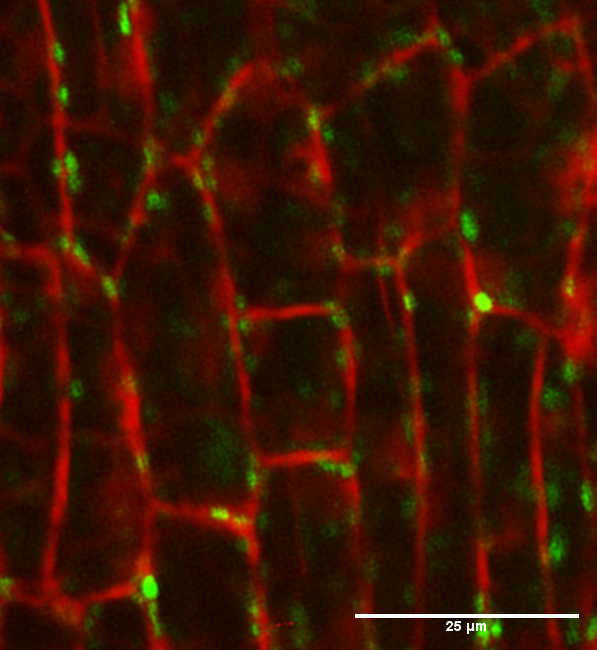

Supplement: Supplementary file 10 — Source data Fig. 5 part 1 [file 44319_2026_819_MOESM10_ESM.zip › Source data Fig. 5 part 1/5C/Region2/2xGME2-Target.tif]

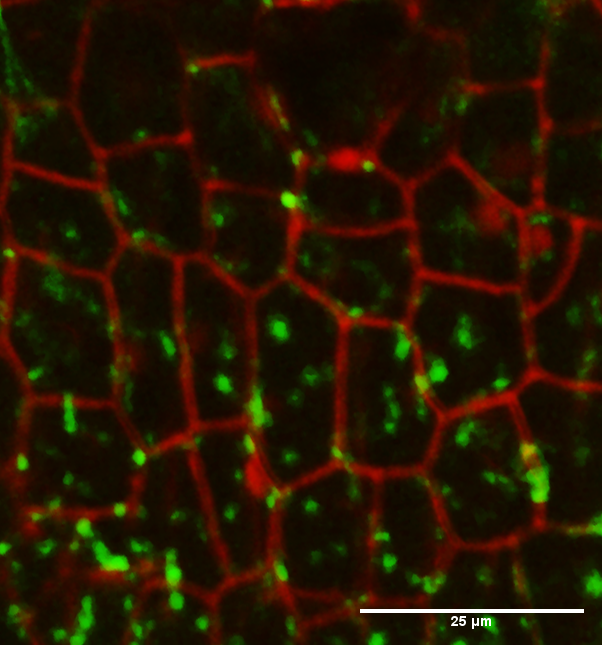

Supplement: Supplementary file 10 — Source data Fig. 5 part 1 [file 44319_2026_819_MOESM10_ESM.zip › Source data Fig. 5 part 1/5C/Region2/GME2-Target-GME2.tif]

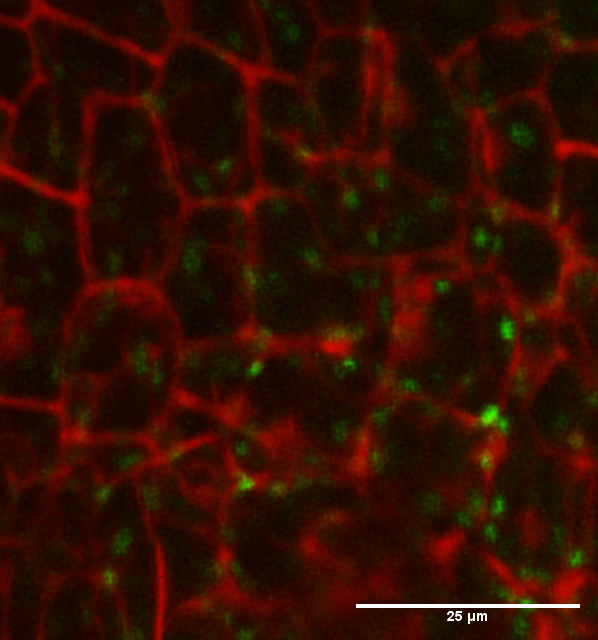

Supplement: Supplementary file 10 — Source data Fig. 5 part 1 [file 44319_2026_819_MOESM10_ESM.zip › Source data Fig. 5 part 1/5C/Region2/GME2-Target.tif]

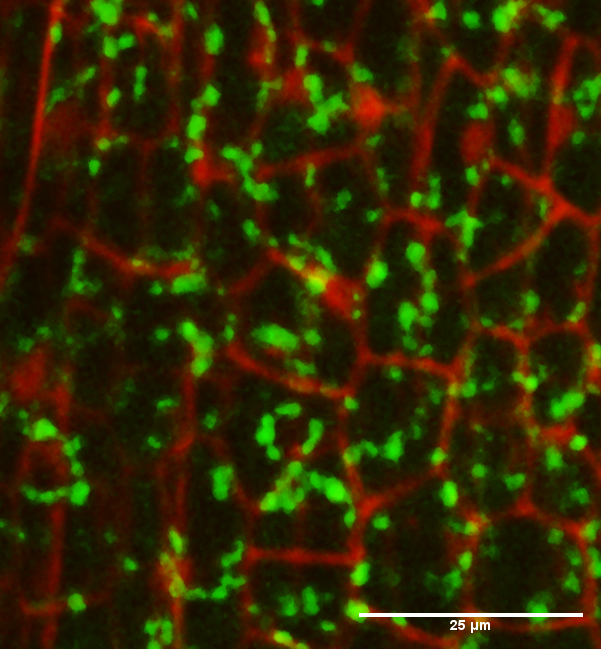

Supplement: Supplementary file 10 — Source data Fig. 5 part 1 [file 44319_2026_819_MOESM10_ESM.zip › Source data Fig. 5 part 1/5C/Region2/Target-2GME2.tif]

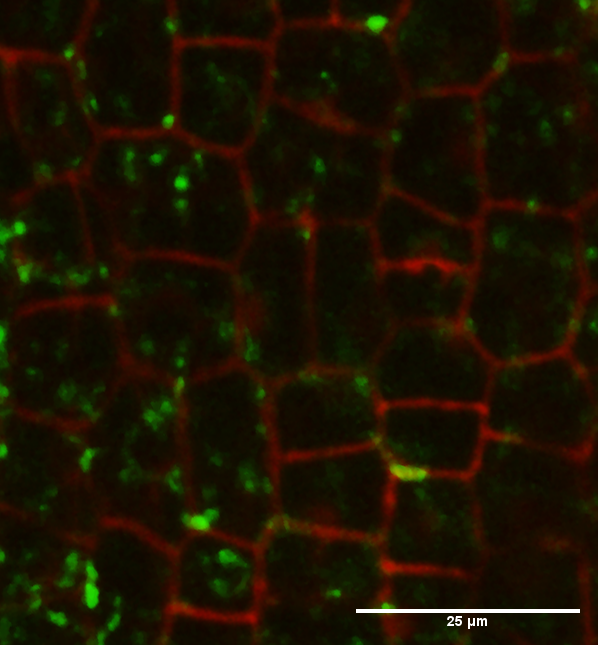

Supplement: Supplementary file 10 — Source data Fig. 5 part 1 [file 44319_2026_819_MOESM10_ESM.zip › Source data Fig. 5 part 1/5C/Region2/Target-GME2.tif]

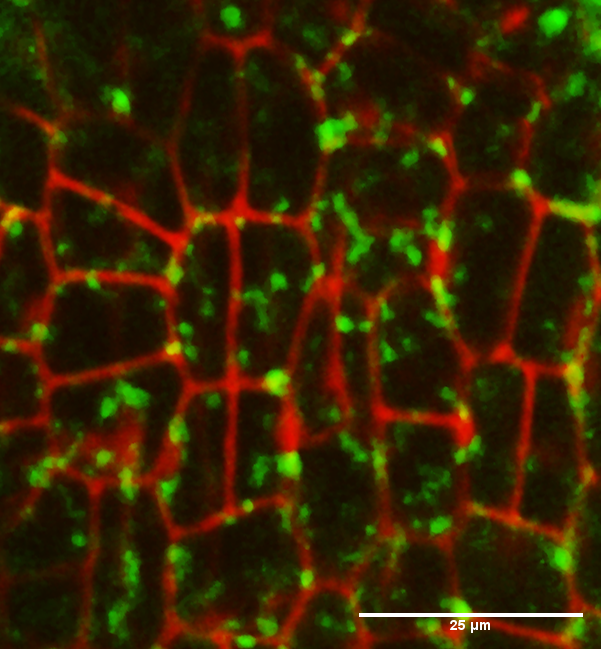

Supplement: Supplementary file 11 — Source data Fig. 5 part 2 [file 44319_2026_819_MOESM11_ESM.zip › Source data Fig. 5 part 2/5D/1xMC-3WJ-4xBro-2xGME2.tif]

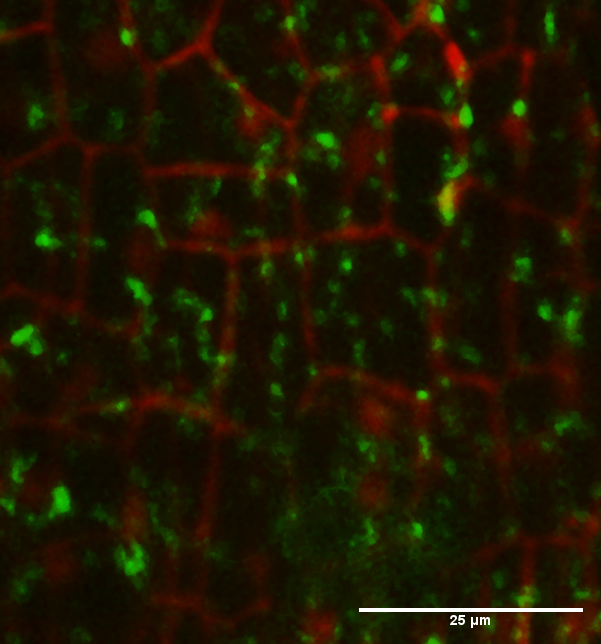

Supplement: Supplementary file 11 — Source data Fig. 5 part 2 [file 44319_2026_819_MOESM11_ESM.zip › Source data Fig. 5 part 2/5D/2xMC-3WJ-4xBro-2xGME2.tif]

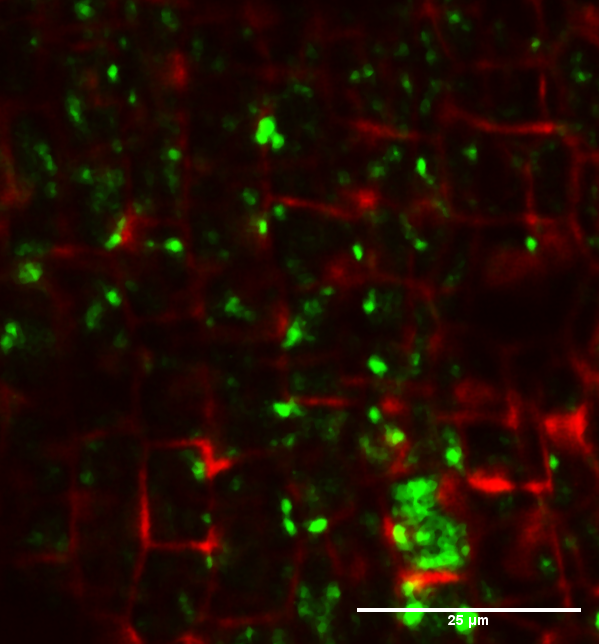

Supplement: Supplementary file 11 — Source data Fig. 5 part 2 [file 44319_2026_819_MOESM11_ESM.zip › Source data Fig. 5 part 2/5D/3xMC-3WJ-4xBro-2xGME2.tif]

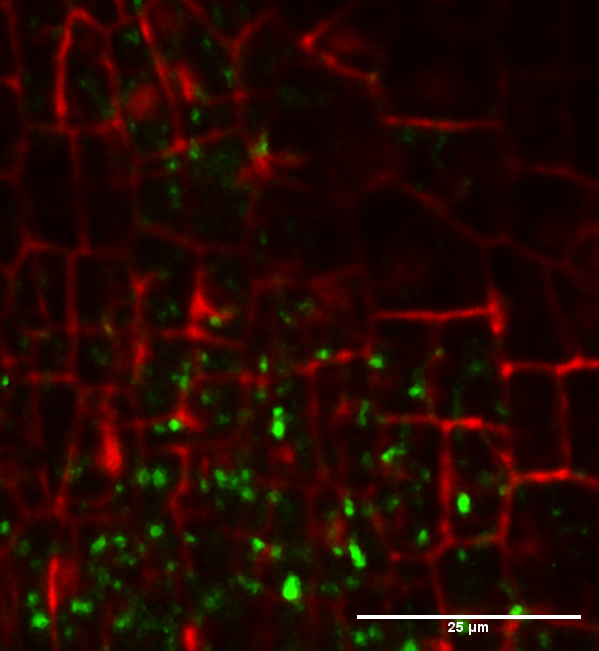

Supplement: Supplementary file 11 — Source data Fig. 5 part 2 [file 44319_2026_819_MOESM11_ESM.zip › Source data Fig. 5 part 2/5D/4xMC-3WJ-4xBro-2xGME2.tif]

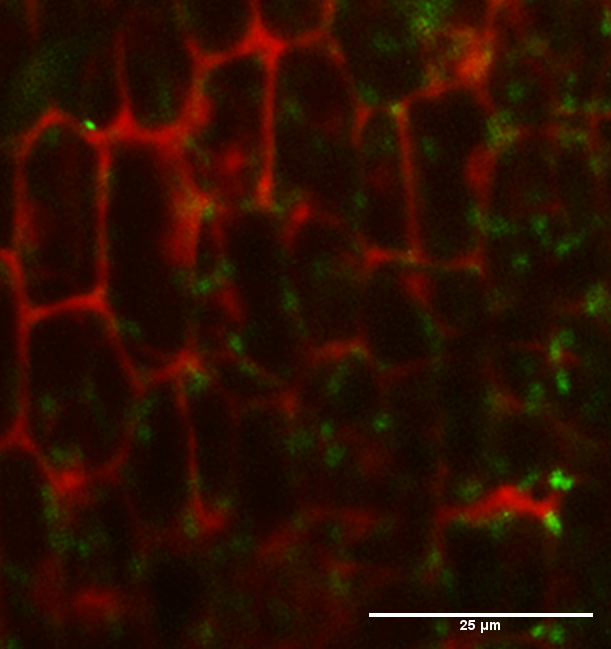

Supplement: Supplementary file 11 — Source data Fig. 5 part 2 [file 44319_2026_819_MOESM11_ESM.zip › Source data Fig. 5 part 2/5D/5xMC-3WJ-4xBro-2xGME2.tif]

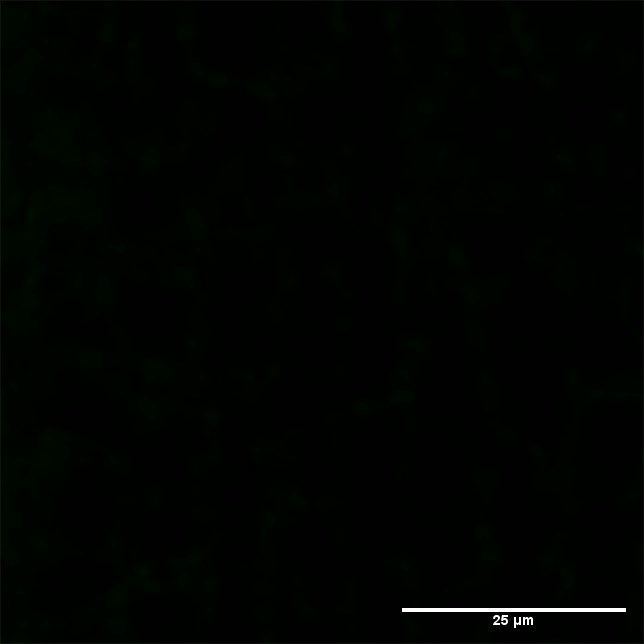

Supplement: Supplementary file 11 — Source data Fig. 5 part 2 [file 44319_2026_819_MOESM11_ESM.zip › Source data Fig. 5 part 2/5D/6xMC-3WJ-4xBro-2xGME2.tif]

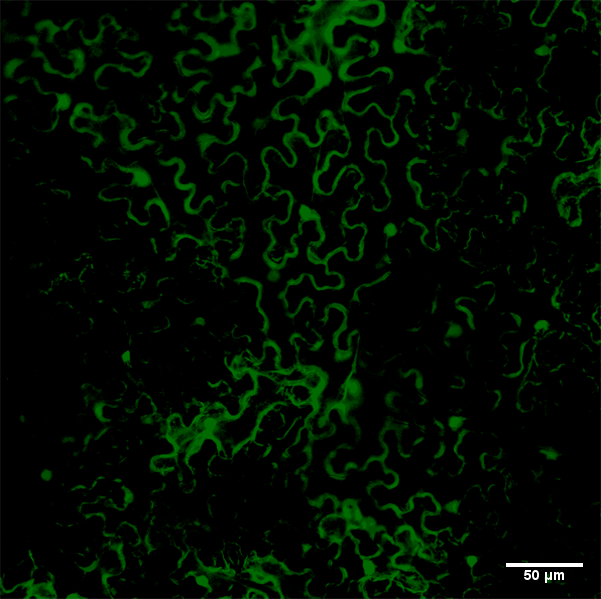

Supplement: Supplementary file 12 — Source data Fig. 6 [file 44319_2026_819_MOESM12_ESM.zip › FIG6/6A/Scion-AtMYB49-3WJ-4×Bro-2×GME2.tif]

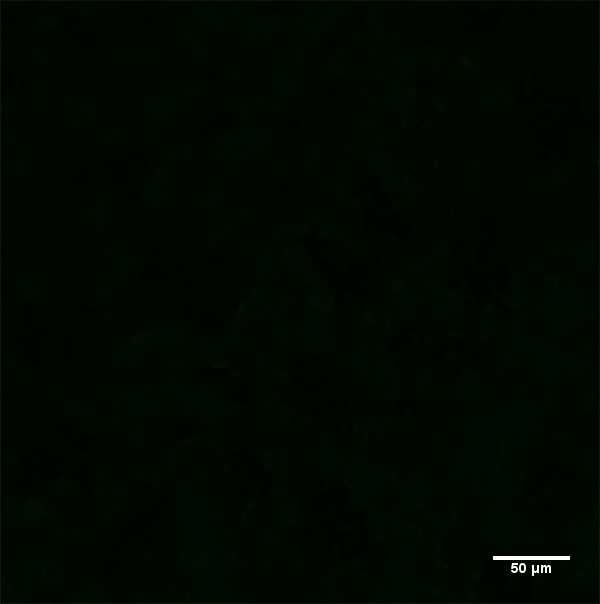

Supplement: Supplementary file 12 — Source data Fig. 6 [file 44319_2026_819_MOESM12_ESM.zip › FIG6/6A/Scion-AtMYB49-3WJ-4×Bro.tif]

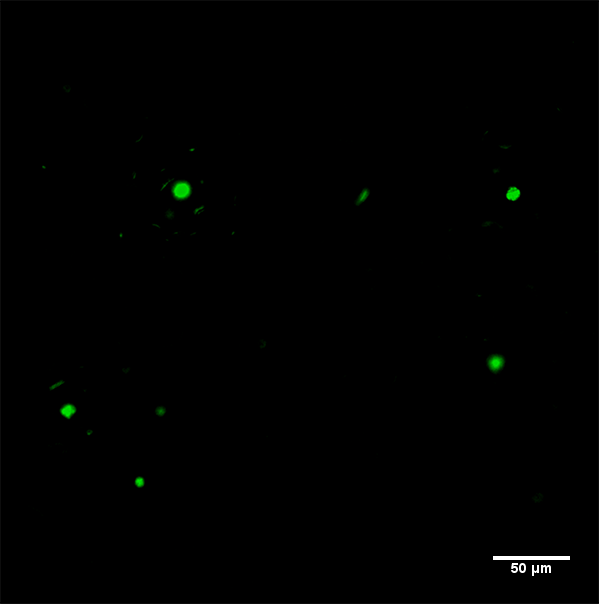

Supplement: Supplementary file 12 — Source data Fig. 6 [file 44319_2026_819_MOESM12_ESM.zip › FIG6/6A/Scion-AtMYB49-GFP-2×GME2.tif]

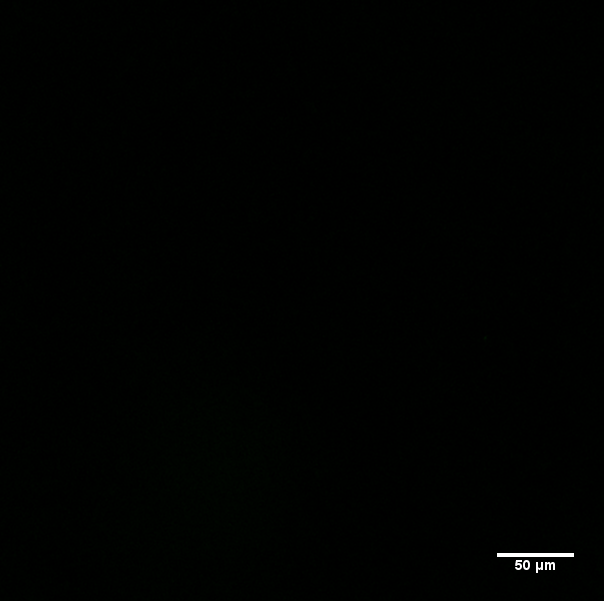

Supplement: Supplementary file 12 — Source data Fig. 6 [file 44319_2026_819_MOESM12_ESM.zip › FIG6/6A/Scion-AtMYB49-GFP.tif]

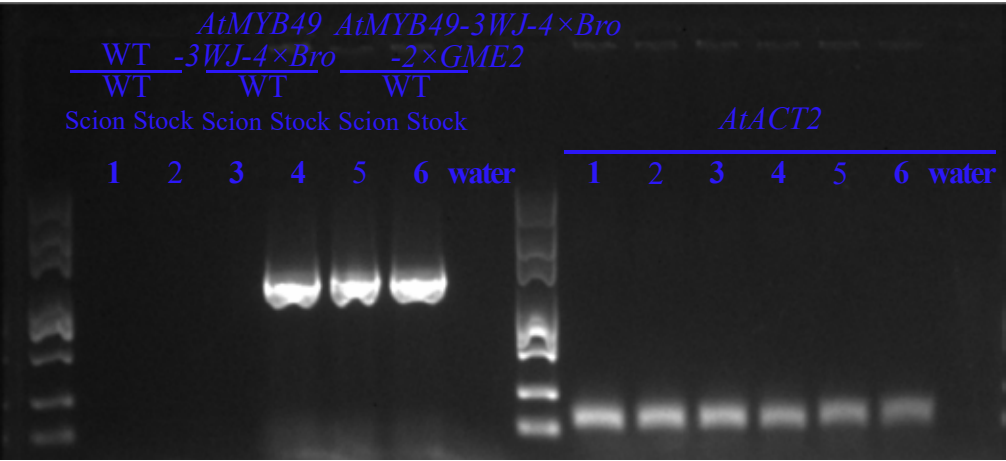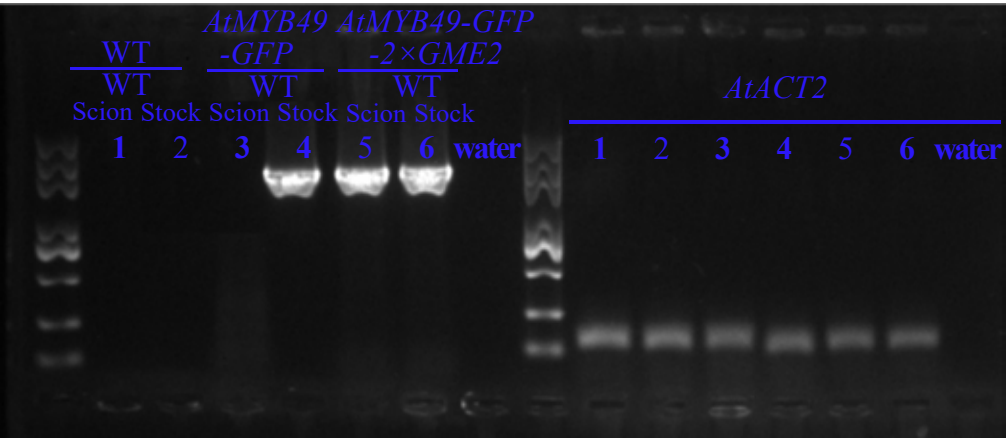

Supplement: Supplementary file 12 — Source data Fig. 6 [file 44319_2026_819_MOESM12_ESM.zip › FIG6/6B/6B.pdf]

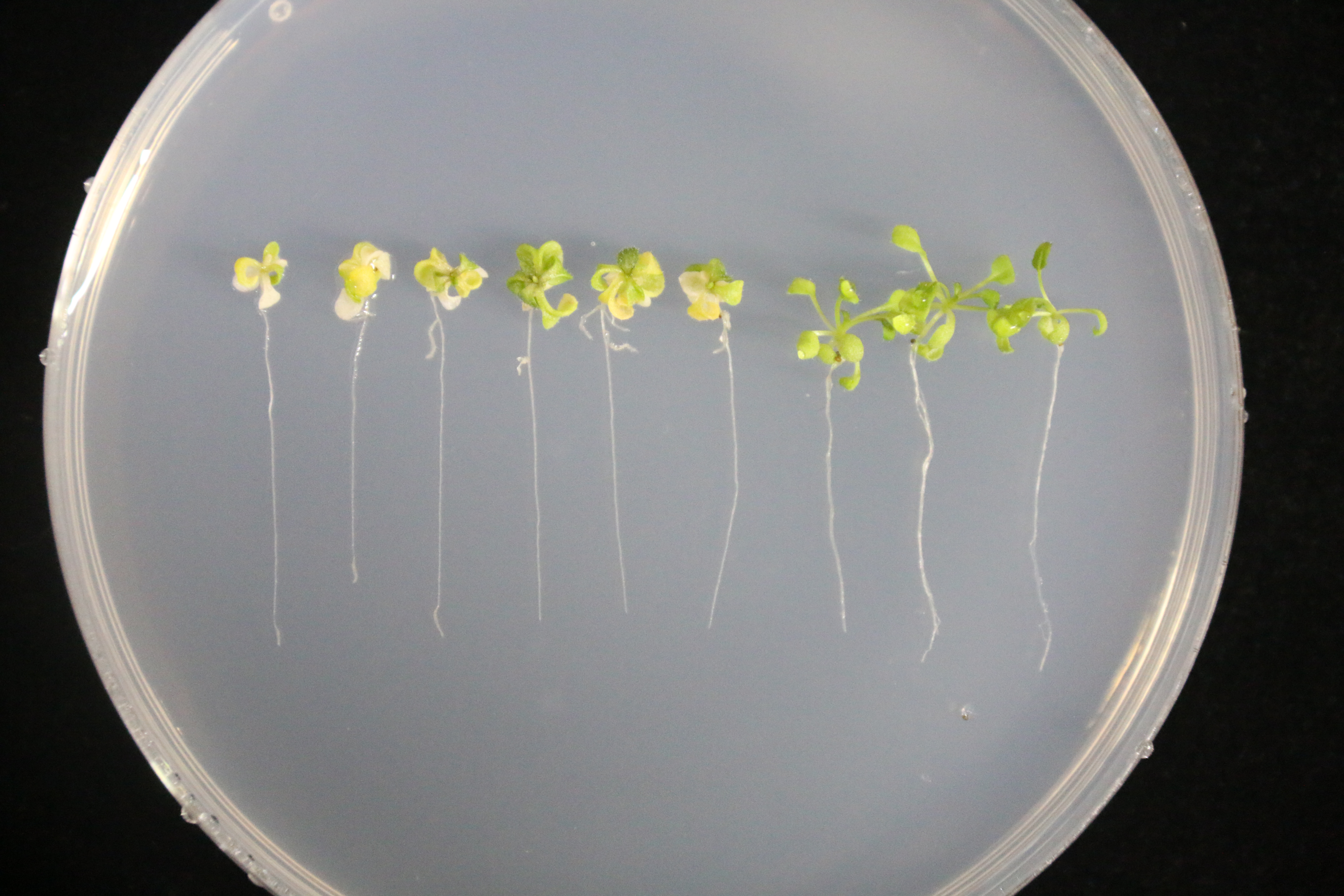

Supplement: Supplementary file 12 — Source data Fig. 6 [file 44319_2026_819_MOESM12_ESM.zip › FIG6/6C/IMG_5359.JPG]

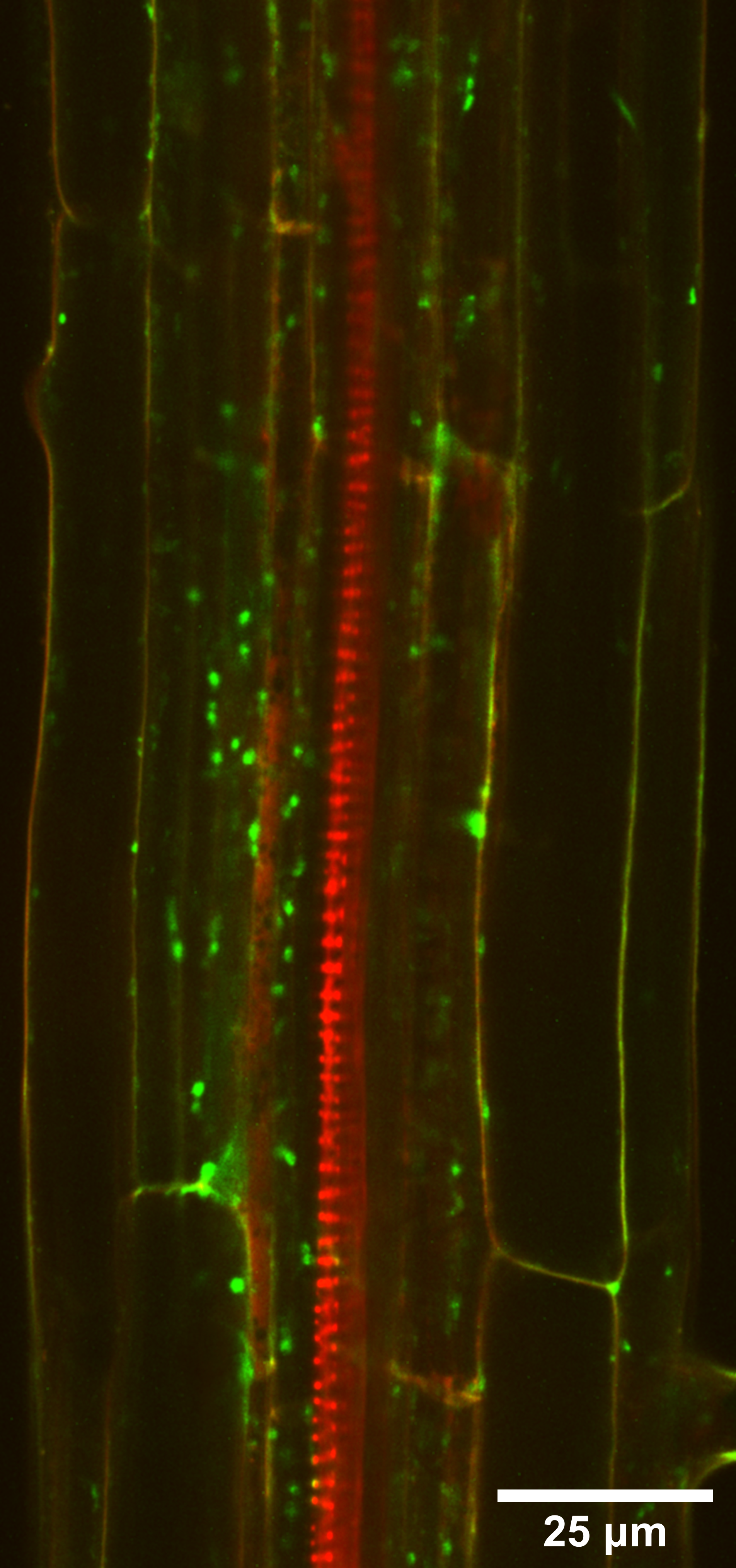

Supplement: Supplementary file 12 — Source data Fig. 6 [file 44319_2026_819_MOESM12_ESM.zip › FIG6/6E/Elongation-AtHMA3-3WJ-4×Bro-2× GME2.tif]

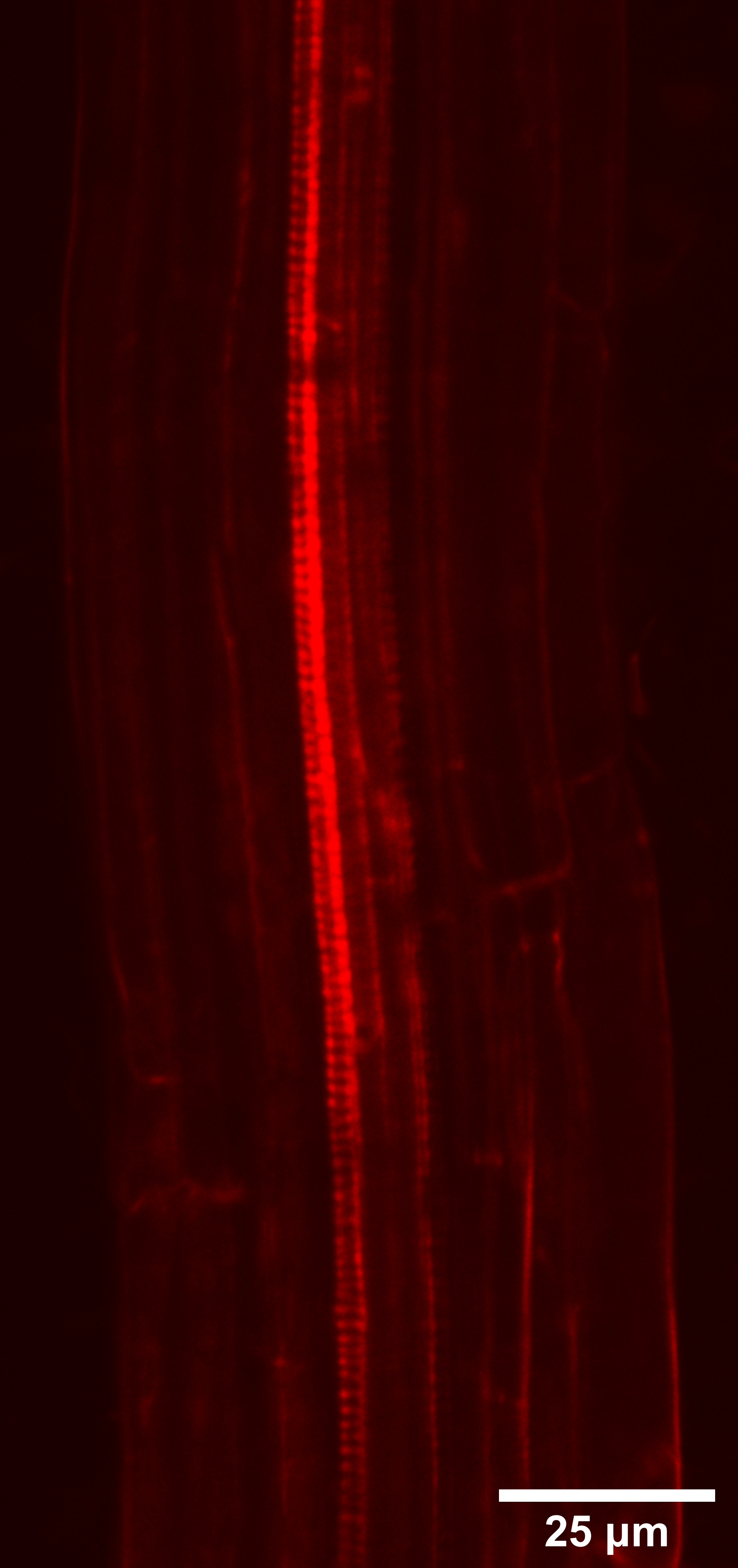

Supplement: Supplementary file 12 — Source data Fig. 6 [file 44319_2026_819_MOESM12_ESM.zip › FIG6/6E/Elongation-AtHMA3-3WJ-4×Bro.tif]

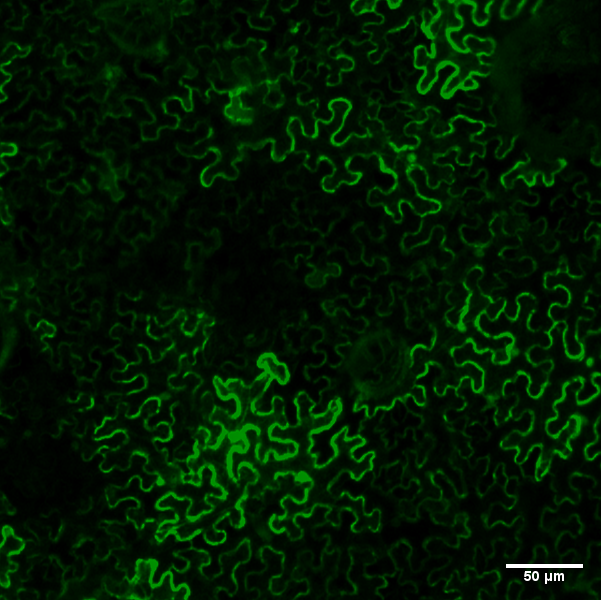

Supplement: Supplementary file 12 — Source data Fig. 6 [file 44319_2026_819_MOESM12_ESM.zip › FIG6/6E/Leaf-AtHMA3-3WJ-4×Bro-2× GME2.tif]

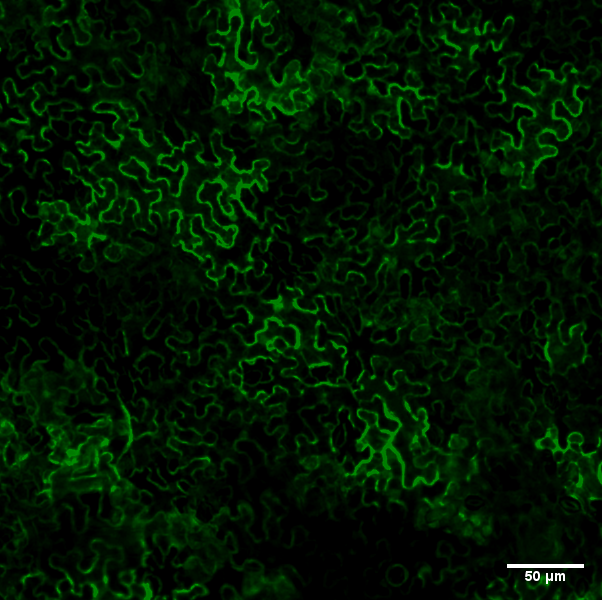

Supplement: Supplementary file 12 — Source data Fig. 6 [file 44319_2026_819_MOESM12_ESM.zip › FIG6/6E/Leaf-AtHMA3-3WJ-4×Bro.tif]

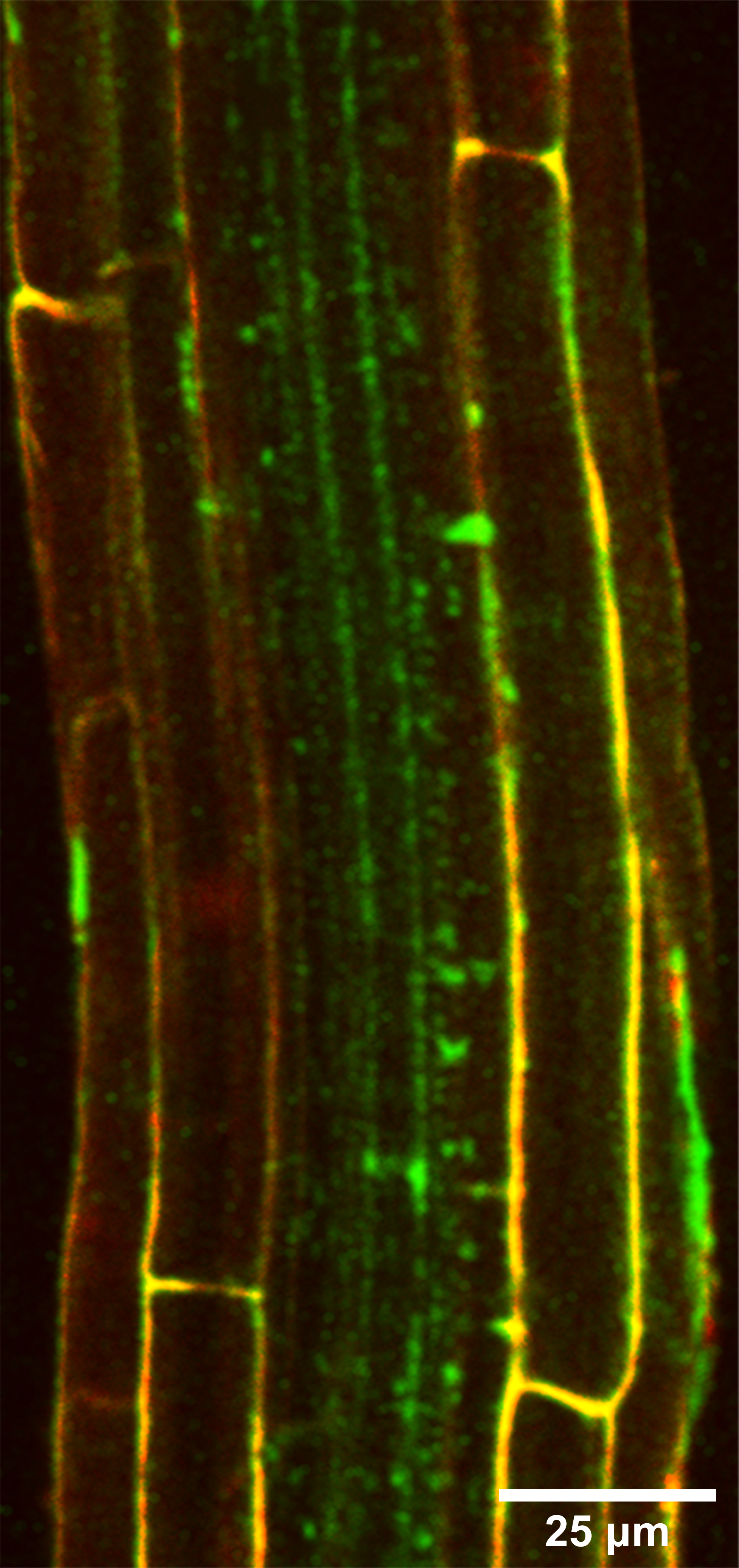

Supplement: Supplementary file 12 — Source data Fig. 6 [file 44319_2026_819_MOESM12_ESM.zip › FIG6/6E/Maturation-AtHMA3-3WJ-4×Bro-2× GME2.tif]

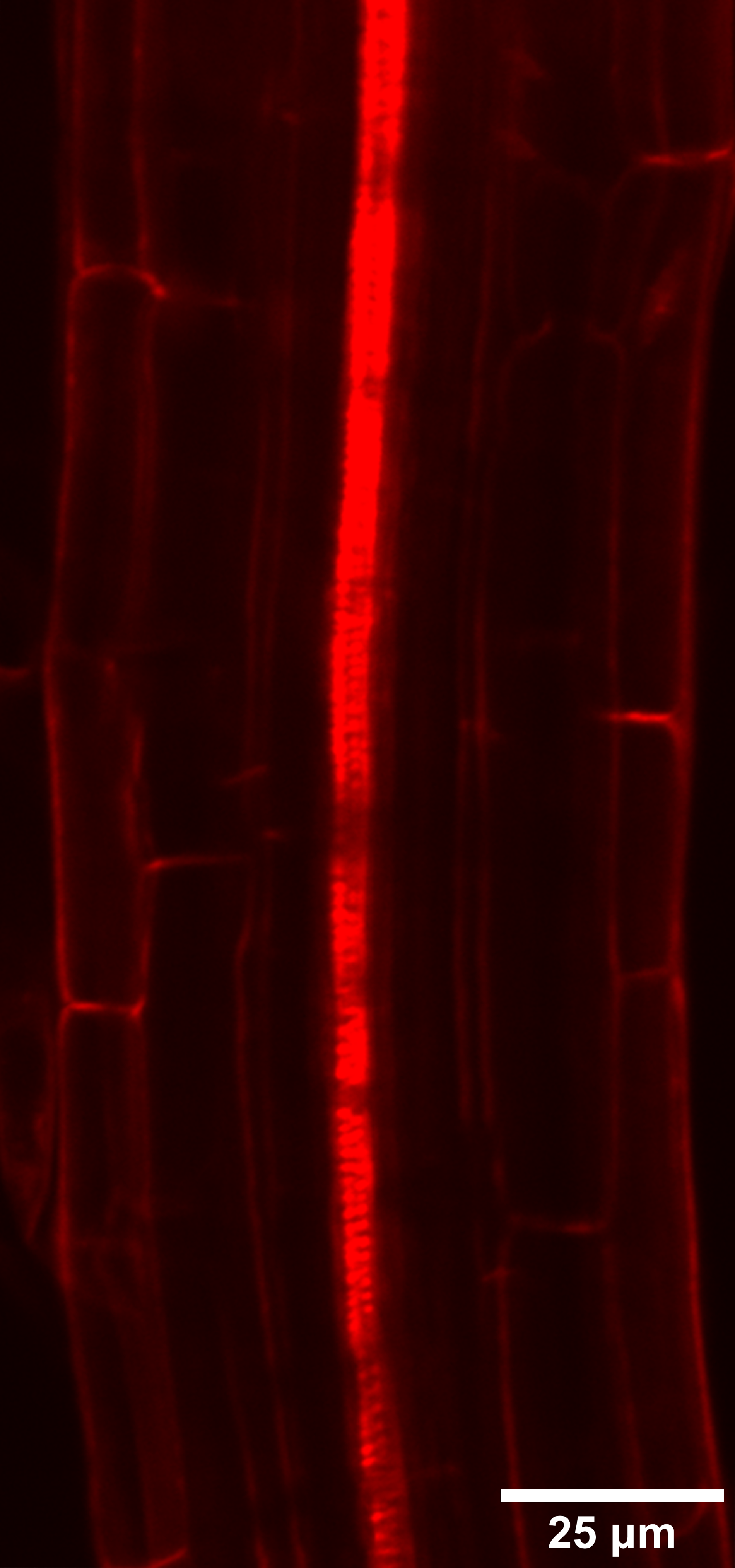

Supplement: Supplementary file 12 — Source data Fig. 6 [file 44319_2026_819_MOESM12_ESM.zip › FIG6/6E/Maturation-AtHMA3-3WJ-4×Bro.tif]

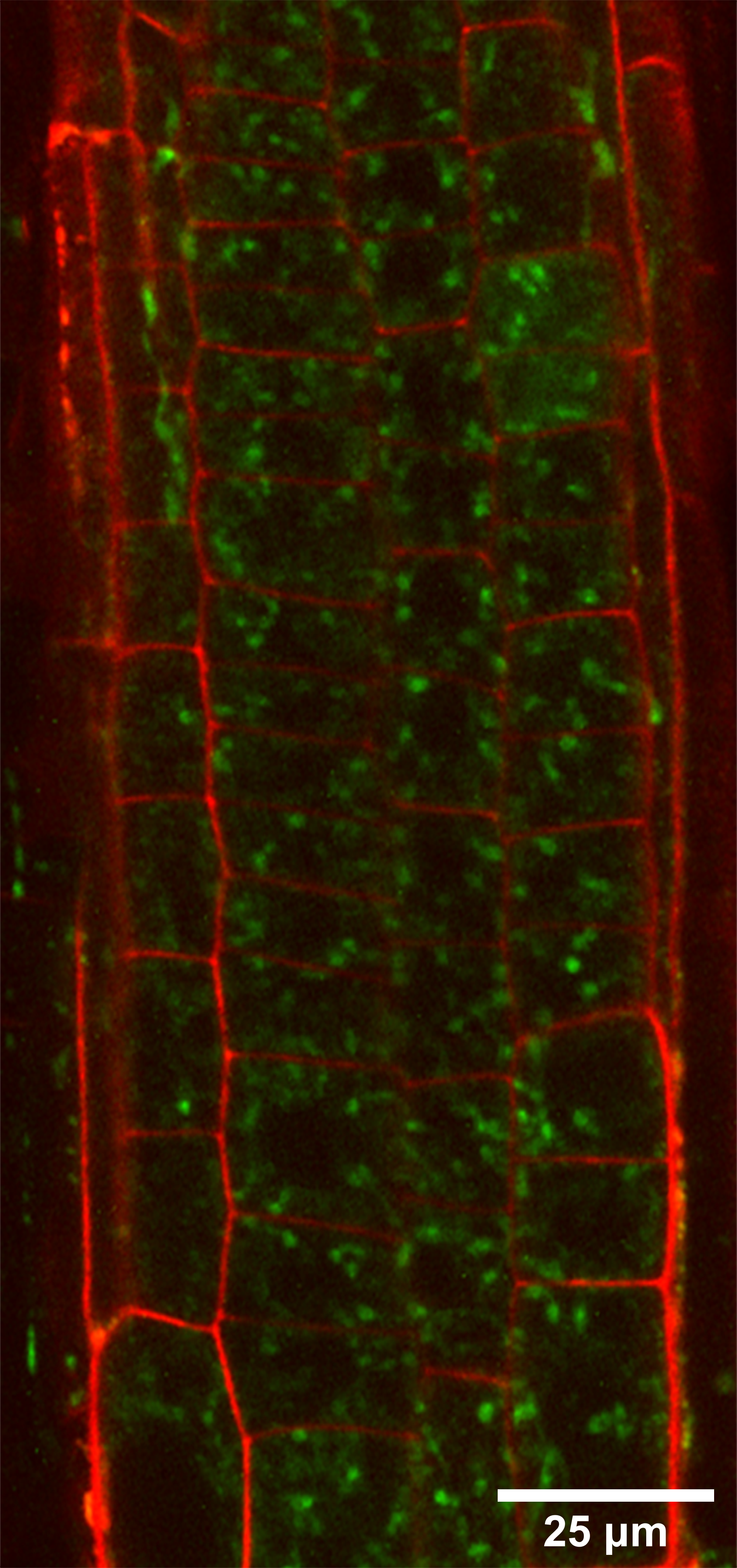

Supplement: Supplementary file 12 — Source data Fig. 6 [file 44319_2026_819_MOESM12_ESM.zip › FIG6/6E/Root-AtHMA3-3WJ-4×Bro-2× GME2.tif]

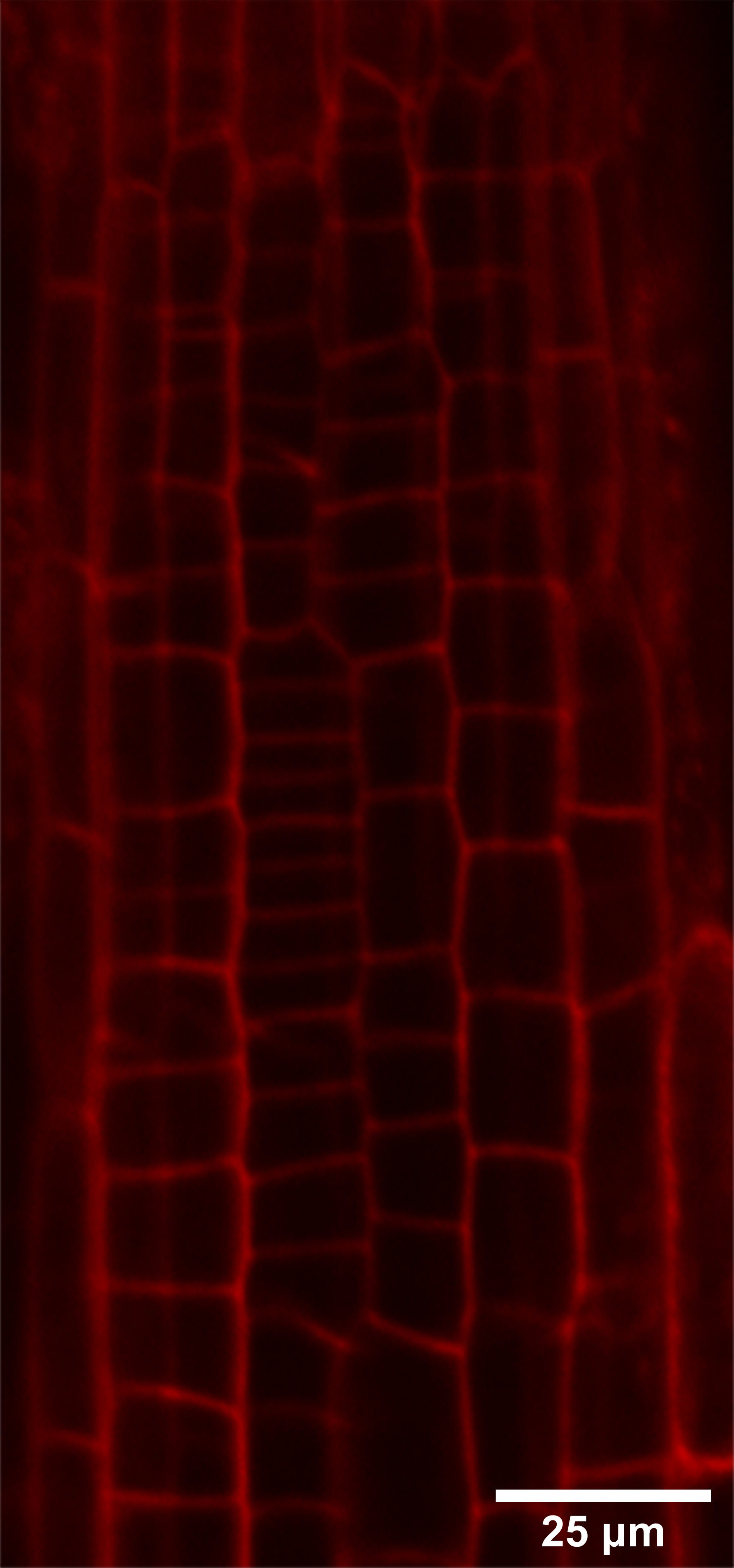

Supplement: Supplementary file 12 — Source data Fig. 6 [file 44319_2026_819_MOESM12_ESM.zip › FIG6/6E/Root-AtHMA3-3WJ-4×Bro.tif]

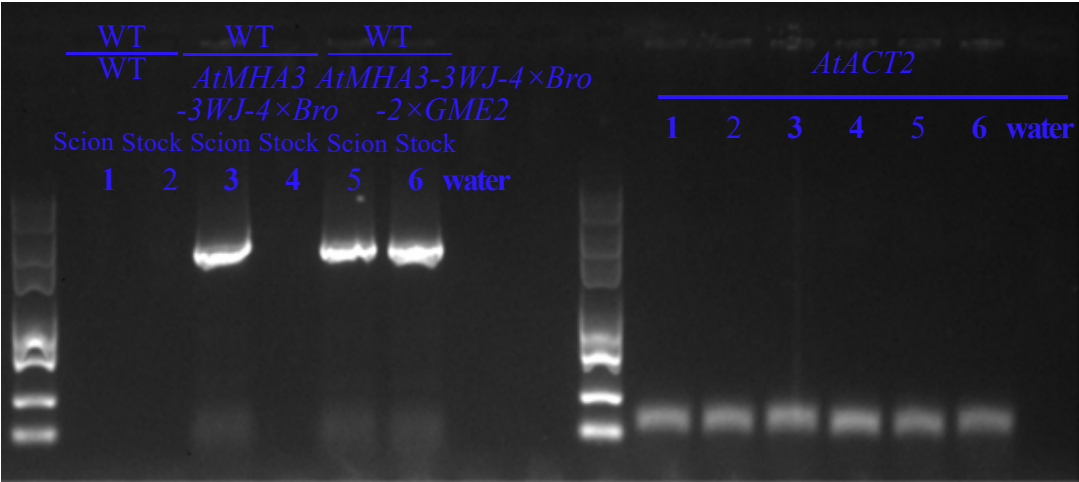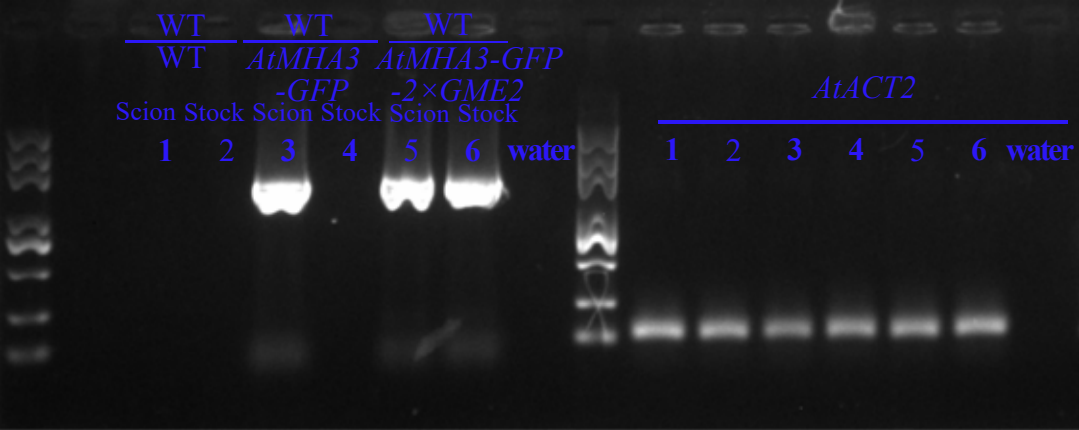

Supplement: Supplementary file 12 — Source data Fig. 6 [file 44319_2026_819_MOESM12_ESM.zip › FIG6/6F/6F.pdf]

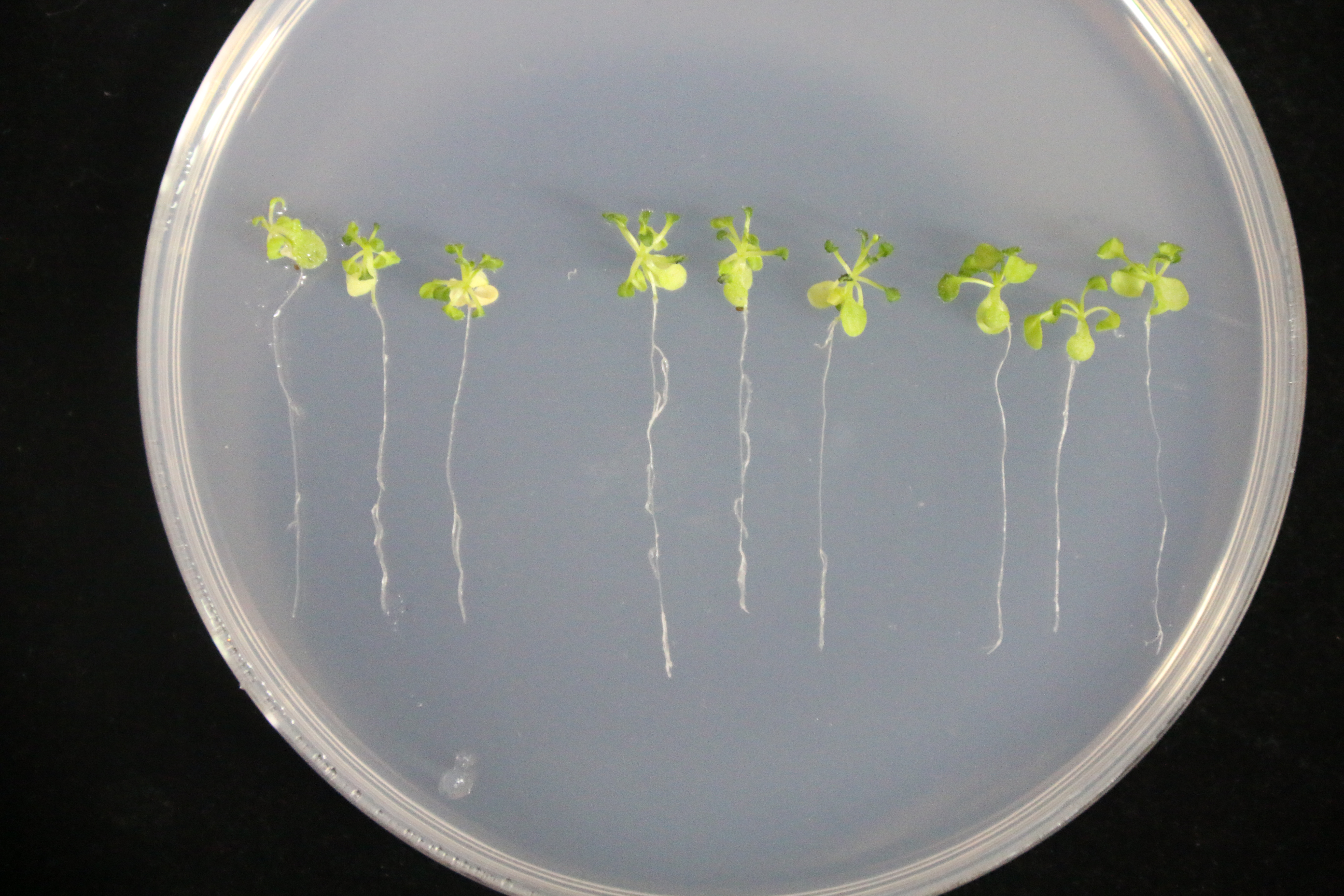

Supplement: Supplementary file 12 — Source data Fig. 6 [file 44319_2026_819_MOESM12_ESM.zip › FIG6/6G/IMG_5356.JPG]
