# Supplementary material for: Developing a mobile RNA delivery system for grafting trait improvement
Source: EMBO Rep. 2026 Jun 10;27(13):3863–89. doi: 10.1038/s44319-026-00819-z (PMC13354801; doi:10.1038/s44319-026-00819-z)
Supplement: Supplementary file 13 — Expanded View Figures [file 44319_2026_819_MOESM13_ESM.pdf]

Expanded View Figures

Figure EV1. 3WJ-4×Bro enables imaging various mobile mRNAs.

(**A**) Sanger sequencing validation of *TCTP1*-3WJ-4×Bro in regions 2 and 3. (**B**) The RT–PCR analysis confirmed the presence of 3WJ-4×Bro-tagged RNA in root phloem. Samples were collected 1.5 cm below to the graft junction. The reference gene *AtACT2* was utilized in the analysis. (**C**) Sanger sequencing validation *TCTP1-3WJ-4×Bro* in wild-type (WT) stocks. (**D**) Representative confocal images of mock-infiltrated *N. benthamiana* leaves transiently expressing 3WJ-4×Bro alone. Samples were collected at 56 h post-infiltration (hpi). Scale bar, 50 μm.

 © The Author(s)

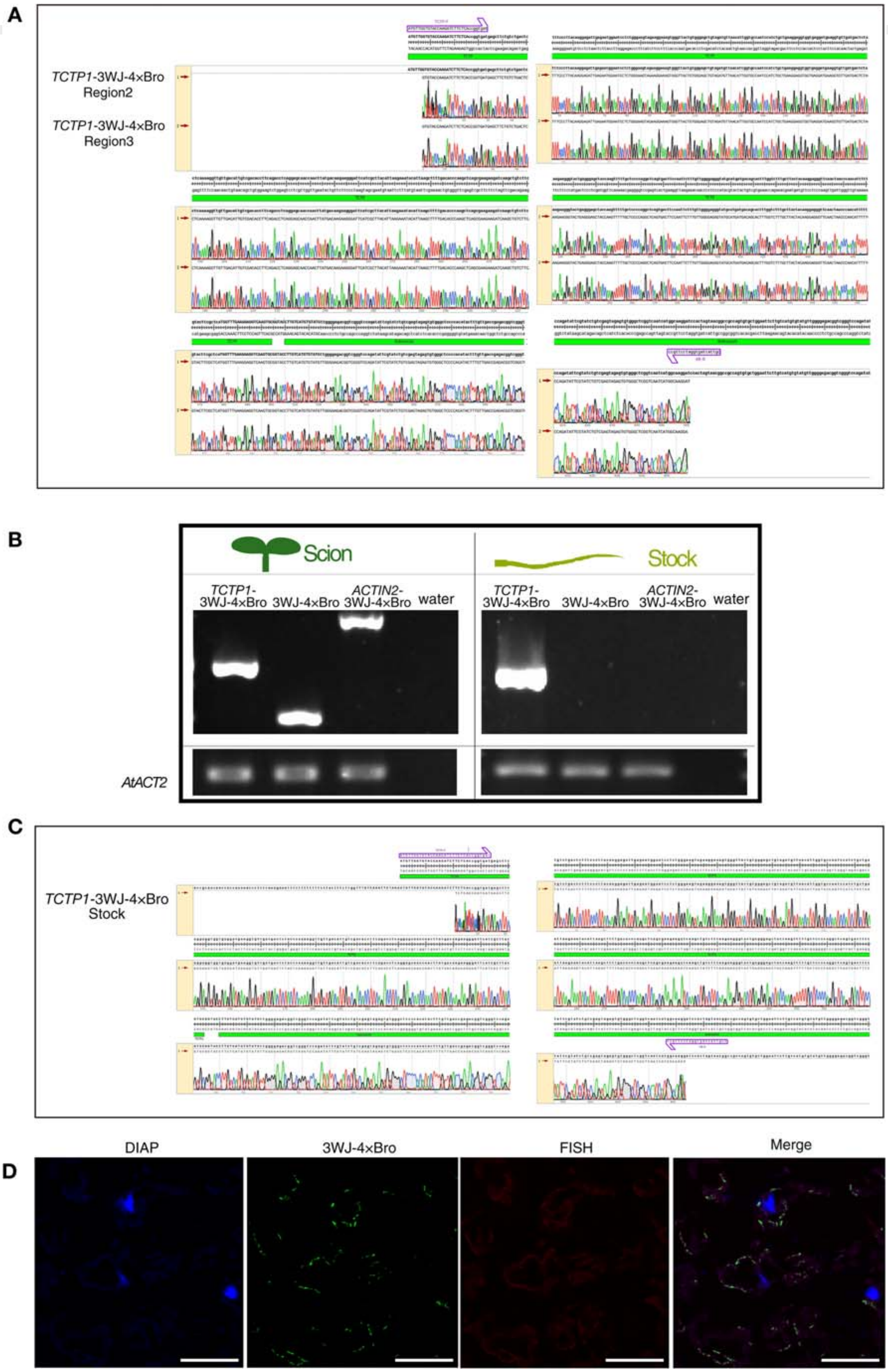
A
TCTP1-3WJ-4×Bro Region2
TCTP1-3WJ-4×Bro Region3
B
Scion
Stock
TCTP1-3WJ-4×Bro
3WJ-4×Bro
ACTIN2-3WJ-4×Bro
water
AtACT2
C
TCTP1-3WJ-4×Bro Stock
DIAP
3WJ-4×Bro
FISH
Merge
D

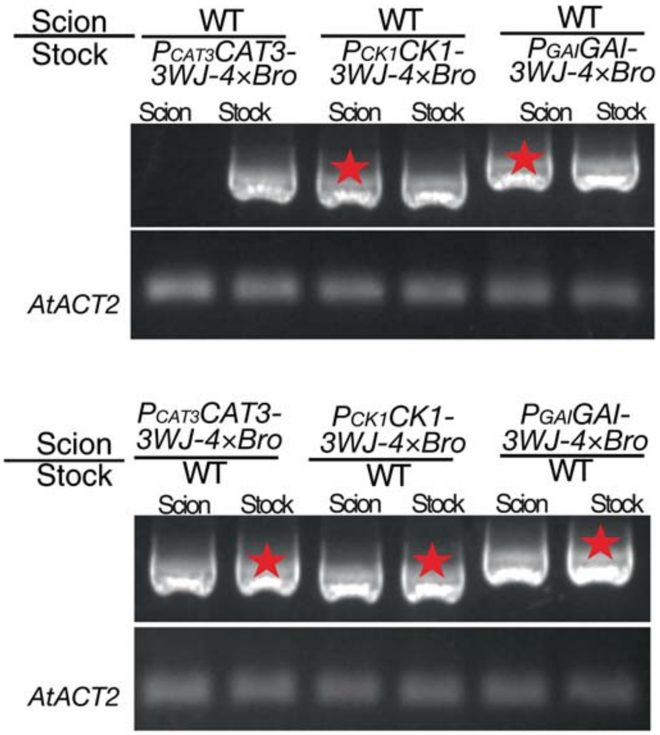

Figure EV2. The RT–PCR analyses of 3WJ-4×Bro-tagged transcripts expressed under native promoters in *A. thaliana*.

The RNAs were extracted from *A. thaliana* (Col-0) stocks (grafted with transgenic scions), and in the stem tip of wild-type scions (grafted onto transgenic stocks). The root samples were collected 1.5 cm away from the grafting junction at 14 days post-grafting. The gene *A. thaliana ACTIN2* (*AtACT2*) was used as a control. The red five-pointed stars are placed on the gel electrophoresis image to indicate where the fusion transcripts were successfully detected in wild-type plants.

 © The Author(s)

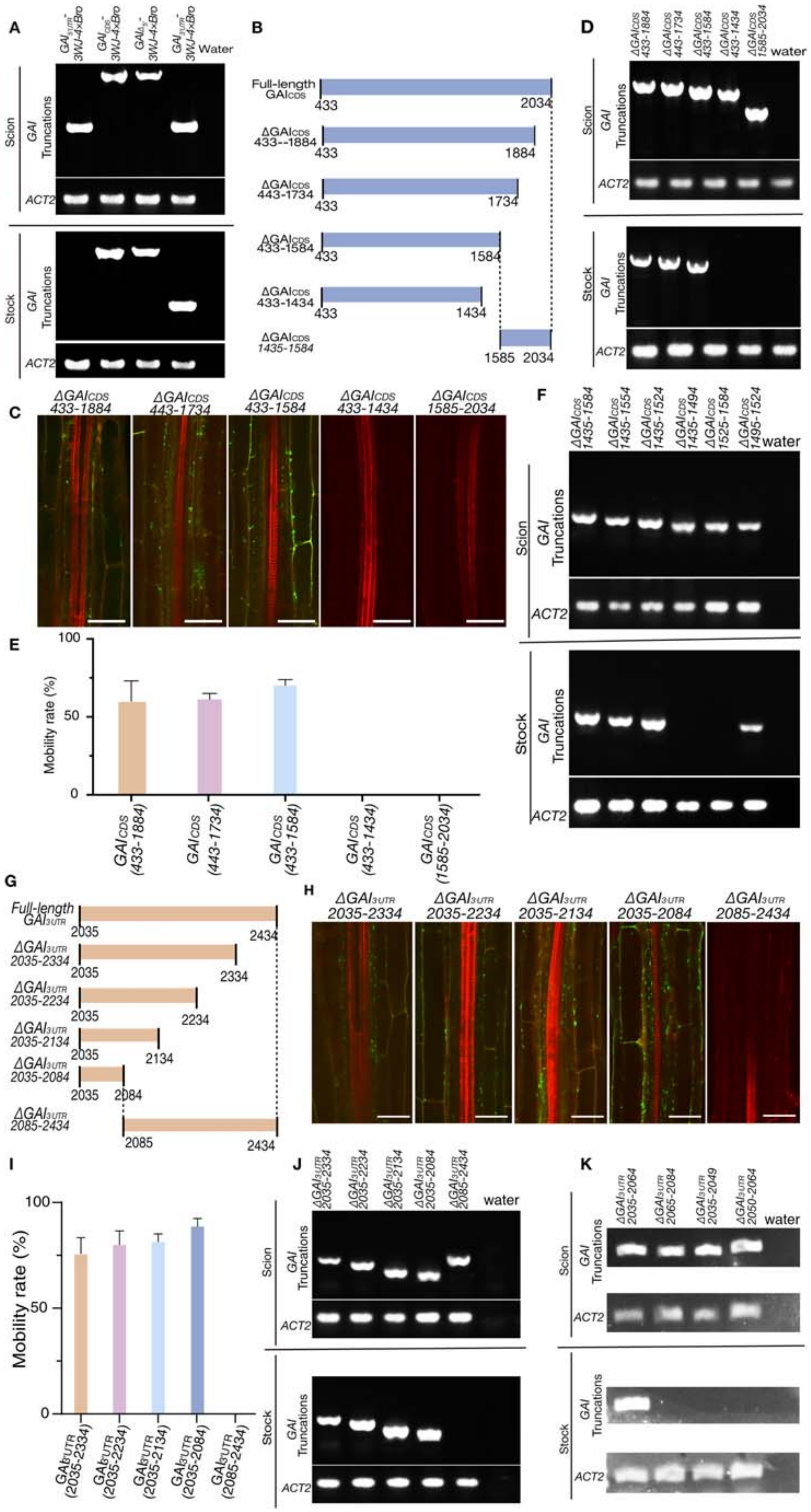
A
Water
Scion
GAI Truncations
ACT2
Stock
B
Full-length GAIcds
433
2034
ΔGAIcds 433--1884
1884
ΔGAIcds 443-1734
1734
ΔGAIcds 433-1584
1584
ΔGAIcds 433-1434
1434
ΔGAIcds 1435-1584
1585
D
ΔGAIcds 433-1884
ΔGAIcds 443-1734
ΔGAIcds 433-1584
ΔGAIcds 433-1434
ΔGAIcds 1585-2034
water
C
ΔGAIcds 1585-2034
F
ΔGAIcds 1435-1584
ΔGAIcds 1435-1554
ΔGAIcds 1435-1524
ΔGAIcds 1435-1494
ΔGAIcds 1525-1584
ΔGAIcds 1495-1524
E
100
50
0
Mobility rate (%)
GAIcds (433-1884)
GAIcds (443-1734)
GAIcds (433-1584)
GAIcds (433-1434)
GAIcds (1585-2034)
G
Full-length GAI3UTR
2035
2434
ΔGAI3UTR 2035-2334
2334
ΔGAI3UTR 2035-2234
2234
ΔGAI3UTR 2035-2134
2134
ΔGAI3UTR 2035-2084
2084
ΔGAI3UTR 2085-2434
2085
H
I
J
ΔGAI3UTR 2035-2334
ΔGAI3UTR 2035-2234
ΔGAI3UTR 2035-2134
ΔGAI3UTR 2035-2084
ΔGAI3UTR 2085-2434
K
ΔGAI3UTR 2035-2064
ΔGAI3UTR 2065-2084
ΔGAI3UTR 2035-2049
ΔGAI3UTR 2050-2064
GAI3UTR (2035-2334)
GAI3UTR (2035-2234)
GAI3UTR (2035-2134)
GAI3UTR (2035-2084)
GAI3UTR (2085-2434)

Figure EV3. Identification of mobile motifs in *GAI* mRNA using the 3WJ-4×Bro tag.

(**A**) RT–PCR detection of *GAI* deletion transcripts in WT *A. thaliana* rootstocks grafted with *pUBQ::GAI* deletion variant scions. Samples were collected 1.5 cm below the graft junction at 14 days post-grafting. *AtACT2* was used as the reference gene. (**B**) Schematic representation of the GAI_{CDS} truncation strategy. (**C**) Representative confocal images of GAI_{CDS} truncation variants in WT *A. thaliana* rootstocks grafted with *pUBQ::GAI* mutant scions. Cell boundaries are indicated by PI staining (red). Scale bars, 25 μm. (**D**) RT–PCR detection of GAI_{CDS} truncation transcripts in WT rootstocks. *AtACT2* was used as the reference gene. (**E**) Quantification of fluorescence detection frequencies in WT rootstocks grafted with *pUBQ::GAI*$_{CDS}$ truncation variant scions. (**F**) RT–PCR detection of GAI_{CDS} fine-mapping constructs in WT rootstocks. *AtACT2* was used as the reference gene. (**G**) Schematic representation of the $GAI_{3'UTR}$ truncation strategy. (**H**) Representative confocal images of $GAI_{3'UTR}$ truncation variants in WT *A. thaliana* rootstocks grafted with *pUBQ::GAI* mutant scions. Cell boundaries are indicated by PI staining (red). Scale bars, 25 μm. (**I**) Quantification of fluorescence detection frequencies in WT rootstocks grafted with *pUBQ::GAI*$_{3'UTR}$ truncation variant scions. (**J**) RT–PCR detection of $GAI_{3'UTR}$ truncation transcripts in WT rootstocks. *AtACT2* was used as the reference gene. (**K**) RT–PCR detection of $GAI_{3'UTR}$ fine-mapping constructs in WT rootstocks. *AtACT2* was used as the reference gene. For (**E**, **I**), 15 independent grafts were examined for each construct under identical confocal imaging settings. A plant was scored as fluorescence-positive when a clear 3WJ-4×Bro signal was detected in the WT rootstock. Mobility rates were calculated as the percentage of fluorescence-positive plants among the total number of plants examined. Data are shown as mean ± SD from five independent transformants. Each experiment was repeated three times with similar results.

© The Author(s)

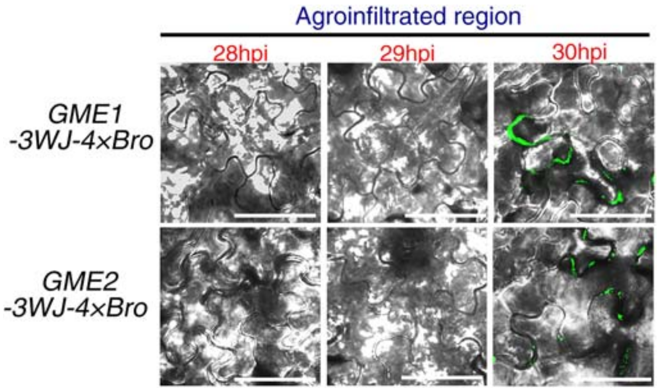

Figure EV4. Representative imaging of 3WJ-4×Bro-tagged the two mobile motifs in the agroinfiltrated leaf region of *N. benthamiana* after Agrobacterium inoculation.

Fluorescence monitoring was initiated at the *N. benthamiana* inoculation site starting at 28 h post-inoculation (hpi), with hourly observations thereafter. Scale bars represent 50 µm (leaf). The experiments above were repeated three times independently.

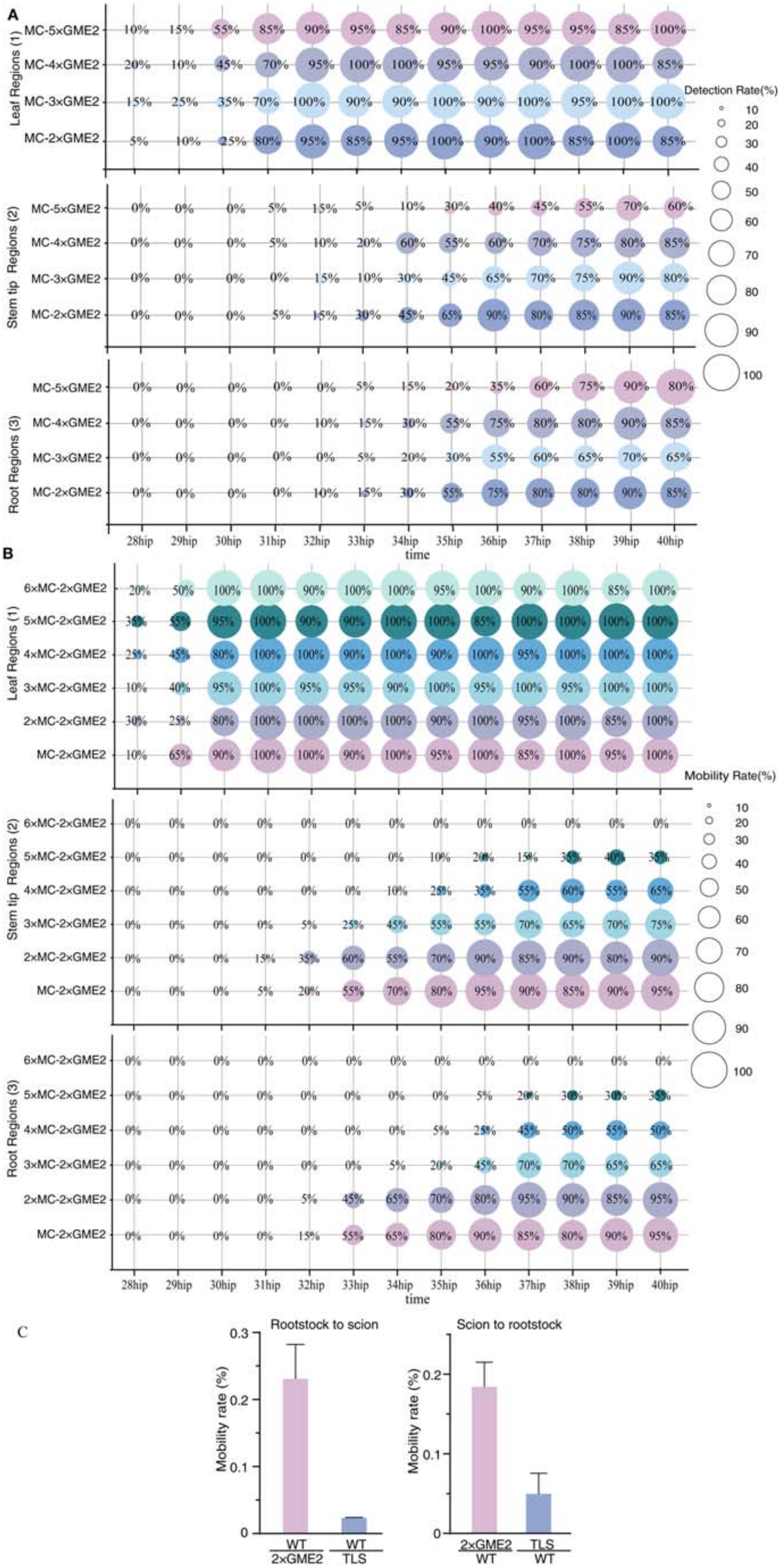
A
Leaf Regions (1)
MC-5×GME2 10% 15% 55% 85% 90% 95% 85% 90% 100% 95% 95% 85% 100%
MC-4×GME2 20% 10% 45% 70% 95% 100% 100% 95% 95% 90% 100% 100% 85%
MC-3×GME2 15% 25% 35% 70% 100% 90% 90% 100% 90% 100% 95% 100% 100%
MC-2×GME2 5% 10% 25% 80% 95% 85% 95% 100% 90% 100% 85% 100% 85%
Stem tip Regions (2)
MC-5×GME2 0% 0% 0% 5% 15% 5% 10% 30% 40% 45% 55% 70% 60%
MC-4×GME2 0% 0% 0% 5% 10% 20% 60% 55% 60% 70% 75% 80% 85%
MC-3×GME2 0% 0% 0% 0% 15% 10% 30% 45% 65% 70% 75% 90% 80%
MC-2×GME2 0% 0% 0% 5% 15% 30% 45% 65% 90% 80% 85% 90% 85%
Root Regions (3)
MC-5×GME2 0% 0% 0% 0% 0% 5% 15% 20% 35% 60% 75% 90% 80%
MC-4×GME2 0% 0% 0% 0% 10% 15% 30% 55% 75% 80% 80% 90% 85%
MC-3×GME2 0% 0% 0% 0% 0% 5% 20% 30% 55% 60% 65% 70% 65%
MC-2×GME2 0% 0% 0% 0% 10% 15% 30% 55% 75% 80% 80% 90% 85%
28hip 29hip 30hip 31hip 32hip 33hip 34hip 35hip 36hip 37hip 38hip 39hip 40hip
time
Detection Rate(%)
10 20 30 40 50 60 70 80 90 100
B
Leaf Regions (1)
6×MC-2×GME2 20% 50% 100% 100% 90% 100% 100% 95% 100% 90% 100% 85% 100%
5×MC-2×GME2 35% 55% 95% 100% 90% 90% 100% 100% 85% 100% 100% 100% 100%
4×MC-2×GME2 25% 45% 80% 100% 100% 90% 100% 90% 100% 95% 100% 100% 100%
3×MC-2×GME2 10% 40% 95% 100% 95% 95% 90% 100% 95% 100% 95% 100% 100%
2×MC-2×GME2 30% 25% 80% 100% 100% 100% 100% 90% 100% 95% 100% 85% 100%
MC-2×GME2 10% 65% 90% 100% 100% 90% 100% 95% 100% 85% 100% 95% 100%
Stem tip Regions (2)
6×MC-2×GME2 0% 0% 0% 0% 0% 0% 0% 0% 0% 0% 0% 0% 0%
5×MC-2×GME2 0% 0% 0% 0% 0% 0% 0% 10% 20% 15% 35% 40% 35%
4×MC-2×GME2 0% 0% 0% 0% 0% 0% 10% 25% 35% 55% 60% 55% 65%
3×MC-2×GME2 0% 0% 0% 0% 5% 25% 45% 55% 55% 70% 65% 70% 75%
2×MC-2×GME2 0% 0% 0% 15% 35% 60% 55% 70% 90% 85% 90% 80% 90%
MC-2×GME2 0% 0% 0% 5% 20% 55% 70% 80% 95% 90% 85% 90% 95%
Root Regions (3)
6×MC-2×GME2 0% 0% 0% 0% 0% 0% 0% 0% 0% 0% 0% 0% 0%
5×MC-2×GME2 0% 0% 0% 0% 0% 0% 0% 0% 5% 20% 30% 30% 35%
4×MC-2×GME2 0% 0% 0% 0% 0% 0% 0% 5% 25% 45% 50% 55% 50%
3×MC-2×GME2 0% 0% 0% 0% 0% 0% 5% 20% 45% 70% 70% 65% 65%
2×MC-2×GME2 0% 0% 0% 0% 5% 45% 65% 70% 80% 95% 90% 85% 95%
MC-2×GME2 0% 0% 0% 0% 15% 55% 65% 80% 90% 85% 80% 90% 95%
28hip 29hip 30hip 31hip 32hip 33hip 34hip 35hip 36hip 37hip 38hip 39hip 40hip
time
Mobility Rate(%)
10 20 30 40 50 60 70 80 90 100
C
Rootstock to scion
Mobility rate (%)
0.3 0.2 0.1 0
WT / 2×GME2
WT / TLS
Scion to rootstock
Mobility rate (%)
0.2 0.1 0
2×GME2 / WT
TLS / WT

Figure EV5. Performance of GME2 as a long-distance RNA delivery module.

(**A**, **B**) Statistical analysis of fluorescence-detected rates at three *N. benthamiana* sampling sites (regions 1, 2, and 3) following *Agrobacterium* inoculation with different copy numbers of GME2/mCherry constructs. *X* axis: Time post-inoculation; *Y* axis: Agrobacterium inoculation with six constructs in leaves. $n = 20$ plants per construct per time point. Mobility rates were calculated as described in "Methods". (**C**) 15 independent grafts were examined for each construct under identical confocal imaging settings. A plant was scored as fluorescence-positive when a clear 3WJ-4×Bro signal was detected in the WT scion (left panel) and in the rootstock (right panel). The mobility rates were calculated as the percentage of fluorescence-positive plants among the total number of plants examined. Bars represent the mean ± SD. Five independent transformants were analyzed per construct, and all experiments were repeated three times with similar results.

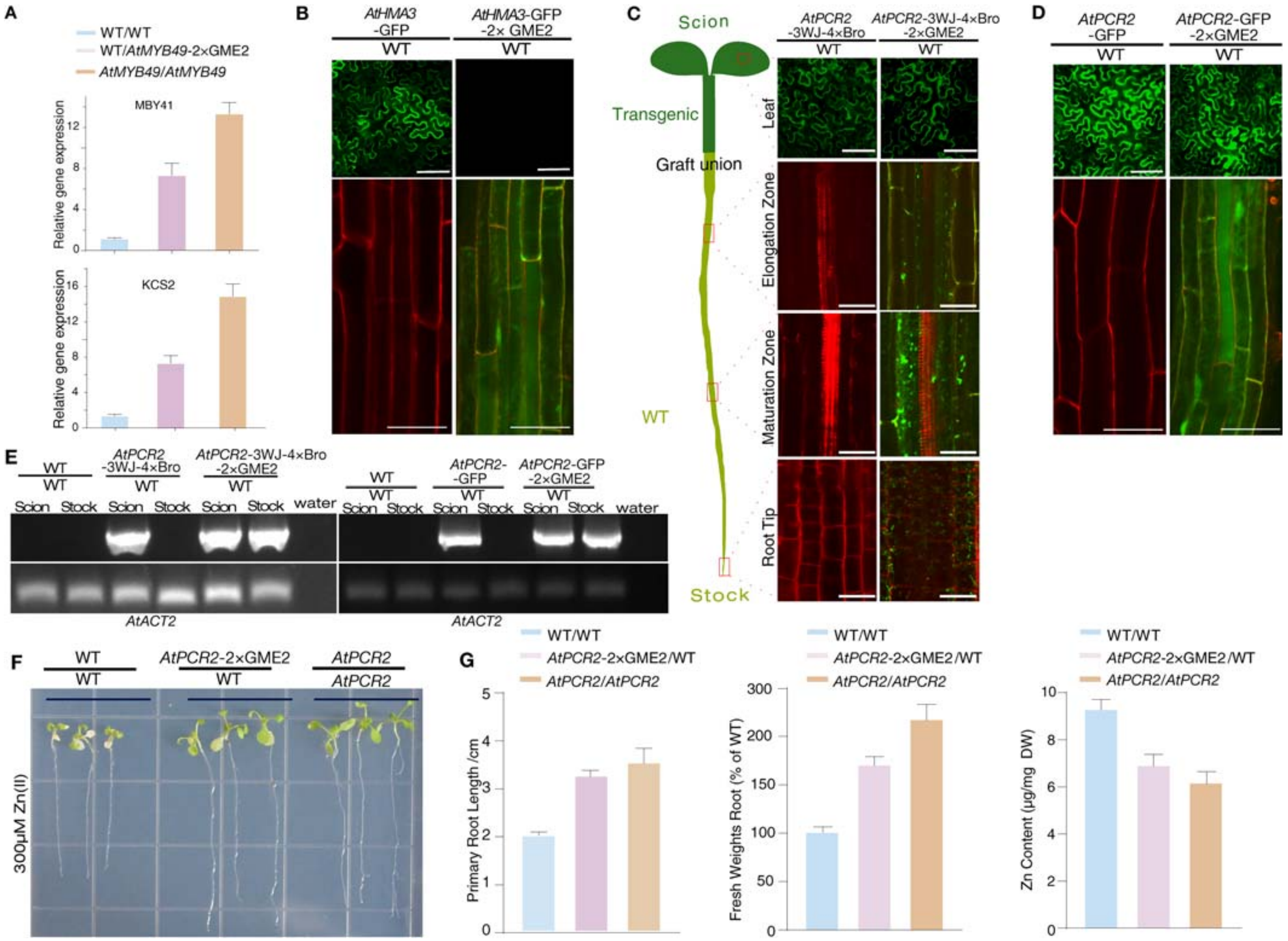

Figure EV6. 2×GME2-mediated delivery of functional mRNAs enhances stress tolerance in grafted *A. thaliana*.

(**A**) Quantitative RT–PCR detection of the expression levels of two *MYB49*-regulated genes *MYB41* and *KCS2* in scions. *AtACT2* was used as the reference gene. (**B**) Representative confocal images of scion and rootstock showing the distribution of *HMA3*-GFP protein in grafted plants. (**C**) Representative confocal images of different root regions showing the distribution of the mobile *PCR2*-3WJ-4×Bro RNA in the roots of grafted plants. (**D**) Representative confocal images of scion and rootstock showing the distribution of *PCR2*-GFP protein grafted in plants. (**E**) RT–PCR detection of 3WJ-4×Bro- or GFP- tagged *PCR2* RNA in rootstocks and scions. *AtACT2* was used as the reference gene for RNA quality control, and water was used as the negative template control. (**F**) Phenotypes of graft combinations WT/WT, *AtPCR2*-2×GME2/WT, and *AtPCR2/AtPCR2* grown on 1/2 MS medium containing 300 μM Zn for two weeks. Scale bars, 15 mm. (**G**) Quantification of primary root length (cm), root fresh weight (g), and root Zn^{2+} content for the indicated graft combinations after two weeks of treatment ($n = 3$ biological replicates; 12 plants per genotype per replicate). All experiments were repeated three times with similar results.

 © The Author(s)

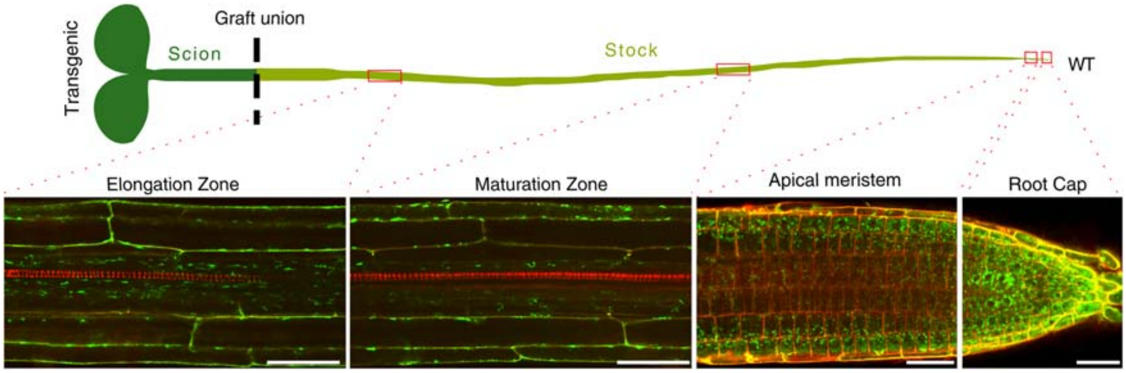

Figure EV7. Confocal fluorescence detection of GME2-3WJ-4×Bro RNA in recipient roots after grafting.

Representative longitudinal optical section images of roots showing the distribution of 3WJ-4×Bro-GME2 RNA in different root regions at 14 days post-grafting (> 2 independent transgenic lines per construct, each *n* > 10 plants). Cell boundaries are indicated by PI staining (red). Scale bars, 25 μm.

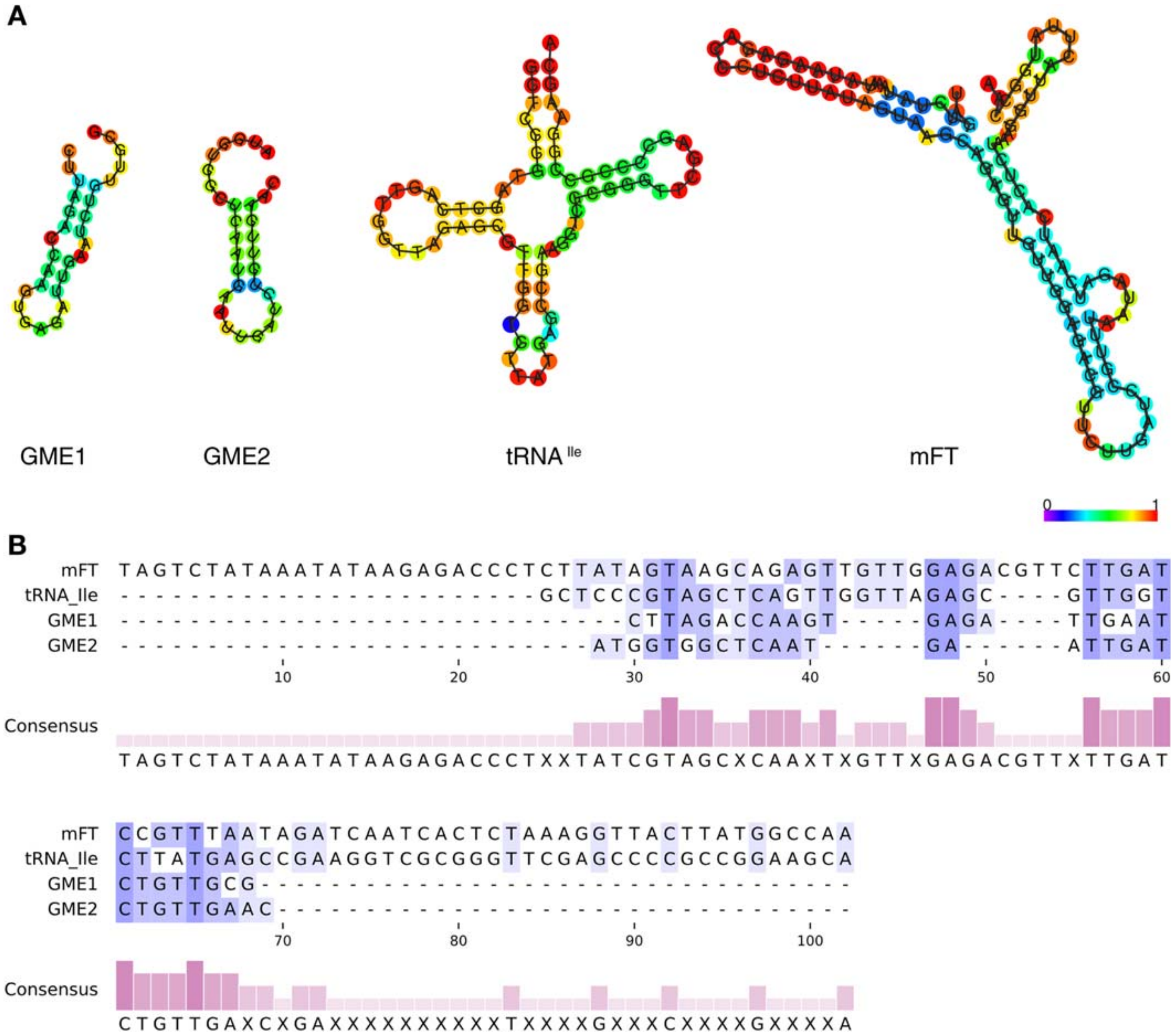

Figure EV8. Predicted RNA secondary structures and nucleotide sequence alignment of GME1, GME2, tRNA^{Ile}, and mFT.

(**A**) RNA secondary structures of GME1, GME2, tRNA^{Ile}, and mFT were predicted using the RNAfold web server (http://rna.tbi.univie.ac.at/cgi-bin/RNAWebSuite/RNAfold.cgi). (**B**) Multiple nucleotide sequence alignment of mFT, tRNA^{Ile}, GME1, and GME2. Conserved or similar nucleotides are highlighted in blue, and gaps introduced for alignment are indicated by dashes. The consensus sequence and histogram below summarize nucleotide conservation across aligned positions.

 © The Author(s)

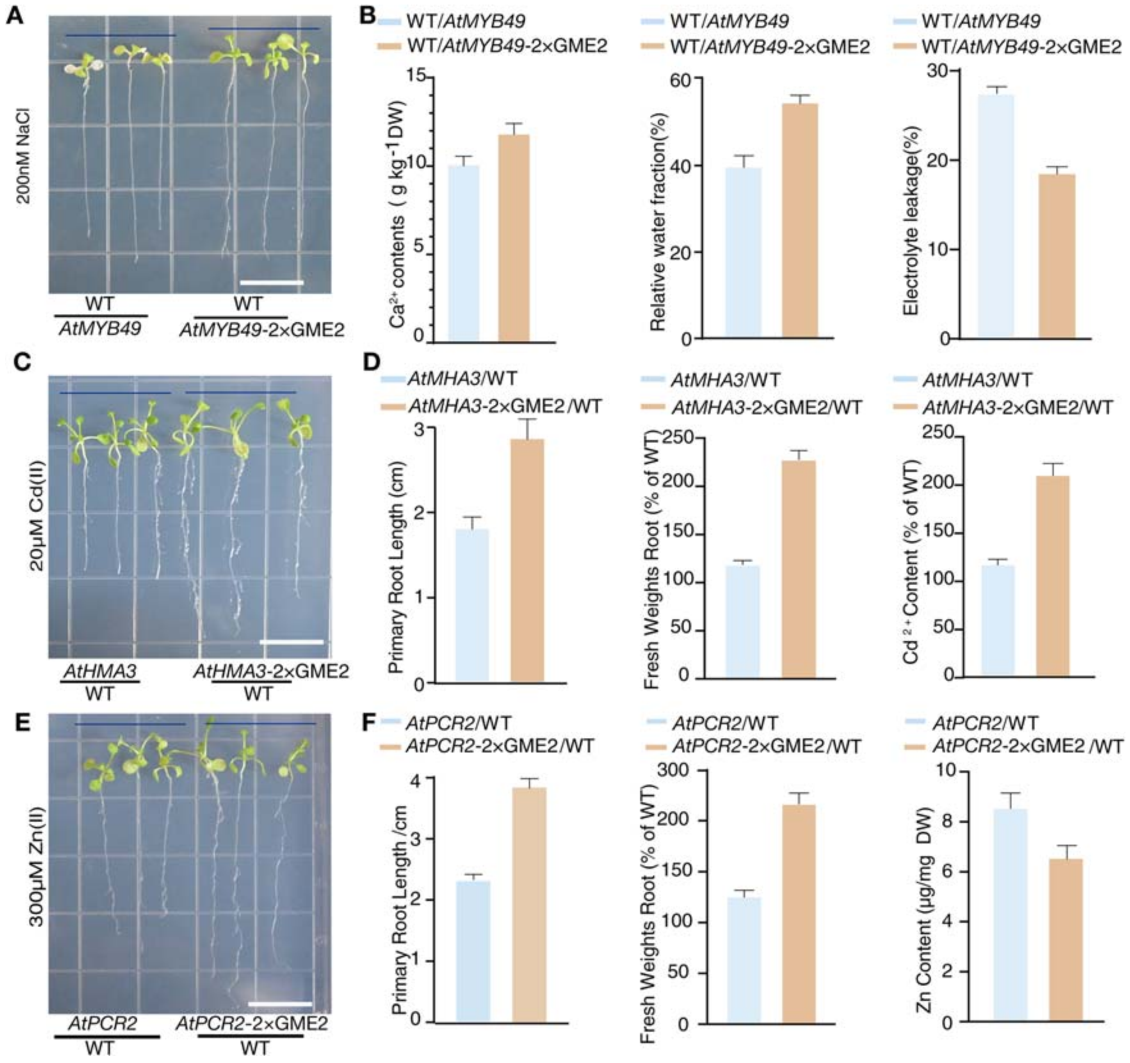

Figure EV9. Comparison of 2×GME2-tagged graft combinations with corresponding grafts lacking 2×GME2 under stress conditions.

(**A**) Phenotypes of WT/*AtMYB49* and WT/*AtMYB49*-2×GME2 graft combinations grown on 1/2 MS medium supplemented with 200 mM NaCl for 14 days. Scale bar, 10 mm. (**B**) Quantification of leaf Ca^{2+} content, relative water content, and electrolyte leakage in WT/*AtMYB49* and WT/*AtMYB49*-2×GME2 graft combinations after NaCl treatment. (**C**) Phenotypes of *AtHMA3*/WT and *AtHMA3*-2×GME2/WT graft combinations grown on 1/2 MS medium containing 20 μM Cd for two weeks. Scale bar, 10 mm. (**D**) Quantification of primary root length, root fresh weight, and root Cd content in *AtHMA3*/WT and *AtHMA3*-2×GME2/WT graft combinations after Cd treatment. (**E**) Phenotypes of *AtPCR2*/WT and *AtPCR2*-2×GME2/WT graft combinations grown on 1/2 MS medium containing 300 μM Zn for two weeks. Scale bar, 10 mm. (**F**) Quantification of primary root length, root fresh weight, and root Zn content in *AtPCR2*/WT and *AtPCR2*-2×GME2/WT graft combinations after Zn treatment. Data are shown as mean ± s.d.; $n = 3$ biological replicates, with 12 plants per graft combination per replicate. All experiments were repeated three times with similar results.

© The Author(s)
