# Supplementary material for: Developing a mobile RNA delivery system for grafting trait improvement
Source: EMBO Rep. 2026 Jun 10;27(13):3863–89. doi: 10.1038/s44319-026-00819-z (PMC13354801; doi:10.1038/s44319-026-00819-z)
Supplement: Supplementary file 2 — Peer Review File [file 44319_2026_819_MOESM2_ESM.pdf]

EMBO reports

Developing a Mobile RNA Delivery System for Grafting Trait Improvement

Yiran Tao, Jiayu zhang, Yuyang Zhang, Miaomiao Lei, Xuan Dong, Qichong Li, JiuYuan Bai, and Yun Zhao

Corresponding author(s): Yun Zhao (zhaoyun@scu.edu.cn) , JiuYuan Bai (Baijiuy@scu.edu.cn)

Review Timeline:

Submission Date:	2nd Dec 25
Editorial Decision:	15th Jan 26
Revision Received:	31st Mar 26
Editorial Decision:	13th May 26
Revision Received:	17th May 26
Accepted:	22nd May 26

Editor: Esther Schnapp

Transaction Report:

(Note: With the exception of the correction of typographical or spelling errors that could be a source of ambiguity, letters and reports are not edited. Depending on transfer agreements, referee reports obtained elsewhere may or may not be included in this compilation. Referee reports are anonymous unless the Referee chooses to sign their reports.)

1st Editorial Decision **15th Jan 2026**

Dear Dr. Zhao,

Thank you for the submission of your manuscript to EMBO reports. We have now received the full set of referee reports that is pasted below.

As you will see, the referees acknowledge that the findings are interesting. However, they also have several suggestions for how the study could be improved and strengthened. I think all suggestions are good and should be addressed, but please let me know in case you disagree, and we can discuss the exact revision requirements further, also in a video chat, if you like.

I would thus like to invite you to revise your manuscript with the understanding that the referee concerns must be fully addressed and their suggestions taken on board. Please address all referee concerns in a complete point-by-point response. Acceptance of the manuscript will depend on a positive outcome of a second round of review. It is EMBO reports policy to allow a single round of major revision only and acceptance or rejection of the manuscript will therefore depend on the completeness of your responses included in the next, final version of the manuscript.

We realize that it is difficult to revise to a specific deadline. In the interest of protecting the conceptual advance provided by the work, we recommend a revision within 3 months (17th Apr 2026). Please discuss the revision progress ahead of this time with the editor if you require more time to complete the revisions.

IMPORTANT NOTE: we perform an initial quality control of all revised manuscripts before re-review. Your manuscript will FAIL this control and the handling will be DELAYED if the following APPLIES:
1) A data availability section providing access to data deposited in public databases is missing. If you have not deposited any data, please add a sentence to the data availability section that explains that.
2) Your manuscript contains statistics and error bars based on n=2. Please use scatter blots in these cases. No statistics should be calculated if n=2.

When submitting your revised manuscript, please review the submission guidelines (https://link.springer.com/journal/44319/submission-guidelines#cms-Revised-submissions) and carefully review the instructions that follow below. Failure to include requested items will delay the evaluation of your revision.

1) a .docx formatted version of the manuscript text (including legends for main figures, EV figures and tables). Please make sure that the changes are highlighted to be clearly visible.

2) individual production quality figure files as .eps, .tif, .jpg (one file per figure). See hhttps://media.springernature.com/original/springer-cms/rest/v1/content/27825798/data/v1 for more info on how to prepare your figures.

3) We replaced Supplementary Information with Expanded View (EV) Figures and Tables that are collapsible/expandable online. A maximum of 5 EV Figures can be typeset. EV Figures should be cited as 'Figure EV1, Figure EV2" etc... in the text and their respective legends should be included in the main text after the legends of regular figures.

- For the figures that you do NOT wish to display as Expanded View figures, they should be bundled together with their legends in a single PDF file called *Appendix*, which should start with a short Table of Content. Appendix figures should be referred to in the main text as: "Appendix Figure S1, Appendix Figure S2" etc. See 'Expanded View' section of the submission guidelines.

- Additional Tables/Datasets should be labeled and referred to as Table EV1, Dataset EV1, etc. Legends have to be provided in a separate tab in case of .xls files. Alternatively, the legend can be supplied as a separate text file (README) and zipped together with the Table/Dataset file.

4) a .docx formatted letter INCLUDING the reviewers' reports and your detailed point-by-point responses to their comments. As part of the EMBO Press transparent editorial process, the point-by-point response is part of the Review Process File (RPF), which will be published alongside your paper.

5) a complete author checklist, which you can download from our author guidelines. Please insert information in the checklist that is also reflected in the manuscript. The completed author checklist will also be part of the RPF.

6) Please note that all corresponding authors are required to supply an ORCID ID for their name upon submission of a revised manuscript (<https://orcid.org/>), which can be linked to your profile in the manuscript submission system.

7) Before submitting your revision, primary datasets produced in this study need to be deposited in an appropriate public database. Please remember to provide a reviewer password if the datasets are not yet public. The accession numbers and database should be listed in a formal "Data Availability" section placed after Methods. Please note that the Data Availability

Section is restricted to new primary data that are part of this study. * Note - All links should resolve to a page where the data can be accessed. *

If your study has not produced novel datasets, please mention this fact in the Data Availability Section.

8) At EMBO Press we ask authors to provide source data for the main manuscript figures. You will receive a separate email with instructions for providing source data with your revised manuscript, including how to upload and organize the files.

9) Our journal also encourages inclusion of *data citations in the reference list* to directly cite datasets that were re-used and obtained from public databases. Data citations in the article text are distinct from normal bibliographical citations and should directly link to the database records from which the data can be accessed. In the main text, data citations are formatted as follows: "Data ref: Smith et al, 2001" or "Data ref: NCBI Sequence Read Archive PRJNA342805, 2017". In the Reference list, data citations must be labeled with "[DATASET]". A data reference must provide the database name, accession number/identifiers and a resolvable link to the landing page from which the data can be accessed at the end of the reference.

10) Regarding data quantification, the following points must be specified in each figure legend:

- the name of the statistical test used to generate error bars and P values,

- the number (n) of independent experiments (please specify technical or biological replicates) underlying each data point,

- the nature of the bars and error bars (s.d., s.e.m.),

- If the data are obtained from n Program fragment delivered error ``Can't locate object method "less" via package "than" (perhaps you forgot to load "than"?) at //ejpvfs23/sites23b/embor_www/letters/embor_decision_revise_and_review.txt line 54.' 2, use scatter blots showing the individual data points.

Discussion of statistical methodology can be reported in the materials and methods section, but figure legends should contain a basic description of n, P and the test applied.

- Please also include scale bars in all microscopy images.

11) The journal requires a statement specifying whether or not authors have competing interests (defined as all potential or actual interests that could be perceived to influence the presentation or interpretation of an article). In case of competing interests, this must be specified in your disclosure statement.

12) All Materials and Methods need to be described in the main text using our 'Structured Methods' format, which is required for all research articles. According to this format, the Methods section includes a separate Reagents and Tools Table file (listing key reagents, experimental models, software and relevant equipment and including their sources and relevant identifiers) and a Methods and Protocols section describing the methods using a step-by-step protocol format. The aim is to facilitate adoption of the methodologies across labs. More information on how to adhere to this format as well as a downloadable template (.docx) for the Reagents and Tools Table can be found in our author guidelines.

We would also welcome the submission of cover suggestions, or motifs to be used by our Graphics Illustrator in designing a cover.

As part of the EMBO publication's Transparent Editorial Process, EMBO reports publishes online a Review Process File (RPF) to accompany accepted manuscripts. This File will be published in conjunction with your paper and will include the referee reports, your point-by-point response and all pertinent correspondence relating to the manuscript.

You are able to opt out of this by letting the editorial office know (emboreports@embo.org). If you do opt out, the Review Process File link will point to the following statement: "No Review Process File is available with this article, as the authors have chosen not to make the review process public in this case."

I look forward to seeing a revised form of your manuscript when it is ready.

Kind regards,
Esther

Esther Schnapp, PhD
Senior Editor
EMBO reports

Referee #1:

In the manuscript titled " Developing a Mobile RNA Delivery System for Grafting Trait Improvement" by Tao et al. the authors represent a method to trace mobile mRNAs after agroinfiltration and in grafted plants using 3WJ-4.Bro tagged transcripts. They tested and implemented the system with published mobile mRNAs such as GAI, CK1, CAT3, and TCTP1 and identified two RNA sequences (GME1 and GME2) present in GAI mediating mRNA transport. Furthermore they fused two GME2 sequences to the 3'UTR of mCherry-3WJ-4×Bro-2 (mCherry-3WJ-4×Bro-2×GME2) which showed increased transcript delivery compared to a 1x GME2 fusion. Also, they compared it with the tRNAMet -based TLS delivery module and present evidence that 2×GME2 constructs exhibit an increased delivery velocity and fluorescence after transport when compared with the TLS fusion.

Suggestions/ Comments

- The statement "...we systematically re-evaluate 100 RNA-Seq-predicted mobile mRNAs and identify only three ..." has to be clarified/rephrased (see also minor comments). The tested transcripts are still fusion constructs suffering the same pitfalls the authors refer to by using fusions: "...may perturb RNA conformation or trafficking due to large protein tags30,31 " .
Here a table of tested transcripts has to be presented. Note that with such a statement one should be cautious as many transcripts might only move directional (as shown e.g. by Thieme et al.) or their mobility is species/condition specific (e.g. not moving in N. benthamiana). Thus, this should be rephrased and specific information on the tested transcripts should to be given in form of a supplemental table.

- Figure 1B confocal images region 1,2 and 3: The majority of the signal appears to be present in the apoplast and not within the cells (especially in infiltrated leaves, region1). Please explain and show higher resolution/quality images allowing to see the distribution of the fluorescent signal within the cells. Especially as one would expect that mRNA is detectable in the cytosol after expression in transfected cells (region 1) and after expected symplastic delivery (regions 2 and 3).

- Control experiment missing in the FISH experiment shown in Figure 1 E. Here no mock infiltrated plant tissue (w/o TCTP) is shown confirming the specificity of the use FISH probe. This has to be implemented.

- Extent of transport is not shown in grafted plants. TCTP1 is reported to move from transgenic shoots mainly to root tips and remains in close proximity of the phloem vasculature (see Yang et al). Do the authors observe a similar distribution of their fusion construct? Thus, an image showing the root tip and the different presence in different root regions should be shown. Also, the presence of tagged TCTP1 transcript in the grafted root should be evaluated by RT-PCR assays.

- In general, the authors suggest that their identified GME2 motif outperforms a TLS motif-based system. However, they do not show any comparative data concerning mobility in grafted plants and whether the GME2 motif mediates mobility from roots to above ground tissues / flowers like the TLS motif does.

- As with tagged TCTP1 transcript: Can a GME2 delivered tagged transcript be detected in the root tips and in the vasculature of grafted plants? Such images should be shown and discussed.

- Experiment with GFP-tagged mobile MYB49-GME2 and distribution of the GFP tagged mobile construct in the expressing and recipient roots/shoots. One would expect that the MYB transcription factor is also found in the nucleus and is not evenly distributed in the cytosol of the cells as shown in the figure 6D confocal images. Is this a consistent pattern based on the fusion construct? Again, here one should show more and higher quality images of different root cells / regions showing the distribution of the mobile construct in the roots. Also, one should confirm the mobility of the transcript by RT -PCR assays. Also, as MYP49 is shown to change the expression of known target genes one should use quantitative RT-PCR assays to confirm that the mobile transcript results in a change of expression in roots confirming it is functional in recipient tissues.

- Figure 5G and efficiency of transport compared to the TLS motif. The authors write in the results that "Importantly, the distal fluorescence detection rate with 2×GME2 increased by ~24-fold in region 2 and ~8-fold in region 3 compared with the TLS system (Figure 5G)." However, figure 5 D only shows the fluorescence intensity in region 2 and 3 of 5xCherry-TLS (according to the legend). Thus, these data are missing / not presented. Also, no image is provided allowing to evaluate the expression/ distribution of mCherry fluorescence in the infiltrated plants. As the authors state in the text mCherry is expressed in the recipient cells. Thus, it should be possible to detect it under a CLSM and present such images.

- The discussion text makes strong statements such as"... this work establishes a modular, sequence-defined RNA mobility system that directly addresses the lack of efficient, broadly applicable, non-viral carriers for graft-mediated trait improvement38,39."

However, this statement is underlined with "strange" references which are not really relevant for this work. One (Yang et al) shows a sequence-defined RNA mobility /editing system. Here do make such a statement one has to compare the known editing efficiency of the existing TLS based editing system with a GME2 based system in the context of Cas9 - which was not done.

This would be in particular important as it was not shown with the presented GME2 motif that it can mediate delivery to the apical meristematic tissues forming the offspring. In similar line, the other manuscript cited is a review focusing on m6A modifications and does not addressing RNA mobility.

- In a similar line, in the following paragraph the authors imply that the GME2 mobility motif may be better than the TLS system without any evidence and claim that "TLS-based graft-mobile editing systems [.....] require expression of bulky CRISPR components in rootstocks and often depend on strong selection to retrieve rare editing events". This claim is no substantiated in their presented results as e.g. no movement mediated by the GME2 to flowers or tissue forming offspring was confirmed, no Cas9 fusion was tested, ...etc.. Thus, these statements should be adopted/ rephrased / changed or deleted accordingly. Again, the authors claim referring to the TLS system that "By contrast, 2×GME2 is a short, sequence-defined cassette..." This claim is not supported by data as the TLS system has a similar length (of approx. 40-35 bases) and has a short, defined sequence (between 70 to 40 bases) which was shown to mediate movement of non-mobile, long and short transcripts. Here the authors over emphasize their finding of an interesting new motif which might be better suitable or not. In general, such statements appear not helpful and do not underscore their interesting findings and data, especially if they are not substantiated by proper experiments.

Minor suggestions/corrections:

Introduction:
Statement "...proof-of-concept studies have demonstrated grafting-induced, transgene-free genome editing using TLS or virus-based systems, these approaches still suffer from low editing efficiency, restricted cargo capacity, and biosafety concerns associated with viral vectors.". Should be rephrased as "restricted cargo capacity, and biosafety concerns" only apply to viral-based systems.

The statement that "Up to now, only three mobile mRNA motifs...." Were found is not right. Others were identified e.g. in AtHSC70.1 (the SVR in the 3' ORF) and in TCTP1 (in the 5' ORF). Also, the statement that m5C and m6A are motifs seems to be an oversimplification of the reported studies. For example, HSC70.1 and TCTP1 mobility appear to depend on m5C modifications in their respective motif regions but can act a mobility motifs of as shown with fusion constructs.

Correct the citation in "... as could support non-transgenic precision breeding via graft-mediated RNA delivery33 ." Cite the first report/suggestions on graft mediated RNA delivery and precision breeding (e.g. Zhang et al 2016 Plant Cell and/or Yang et al 2023 Nature Biotech.)

Correct the citation for "CK120 " (Li, X. J. et al. m5C and m6A modifications regulate the mobility of pumpkin CHOLINE KINASE 1 mRNA under chilling stress. Plant Physiology 197, 1-16, (2024).) to 25 (Zhang, W. et al. tRNA-Related Sequences Trigger Systemic mRNA Transport in Plants.The Plant Cell 28, 1237-1249, (2016)) as Arabidopsis CK1 mobility was first reported by Thieme, C. J. et al. (Endogenous Arabidopsis messenger RNAs transported to distant tissues. Nature Plants 1, 1-9, (2015)) and by Zhang W et al. 2016. This is especially important as the authors use AtCK1 in their submitted study to confirm its mobility.

Correct the citation for the statement "...subsequent re-analyses and experimental validation have revealed high false-positive rates and have indicated that only a small fraction of predicted candidates are truly mobile over long distances29 " The correct citation should be: "Paajanen et al. Re-analysis of mobile mRNA datasets raises questions about the extent of long-distance mRNA communication. Nat Plants. 2025 May;11(5):977-984. doi: 10.1038/s41477-025-01979-x. Epub 2025 Apr 16. PMID: 40240650; PMCID: PMC12095074." Note, that this re-analysis only refers to the SNP-based mobility datasets and that they appear to have a high error rate (> 50%). Other datasets including e.g. transcripts moving from Arabidopsis to Cuscuta are not affected by such a SNP calling error based on RNAseq data.

The statement "Using this system, we systematically re-evaluate 100 RNA-Seq-predicted mobile mRNAs and identify only three bona fide long-distance mobile transcripts (CAT318, CK120, and GAI17)," This is not right as they also confirmed AtTCTP1 which was reported to be mobile in several manuscripts. Furthermore (see also major comments above), there is not list of the "100" tested transcripts presented in the results (e.g. as supplement data with references) supposedly not to be mobile. This has to be implemented and corrected.

Referee #2:

In this manuscript, Tao et al. applied mRNA imaging system to track mobile mRNA in plants and elucidated the systemic movement of CAT3, CK1, and GAI mRNAs in planta through the application of a fluorescence-aptamer-based RNA imaging tool. This study identify cis-elements governing the systemic movement of GAI mRNA and develop a new RNA delivery system, referred to as the 2xGME2 system. By employing the 2xGME2 system in conjunction with grafting techniques, the authors demonstrated that the 2xGME2 element fused-AtMYB49, AtHMA3 and AtPCR2 mRNAs can be transported from scion to rootstock or from rootstock to scion. In these grafts, tolerance to abiotic stresses were increased. Overall, it is very interesting study.

Most experiments are well designed and thoroughly documented. I provide minor comments below.

Regarding Fig. 1, it would be preferable to explain the intracellular localization of mRNA.

In line 125, Thieme et al. identified 918 transcripts as mobile mRNAs, and at least 222 of these were shown to move bidirectionally between root and shoot tissues. How were these narrowed down to 100 candidates? Additionally, the authors should provide the list of the 100 candidate mobile mRNAs.

Please show the nucleotide sequences of GME1 and GME2. Do these RNA motifs form secondary structures? In addition, are the GME motifs found in other mobile mRNA candidates?

Regarding with Fig. 5A, is the velocity of mRNA dependent on the number of GME copies? If so, does this dependence apply when there are three or more copies?

Regarding with Fig.5B and S5, how was the time course of distal fluorescence detection frequency quantified? The authors should describe the method in detail.

Regarding Lines 261-262, it is unclear what is being compared. Figure 5G does not directly correspond to the explanatory text.

Regarding AtMYB49, AtHMA3, and AtPCR2 in lines 284-285, it would be preferable to explain what genes they encode, in which tissues they function, and their roles. Otherwise, the rationale for the rootstock and scion combinations in the grafting experiments described in Lines 303-315 is unclear.

Regarding with Fig.6D, although MYB49 is a transcription factor, the MYB49-GFP signal does not appear to localize to the nucleus. What could be the reason why the signal does not appear in the nucleus? Further analysis and explanation of the intracellular localization are necessary.

The explanation in Lines 303-309 needs to include an explanation of the results of transgenic homografts shown in Fig. 6E-J. There is no explanation of what plants the labels AtMYB49, AtHMA3, and AtPCR2 in Fig. 6E-J refer to.

The unit for the NaCl concentration in Fig. 6E is incorrect and needs to be corrected.

Referee #3:

Tao et al use an aptamer-based RNA imaging system to identify novel mobilizing sequences, characterize the dynamics of mobility, and deliver non-mobile abiotic stress-responsive RNAs that enhance abiotic stress tolerance upon delivery. The paper adds substantial new insights to the field of RNA mobility. One significant point that I would like to raise is that the authors should specify which cell layers are being imaged in all of their experiments, particularly with regard to root imaging. It looks like the epidermal layer is being imaged, which is an unexpected destination for vascular-mobile RNAs. For most of the experiments, it also looks like the authors imaged halfway down the root, rather than at the root tip, where I would expect RNAs to exit the phloem transport stream. Cross sections through roots at high, mid, and root tip points would provide a more informative look at how RNAs are moving from the vasculature into the cortex and potentially epidermal tissue layers.

I've listed some additional points below:

Line 31: should be revised to "the empirical selection of rootstock scion combinations"

Line 66: The data from the screen of 100 candidates should be included as supplemental data, or this experiment should not be included in the paper.

lines 91-94: RT-PCR is an insufficient test to exclude the possibility of contaminating Agrobacterium because the Agro infection is a continuous process, and the RT-PCR is only informative for the time point when samples are collected. The authors produced stable transgenic lines for all essential pieces of evidence in this study, so the transient assays are okay for an initial screen.

An interesting follow-up computational experiment for the authors to consider: It would be useful for the authors to dig deeper into the evolutionary significance of GME1 and GME2. Are these sequences conserved in other flowering plant species? Do these sequences contain conserved motifs that are found in other mobile RNAs?

Figures:
Figure 1: The localization of the fluorescence in Fig 1B looks extracellular. Higher resolution images for this experiment would be useful to determine the localization of the mobile RNA. In addition, a supplemental figure showing negative controls under the exact same imaging conditions is necessary to separate signal from autofluorescence.

The co-localization experiments in Figure 1 provide nice substantiating evidence for the movement of 3WJ-4xBro

Lines 143-144: The authors should reference previous work characterizing GAI movement in this statement. For example, "Congruent with previous research on GAI mRNA mobility, these data demonstrate that GAI is a highly efficient long distance...."

Line 205/Figure 4: The authors should describe how they identified phloem cells in their imaging datasets.

For the experiments in Figure 5: In order to make comparative statements about the mobility of different sequences, it's necessary to quantify the source expression of the different constructs. Different levels of transient expression in the source tissue could significantly impact transport dynamics and produce misleading conclusions regarding the efficiency of different mobilizing sequences.

Methods:
It is important for the authors to provide a supplemental file including sequence information for all of the constructs used in this study. This is essential for external replicability, especially given that the results are linked to specific nucleotide sequences.

The authors need to include ecotype/genotype information in the materials and methods section.

Please provide more details regarding the Arabidopsis micro grafting protocol.

Dear reviewers,

Thank you for your careful review and constructive suggestions regarding our manuscript entitled “Developing a Mobile RNA Delivery System for Grafting Trait Improvement” (previous manuscript ID: EMBOR-2025-63321V1). We have revised the manuscript in accordance with the comments and marked all the changes on our revised manuscript by red. Here are the point-by-point responses for the reviewers’ comments. The responses are marked by blue.

Referee #1:

In the manuscript titled " Developing a Mobile RNA Delivery System for Grafting Trait Improvement" by Tao et al. the authors represent a method to trace mobile mRNAs after agroinfiltration and in grafted plants using 3WJ-4.Bro tagged transcripts. They tested and implemented the system with published mobile mRNAs such as GAI, CK1, CAT3, and TCTP1 and identified two RNA sequences (GME1 and GME2) present in GAI mediating mRNA transport. Furthermore they fused two GME2 sequences to the 3'UTR of mCherry-3WJ-4×Bro-2 (mCherry-3WJ-4×Bro-2×GME2) which showed increased transcript delivery compared to a 1x GME2 fusion. Also, they compared it with the tRNAMet -based TLS delivery module and present evidence that 2×GME2 constructs exhibit an increased delivery velocity and fluorescence after transport when compared with the TLS fusion.

Suggestions/ Comments

- The statement "...we systematically re-evaluate 100 RNA-Seq-predicted mobile mRNAs and identify only three ..." has to be clarified/rephrased (see also minor comments). The tested transcripts are still fusion constructs suffering the same pitfalls the authors refer to by using fusions: "...may perturb RNA conformation or trafficking due to large protein tags30,31 " .
Here a table of tested transcripts has to be presented. Note that with such a statement one should be cautious as many transcripts might only move directional (as shown e.g. by Thieme et al.) or their mobility is species/condition specific (e.g. not moving in N. benthamiana). Thus, this should be rephrased and specific information on the tested transcripts should to be given in form of a supplemental table.

Response：Thank you for this important comment. We agree that our original wording was too broad. In our study, these candidates were tested as 3WJ-4×Bro-tagged fusion transcripts in a defined experimental framework, with an initial transient screening in *N. benthamiana* followed by native-promoter graft validation in *Arabidopsis* only for the positive candidates. We have therefore revised the relevant text throughout the manuscript to clarify this point. Specifically, we now state that only three of the 100 RNA-seq-predicted candidates showed reproducible long-distance mobility under our assay conditions, rather than implying that the remaining candidates are universally non-mobile in different biological contexts (lines123-128).

We also appreciate the reviewer’s point regarding fusion-based assays. the protein-based tagging strategies have been reported to perturb RNA conformation, localization, or trafficking (Garcia and Parker, 2015, Christensen et al., 2010, Heinrich et al., 2017). Compared with protein-based tags, the 3WJ-4×Bro tag sequences are shorter and previous studies have shown that ligands do not affect the transcription and translation of target mRNA(Bai et al., 2020, Liu et al., 2025). In the present study, we also tested if 3WJ-4×Bro could perturb performance of a well-characterized mobile RNA *TCTP1*. In root cells, the 3WJ-4×Bro signal showed strong agreement with independent FISH-based detection, supporting its utility as a comparatively low-perturbation readout for long-distance RNA movement in our assay system.
In addition, as suggested, we have added a new supplementary table listing all tested candidate transcripts, together with their gene identifiers, the constructs used, and the outcomes of the screening assays. Please see Table EV1 in revised supplementary information file

- Figure 1B confocal images region 1,2 and 3: The majority of the signal appears to be present in the apoplast and not within the cells (especially in infiltrated leaves, region1). Please explain and show higher resolution/quality images allowing to see the distribution of the fluorescent signal within the cells. Especially as one would expect that mRNA is detectable in the cytosol after expression in transfected cells (region 1) and after expected symplastic delivery (regions 2 and 3).

Response：

Thank you for this important comment. For region 1, we note that in plant epidermal cells the large central vacuole occupies most of the cell volume and compresses the cytoplasm into a very thin cortical layer adjacent to the plasma membrane. As a result, cytoplasmic RNA-associated fluorescence can appear as a peripheral signal and may be difficult to distinguish from the cell periphery or apoplastic space when imaged at limited resolution. We agree that this was not sufficiently clear in the original figure.
To address this concern, we have replaced the original images in Figure 1B with higher-resolution images. In addition, we performed Propidium iodide (PI) staining to indicate cell boundaries, which allow clearer visualization of the relationship between the 3WJ-4×Bro signal and the cellular boundaries, and support an intracellular rather than extracellular interpretation of the fluorescence pattern in regions 1–3.

- Control experiment missing in the FISH experiment shown in Figure 1 E. Here no mock infiltrated plant tissue (w/o TCTP) is shown confirming the specificity of the use FISH probe. This has to be implemented.

Response：

We also appreciate the reviewer's point regarding the FISH experiment in Figure 1E. We agree that a negative control is necessary to demonstrate probe specificity. We have added a mock infiltrated plant tissue control to the FISH analysis. In this control, no specific Cy3 signal was detected under the same imaging conditions, supporting the specificity of the probe used to detect the *TCTP1* transcript. The corresponding control images and the associated description have now been included in the revised Fig EV1D.

Extent of transport is not shown in grafted plants. *TCTP1* is reported to move from transgenic shoots mainly to root tips and remains in close proximity of the phloem vasculature (see Yang et al). Do the authors observe a similar distribution of their fusion construct? Thus, an image showing the root tip and the different presence in different root regions should be shown. Also, the presence of tagged *TCTP1* transcript in the grafted root should be evaluated by RT-PCR assays.

Response：

Thank you for raising this important point. We agree that the original Figure 1D, which showed fluorescence only in root phloem 1.5 cm below the graft junction, did not sufficiently illustrate the spatial extent of *TCTP1* transport within the grafted root system.

To address this, we performed a more detailed analysis of grafted *Arabidopsis* roots using transgenic scions expressing *TCTP1*-3WJ-4×Bro grafted onto wild-type rootstocks. At 14 days after grafting, recipient roots were imaged under identical confocal settings across the maturation zone, elongation zone, meristematic zone, and root cap. The revised Fig 1D show that the transported *TCTP1*-3WJ-4×Bro signal appears predominantly in vasculature tissue of root. Notably, the signal is most strongly enriched in the root tip. This distribution pattern is consistent with the previously reported localization pattern of mobile *TCTP1*.

In addition, *TCTP1* transcript in the grafted root was evaluated by RT-PCR assays, which is shown in Fig. EV1B in our revised supplementary file.

- In general, the authors suggest that their identified GME2 motif outperforms a TLS motif-based system. However, they do not show any comparative data concerning mobility in grafted plants and whether the GME2 motif mediates mobility from roots to above ground tissues / flowers like the TLS motif does.

Response：

Thank you for this important comment. We agree that our original wording overstated the scope of the comparison with TLS-based systems.

In this study, we just compared the transport efficiency and mobility rate between 2×GME2 and the $tRNA^{Met}$-based TLS module by *Nicotiana benthamiana* transient assay and transgenic grafting assay. Under equivalent 5×mCherry cargos, 2×GME2 showed higher transport efficiency and mobility rate than the representative TLS module. we have revised the

manuscript to make this limitation explicit (lines208-212).

Our grafting experiments in *Arabidopsis* do show that 2×GME2 can mediate bidirectional long-distance transport across graft junctions: from rootstock to recipient leaves in the *AtMYB49* experiments, and from scion to recipient roots in the *AtHMA3* and *AtPCR2* experiments. We therefore conclude that 2×GME2 functions effectively in grafted plants for both shootward and rootward delivery.

However, we did not perform a side-by-side comparison between 2×GME2 and TLS in mediating mobility from roots to flowers.

- As with tagged *TCTP1* transcript: Can a GME2 delivered tagged transcript be detected in the root tips and in the vasculature of grafted plants? Such images should be shown and discussed.

Response：

Thank you for this helpful suggestion. To further examine the spatial distribution of the GME2-tagged transcript in grafted plants, we performed additional imaging using the same detection strategy applied to the tagged *TCTP1* transcript. *Arabidopsis* plants expressing the GME2-3WJ-4×Bro transcript were used as scions and grafted onto wild-type rootstocks. At 14 days after grafting, recipient roots were imaged under identical confocal settings across the maturation zone, elongation zone, meristematic zone, and root cap.
The revised data show that the GME2-3WJ-4×Bro signal is detectable in grafted recipient roots, including the root tip region. In the maturation and elongation zones, the signal was predominantly associated with the phloem region, whereas in the meristematic zone and root cap it was mainly detected in peripheral root tissues. These new images support the conclusion that GME2-tagged transcripts can be detected in both vascular tissues and root tip-associated regions of grafted plants.
Representative images have now been included in the revised manuscript (Fig EV7), and the corresponding description and discussion have been added to the revised text Lines294-297.

-Experiment with GFP-tagged mobile *MYB49*-GME2 and distribution of the GFP tagged mobile construct in the expressing and recipient roots/shoots. One would expect that the MYB transcription factor is also found in the nucleus and is not evenly distributed in the cytosol of the cells as shown in the figure 6D confocal images. Is this a consistent pattern based on the fusion construct? Again, here one should show more and higher quality images of different root cells / regions showing the distribution of the mobile construct in the roots. Also, one should confirm the mobility of the transcript by RT -PCR assays. Also, as MYP49 is shown to change the expression of known target genes one should use quantitative RT-PCR assays to confirm that the mobile transcript results in a change of expression in roots confirming it is functional in recipient tissues.

Response：

Thank you for this important comment. We apologize for a figure assembly error in the original submission. Specifically, the *AtMYB49*-GFP-2×GME2 image in Figure 6D was inadvertently interchanged with the corresponding *AtMYB49*-3WJ-4×Bro-2×GME2 image in Figure 6B. This error has now been corrected in the revised manuscript.

In the corrected Figure 6D, the GFP signal of *AtMYB49*-GFP-2×GME2 is predominantly nuclear, which is consistent with the expected subcellular localization of a MYB transcription factor. We have also replaced the original panel with higher-resolution confocal images to more clearly show the fluorescence distribution in the relevant donor and recipient tissues.

To directly confirm transcript mobility, we performed RT–PCR assays on RNA isolated from the corresponding recipient tissues. These analyses confirmed the presence of the *AtMYB49*-GFP-2×GME2 transcript in WT recipient leaves, as well as the corresponding *AtHMA3*-GFP-2×GME2 and *AtPCR2*-GFP-2×GME2 transcripts in WT recipient roots, providing molecular support for the imaging results (Fig. 6B and 6F, Fig. EV6E).

In addition, to test whether the delivered *AtMYB49* transcript is functionally active in recipient tissues, we performed quantitative RT–PCR analysis of known *AtMYB49* target genes (*MYB41* and *KCS2*) in WT recipient leaves. As expected, the expression of *MYB41* and *KCS2* significantly increased in leaves of WT / *AtMYB49*-2×GME2 compared to that of WT / WT grafts, supporting that the delivered *AtMYB49* transcript is not only mobile and translatable, but also functionally active in recipient tissues (Fig. EV6A).

- Figure 5G and efficiency of transport compared to the TLS motif. The authors write in the results that "Importantly, the distal fluorescence detection rate with 2×GME2 increased by ~24-fold in region 2 and ~8-fold in region 3 compared with the TLS system (Figure 5G)." However, figure 5 D only shows the fluorescence intensity in region 2 and 3 of 5xCherry-TLS (according to the legend). Thus, these data are missing / not presented. Also, no image is provided allowing to evaluate the expression/ distribution of mCherry fluorescence in the infiltrated plants. As the authors state in the text mCherry is expressed in the recipient cells. Thus, it should be possible to detect it under a CLSM and present such images.

Response：

Thank you for this important comment. We agree that the original presentation of Figure 5 did not make the comparison between the 2×GME2 and TLS systems sufficiently clear.

To address this, we have revised Figure 5F and 5G to explicitly present the direct comparison between 2×GME2 and TLS using the same 5×mCherry cargo. The revised text read: “In both the upward direction (region 1 to region 2) and the downward direction (region 1 to region 3), 2×GME2 showed a higher mobility rate than TLS (Fig. 5F, Fig. EV5C), indicating more efficient long-distance movement of the same cargo. Consistent with this result, 2×GME2 also

exhibited a higher transport velocity than TLS in both directions (Fig. 5G). Together, these data show that, under our assay conditions, 2×GME2 mediates both more efficient and faster long-distance transport of the same RNA cargo than the representative TLS module.”

In addition, as suggested, we have added representative images showing the mCherry fluorescence distribution in the relevant tissues, and these images are now included in Figure 5C and 5D.

- The discussion text makes strong statements such as"... this work establishes a modular, sequence-defined RNA mobility system that directly addresses the lack of efficient, broadly applicable, non-viral carriers for graft-mediated trait improvement38,39." However, this statement is underlined with "strange" references which are not really relevant for this work. One (Yang et al) shows a sequence-defined RNA mobility /editing system. Here do make such a statement one has to compare the known editing efficiency of the existing TLS based editing system with a GME2 based system in the context of Cas9 - which was not done. This would be in particular important as it was not shown with the presented GME2 motif that it can mediate delivery to the apical meristematic tissues forming the offspring. In similar line, the other manuscript cited is a review focusing on m6A modifications and does not addressing RNA mobility.

Response：

Thank you for this comment. We revised the Discussion to narrow the scope of this statement The revised text now describes 2×GME2 as a modular, sequence-defined RNA mobility element that expands the toolkit for non-viral graft-mediated RNA delivery, rather than implying validated superiority for genome-editing applications. In addition, we replaced the references that were not directly appropriate for supporting the original statement. These changes are marked in red in the revised Discussion section (lines 337–340).

- In a similar line, in the following paragraph the authors imply that the GME2 mobility motif may be better than the TLS system without any evidence and claim that "TLS-based graft-mobile editing systems [.....] require expression of bulky CRISPR components in rootstocks and often depend on strong selection to retrieve rare editing events". This claim is no substantiated in their presented results as e.g. no movement mediated by the GME2 to flowers or tissue forming offspring was confirmed, no Cas9 fusion was tested, ...etc.. Thus, these statements should be adopted/ rephrased / changed or deleted accordingly. Again, the authors claim referring to the TLS system that "By contrast, 2×GME2 is a short, sequence-defined cassette..." This claim is not supported by data as the TLS system has a similar length (of approx. 40-35 bases) and has a short, defined sequence (between 70 to 40 bases) which was shown to mediate movement of non-mobile, long and short transcripts. Here the authors over emphasize their finding of an interesting new motif which might be better suitable or not. In general, such statements appear not helpful and do not underscore their interesting findings and data, especially if they are not substantiated by proper experiments.

Response：

Thank you for this comment. We revised this paragraph to remove unsupported comparisons with TLS-based editing systems, including statements related to Cas9 delivery, editing efficiency, meristem targeting, and progeny-related outcomes, as these were not tested in this study. The revised text now describes 2 × GME2 more neutrally as a compact, sequence-defined, non-viral mobility element that supports graft-mediated RNA delivery in the systems tested here, while TLS- and virus-based platforms are discussed as complementary approaches in the revised Discussion section (Lines 249-251).

Minor suggestions/corrections:

Introduction:
Statement "...proof-of-concept studies have demonstrated grafting-induced, transgene-free genome editing using TLS or virus-based systems, these approaches still suffer from low editing efficiency, restricted cargo capacity, and biosafety concerns associated with viral vectors.". Should be rephrased as "restricted cargo capacity, and biosafety concerns" only apply to viral-based systems.

Response：

Thank you for this comment. We revised this sentence to separate the limitations of TLS-based and virus-based systems more precisely. Specifically, we removed the wording that could be interpreted as attributing restricted cargo capacity and biosafety concerns to TLS-based systems. The revised text now states these limitations only in the context of viral vectors, while the sentence has been rephrased more generally for TLS-based approaches. This change has been made in the revised Introduction (Lines 53-59).

The statement that "Up to now, only three mobile mRNA motifs...." Were found is not right. Others were identified e.g. in AtHSC70.1 (the SVR in the 3' ORF) and in TCTP1 (in the 5' ORF). Also, the statement that m5C and m6A are motifs seems to be an oversimplification of the reported studies. For example, HSC70.1 and TCTP1 mobility appear to depend on m5C modifications in their respective motif regions but can act a mobility motifs of as shown with fusion constructs.

Response：

Thank you for this comment. We revised this part of the Introduction to make the description of previously reported mobile RNA determinants more accurate. Specifically, we removed the statement that “only three mobile RNA motifs” have been identified. We now acknowledge additional reported mobility determinants, including those associated with *TCTP1* and HSC70.1, and we revised the text to distinguish sequence/cis-acting mobility elements from mobility-associated RNA modifications. Accordingly, m5C and m6A are no longer described

as “motifs” but as RNA modifications linked to mRNA mobility. These changes have been incorporated into the revised Introduction section (Lines 49-52).

Correct the citation in "... as could support non-transgenic precision breeding via graft-mediated RNA delivery33." Cite the first report/suggestions on graft mediated RNA delivery and precision breeding (e.g. Zhang et al 2016 Plant Cell and/or Yang et al 2023 Nature Biotech.)

Response：

Thank you for this comment. We corrected the citation for this statement and cited Zhang et al. (2016, Plant Cell) for the early demonstration that sequence-defined RNA elements can confer systemic mRNA transport, and Yang et al. (2023, Nature Biotechnology) for graft-mediated production of transgene-free edited progeny (Lines 73).

Correct the citation for "CK120 " (Li, X. J. et al. m5C and m6A modifications regulate the mobility of pumpkin CHOLINE KINASE 1 mRNA under chilling stress. Plant Physiology 197, 1-16, (2024).) to 25 (Zhang, W. et al. tRNA-Related Sequences Trigger Systemic mRNA Transport in Plants.The Plant Cell 28, 1237-1249, (2016)) as Arabidopsis CK1 mobility was first reported by Thieme, C. J. et al. (Endogenous Arabidopsis messenger RNAs transported to distant tissues. Nature Plants 1, 1-9, (2015)) and by Zhang W et al. 2016. This is especially important as the authors use AtCK1 in their submitted study to confirm its mobility.

Response：

Thank you for this comment. We corrected the citation for CK1 so that the manuscript now refers to the earlier studies reporting its mobility, rather than to the later paper addressing m5C/m6A-dependent regulation of pumpkin *CK1* (Lines 43).

Correct the citation for the statement "...subsequent re-analyses and experimental validation have revealed high false-positive rates and have indicated that only a small fraction of predicted candidates are truly mobile over long distances29 " The correct citation should be: "Paajanen et al. Re-analysis of mobile mRNA datasets raises questions about the extent of long-distance mRNA communication. Nat Plants. 2025 May;11(5):977-984. doi: 10.1038/s41477-025-01979-x. Epub 2025 Apr 16. PMID: 40240650; PMCID: PMC12095074." Note, that this re-analysis only refers to the SNP-based mobility datasets and that they appear to have a high error rate (> 50%). Other datasets including e.g. transcripts moving from Arabidopsis to Cuscuta are not affected by such a SNP calling error based on RNAseq data.

Response：

Thank you for this comment. we now cite Paajanen et al. (Nature Plants, 2025) for the statement regarding the high false-positive rate in short-read RNA-seq/SNP-based

graft-mobile mRNA datasets. We also revised the sentence to avoid generalizing this conclusion to all types of mobile RNA datasets, as the Paajanen et al. study specifically addressed graft-based RNA-seq datasets of this type. These changes have been incorporated into the revised Introduction. (Lines 58-62).

The statement "Using this system, we systematically re-evaluate 100 RNA-Seq-predicted mobile mRNAs and identify only three bona fide long-distance mobile transcripts (CAT318, CK120, and GAI17)," This is not right as they also confirmed AtTCTP1 which was reported to be mobile in several manuscripts. Furthermore (see also major comments above), there is not list of the "100" tested transcripts presented in the results (e.g. as supplement data with references) supposedly not to be mobile. This has to be implemented and corrected.

Response：

Thank you for this comment. We corrected this statement to distinguish the 100 RNA-seq-predicted candidates from the previously reported mobile transcript TCTP1, which was used in this study as a positive control for validating the 3WJ-4×Bro imaging system rather than as one of the 100 screened candidates. (Lines 120-123)
Accordingly, we revised the text to state more precisely that, among the 100 RNA-seq-predicted candidates tested in our assay, only three showed reproducible long-distance mobility under our experimental conditions. In addition, as suggested, we have added a new supplementary table listing all 100 tested transcripts, together with their gene identifiers, constructs, and screening outcomes. Please see Table EV1 in the revised Supplementary Information file.

Referee #2:

In this manuscript, Tao et al. applied mRNA imaging system to track mobile mRNA in plants and elucidated the systemic movement of CAT3, CK1, and GAI mRNAs in planta through the application of a fluorescence-aptamer-based RNA imaging tool. This study identify cis-elements governing the systemic movement of GAI mRNA and develop a new RNA delivery system, referred to as the 2xGME2 system. By employing the 2xGME2 system in conjunction with grafting techniques, the authors demonstrated that the 2xGME2 element fused-AtMYB49, AtHMA3 and AtPCR2 mRNAs can be transported from scion to rootstock or from rootstock to scion. In these grafts, tolerance to abiotic stresses were increased. Overall, it is very interesting study.
Most experiments are well designed and thoroughly documented. I provide minor comments below.

Regarding Fig. 1, it would be preferable to explain the intracellular localization of mRNA.

Response：

Thank you for this helpful suggestion. We have revised Figure 1 and the associated text to better explain the intracellular localization of the RNA signal. Specifically, we now clarify that in plant cells the large central vacuole compresses the cytoplasm into a thin cortical layer near the cell periphery, so the fluorescence signal from 3WJ-4×Bro-tagged mRNAs is expected to appear mainly in the peripheral cytoplasmic region rather than uniformly throughout the cell interior. To make this localization clearer, we replaced the original images with higher-resolution confocal images and added PI staining to mark the cell wall and define cell boundaries. These revisions are now included in the revised Figure 1.

In line 125, Thieme et al. identified 918 transcripts as mobile mRNAs, and at least 222 of these were shown to move bidirectionally between root and shoot tissues. How were these narrowed down to 100 candidates? Additionally, the authors should provide the list of the 100 candidate mobile mRNAs.

Response：

Thank you for this comment. We revised the manuscript to clarify how the 100 candidate transcripts were selected from the larger set reported by Thieme et al. Because imaging-based validation of all 918 reported candidates was not practically feasible, we selected a random subset of 100 transcripts from this dataset for first-pass screening using the 3WJ-4×Bro imaging system. We have now made this sampling strategy explicit in the revised manuscript. In addition, we added a supplementary table listing all 100 tested candidate mobile mRNAs, together with their gene identifiers and screening outcomes in our assay (Table EV1).

Please show the nucleotide sequences of GME1 and GME2. Do these RNA motifs form secondary structures? In addition, are the GME motifs found in other mobile mRNA candidates?

Response：

Thank you for this helpful comment. We have revised the manuscript to provide the nucleotide sequences of GME1 and GME2. In addition, we performed in silico secondary-structure prediction for both motifs, and the predicted RNA secondary structures are now included in Figure EV8. Through sequence alignment, we did not find the GME-like motif in other mobile mRNA candidates.

Regarding with Fig. 5A, is the velocity of mRNA dependent on the number of GME copies? If so, does this dependence apply when there are three or more copies?

Response：

Thank you for this question. In the original manuscript, we highlighted the comparison between one copy and two tandem copies of the GME element, because the 2×GME construct showed improved long-distance transport relative to the single-copy version.

To further address the reviewer's question, we have now included additional data for constructs carrying three, four, or five copies of the GME element fused to the mCherry-3WJ-4×Bro transcript. Under our experimental conditions, these 3×–5× constructs did not show significant differences in mobility efficiency from one another. Thus, our data support an improvement from one to two copies, but do not indicate a continued increase in transport efficiency with further increases in copy number beyond this range. These additional results have now been included in the revised Figure EV5B, and the corresponding text has been updated accordingly (Lines 195-197).

Regarding with Fig.5B and S5, how was the time course of distal fluorescence detection frequency quantified? The authors should describe the method in detail.

Response：

Thank you for this comment. We have changed statement "distal fluorescence detection frequency" as "mobility rate" according the related reports ((Zhang et al., 2025)). The methodological details on quantification of the time course of mobility rate were added Figure 5 legend and Supplementary Fig.EV5 legend. Specifically, we now state that, for each construct and each time point, 20 independently inoculated plants were examined under the same confocal imaging settings at the defined distal regions. A plant was scored as fluorescence-positive when a clear 3WJ-4×Bro signal was detected in the designated distal region. The distal fluorescence detection frequency was then calculated as the percentage of fluorescence-positive plants among the total plants examined at each time point.

Regarding Lines 261-262, it is unclear what is being compared. Figure 5G does not directly correspond to the explanatory text.

Response：

Thank you for this comment. We revised Figure 5F–G and the corresponding Results text to make the comparison explicit. The revised figures now directly compare 2×GME2 and TLS using the same 5×mCherry cargo in both the upward and downward directions, with Fig. 5F showing the mobility rate and Fig. 5G showing the transport velocity. The figure legends and text have also been revised accordingly (Lines 642-646).

Regarding AtMYB49, AtHMA3, and AtPCR2 in lines 284-285, it would be preferable to explain what genes they encode, in which tissues they function, and their roles. Otherwise, the rationale for the rootstock and scion combinations in the grafting experiments described in Lines 303-315 is unclear.

The upward-delivery section uses *AtMYB49* as the cargo to illustrate transport from the rootstock to the scion and the resulting improvement in salt tolerance. Please see Results text, lines214–217. The downward-delivery section uses *AtHMA3* and *AtPCR2* as cargos to illustrate transport from the scion to the rootstock and the resulting improvement in Cd tolerance and Zn tolerance, respectively. Please see Results text, lines231–232.

Regarding with Fig.6D, although MYB49 is a transcription factor, the MYB49-GFP signal does not appear to localize to the nucleus. What could be the reason why the signal does not appear in the nucleus? Further analysis and explanation of the intracellular localization are necessary.

Response：

Thank you for this important comment. We identified a figure assembly error in the original submission: the *AtMYB49*-GFP-2×GME2 panel in Figure 6D had been inadvertently interchanged with the corresponding *AtMYB49*-3WJ-4×Bro-2×GME2 panel in Figure 6B. This error has now been corrected in the revised manuscript.
In the corrected Figure 6D, the GFP signal of AtMYB49-GFP-2×GME2 is predominantly nuclear, consistent with the expected intracellular localization of a MYB transcription factor. We have also replaced the original panel with higher-resolution confocal images to more clearly show the intracellular distribution of the signal.

The explanation in Lines 303-309 needs to include an explanation of the results of transgenic homografts shown in Fig. 6E-J. There is no explanation of what plants the labels AtMYB49, AtHMA3, and AtPCR2 in Fig. 6E-J refer to.

Response：

Thank you for this comment. To improve the clarity of Figure 6, we reorganized the figure according to the direction of graft-mediated mRNA delivery and the corresponding trait-improvement assay.
Specifically, the revised figure is now divided into two sections. The upward-delivery section uses *AtMYB49* as the cargo to illustrate transport from the rootstock to the scion and the resulting improvement in salt tolerance. Please see Results text, lines214–217. The downward-delivery section uses *AtHMA3* and *AtPCR2* as cargos to illustrate transport from the scion to the rootstock and the resulting improvement in Cd tolerance and Zn tolerance, respectively. Please see Results text, lines231–232.

We also revised the panel organization, labels, and figure legend so that the meaning of *AtMYB49*, *AtHMA3*, and *AtPCR2* is now explicitly defined in the context of the corresponding graft combinations and stress assays, shown in revised Figure 6 and Figure EV6)

The unit for the NaCl concentration in Fig. 6E is incorrect and needs to be corrected.

Response：

Thank you for pointing this out. We have now corrected the unit in the revised figure and carefully checked the corresponding figure legend and main text to ensure consistency.

Referee #3:

Tao et al use an aptamer-based RNA imaging system to identify novel mobilizing sequences, characterize the dynamics of mobility, and deliver non-mobile abiotic stress-responsive RNAs that enhance abiotic stress tolerance upon delivery. The paper adds substantial new insights to the field of RNA mobility. One significant point that I would like to raise is that the authors should specify which cell layers are being imaged in all of their experiments, particularly with regard to root imaging. It looks like the epidermal layer is being imaged, which is an unexpected destination for vascular-mobile RNAs. For most of the experiments, it also looks like the authors imaged halfway down the root, rather than at the root tip, where I would expect RNAs to exit the phloem transport stream. Cross sections through roots at high, mid, and root tip points would provide a more informative look at how RNAs are moving from the vasculature into the cortex and potentially epidermal tissue layers.

Response：

Thank you for this important comment. We revised the manuscript to clarify the tissue layers imaged in our fluorescence analyses, particularly for the root samples.
we have added longitudinal optical sections of intact roots including the maturation zone, elongation zone, meristematic zone, and root cap. These images indicate that, in the maturation and elongation zones, the signal is predominantly associated with the vascular region, whereas in the meristematic and root-cap regions it is mainly detected in peripheral cell layers.
To further improve interpretability, we also included higher-resolution images and PI staining to mark cell boundaries where appropriate. We agree that transverse root sections would be informative for resolving the radial route of RNA unloading in greater detail; however, this was not systematically addressed in the present study. We therefore revised the text to define the imaged tissues more clearly and to avoid overinterpretation of the precise cell-to-cell route of RNA movement.

I've listed some additional points below:

Line 31: should be revised to "the empirical selection of rootstock scion combinations"

Response：

Thank you for this suggestion. We have revised Line 31 accordingly. The phrase has been changed to "the empirical selection of rootstock-scion combinations" in the revised manuscript, marked by red(Lines 32).

Line 66: The data from the screen of 100 candidates should be included as supplemental data, or this experiment should not be included in the paper.

Response：

Thank you for this comment. We have added a supplementary table listing all 100 tested candidate mobile mRNAs, together with their gene identifiers, constructs, and screening outcomes in our assay please see Table EV1.

lines 91-94: RT-PCR is an insufficient test to exclude the possibility of contaminating Agrobacterium because the Agro infection is a continuous process, and the RT-PCR is only informative for the time point when samples are collected. The authors produced stable transgenic lines for all essential pieces of evidence in this study, so the transient assays are okay for an initial screen.

Response：

Thank you for this important and insightful comment. Thank you for this important comment. We revised the text to avoid overstating the ability of the transient RT–PCR/16S assay to fully exclude Agrobacterium persistence or later spread (Lines100-102).

An interesting follow-up computational experiment for the authors to consider: It would be useful for the authors to dig deeper into the evolutionary significance of GME1 and GME2. Are these sequences conserved in other flowering plant species? Do these sequences contain conserved motifs that are found in other mobile RNAs?

Response：

Thank you for this helpful suggestion. We agree that the evolutionary conservation of GME1 and GME2, and their possible relationship to mobility-associated features in other mobile RNAs, is an interesting question.
However, this was not systematically addressed in the present study. Our current work focuses on the experimental identification and functional characterization of these motifs in the systems tested here, and we have therefore avoided broader claims regarding their evolutionary conservation or general occurrence in other mobile transcripts. We regard cross-species conservation analysis and comparative motif mining as important directions for future study.

Figures:

Figure 1: The localization of the fluorescence in Fig 1B looks extracellular. Higher resolution images for this experiment would be useful to determine the localization of the mobile RNA. In addition, a supplemental figure showing negative controls under the exact same imaging conditions is necessary to separate signal from autofluorescence. The co-localization experiments in Figure 1 provide nice substantiating evidence for the movement of 3WJ-4xBro

Response：

Thank you for this helpful comment. We revised Figure 1B to better resolve the localization of the fluorescence signal. Specifically, we replaced the original images with higher-resolution confocal images and included PI staining to mark the cell wall and define cell boundaries more clearly. These revised images support an intracellular, peripheral cytoplasmic localization pattern rather than extracellular accumulation. In addition, as suggested, we added a new supplementary figure showing the relevant negative controls acquired under the same imaging conditions, which allows clearer distinction between true fluorescence signal and tissue autofluorescence.

Lines 143-144: The authors should reference previous work characterizing GAI movement in this statement. For example, "Congruent with previous research on GAI mRNA mobility, these data demonstrate that GAI is a highly efficient long distance...."

Response：

Thank you for this comment. We revised this statement to acknowledge previous work reporting GAI mRNA mobility. Specifically, the revised text now places our result in the context of earlier studies and states that our data are consistent with previous reports of GAI mobility, while further showing that GAI exhibits particularly high long-distance transport efficiency in our assay system. The relevant reference has now been added at this position in the revised manuscript (lines 138-140).

Line 205/Figure 4: The authors should describe how they identified phloem cells in their imaging datasets.

Response：

Thank you for this comment. We revised the manuscript to clarify how the phloem region was identified in the imaging data shown in Figure 4. Specifically, the root images were acquired as longitudinal optical sections of intact *Arabidopsis* roots. In the revised figure and text, we now indicate that the phloem region was identified based on its characteristic anatomical position within the root stele, and this interpretation was further aided by PI staining, which was used to mark cell walls and define tissue organization more clearly. We therefore revised the wording to refer more precisely to the phloem region rather than over-interpreting individual cells at single-cell resolution. These changes have been incorporated into the

revised Figure 4 legend and corresponding Results

For the experiments in Figure 5: In order to make comparative statements about the mobility of different sequences, it's necessary to quantify the source expression of the different constructs. Different levels of transient expression in the source tissue could significantly impact transport dynamics and produce misleading conclusions regarding the efficiency of different mobilizing sequences.

Response：

Thank you for this important comment. We agree that variation in transient expression at the source tissue could influence apparent transport performance and should be considered when comparing different constructs.

In our experimental design, several measures were taken to minimize this effect. For each plant, only one leaf was used for agroinfiltration. All constructs were infiltrated using the same bacterial culture density and the same inoculation volume. In addition, for each construct and each time point, we examined 20 independently inoculated plants, and all comparisons were performed using the same imaging workflow. Because the inoculated leaf had to be excised for imaging, we could not directly monitor the initial expression level of each construct in the source tissue in parallel.

To quantify transport velocity, we defined the relevant time interval as the time after inoculation at which the first fluorescence-positive plant was detected in the designated distal region among the 20 independently inoculated plants. Transport velocity was then calculated as the distance from the inoculation site to the distal region divided by this time interval. Each experiment was repeated three times, and the mean value was used to represent the transport velocity mediated by each construct.

We have now added these methodological details to the revised manuscript and, accordingly, describe the Figure 5 comparisons more cautiously as differences in transport performance under our assay conditions.

Methods:

It is important for the authors to provide a supplemental file including sequence information for all of the constructs used in this study. This is essential for external replicability, especially given that the results are linked to specific nucleotide sequences.

Response：

Thank you for this important suggestion. The sequence information for all of the constructs has been deposited in the BioStudies database record (https://www.ebi.ac.uk/biostudies/studies/S-BSST2877?key=0ca6e59d-88dd-433f-b392-062196ed4bf6).

The authors need to include ecotype/genotype information in the materials and methods

section.

Response：

Thank you for this important comment. we have revised the manuscript to include the relevant Arabidopsis ecotype/genotype information in the Materials and Methods (Lines317-319)..

Please provide more details regarding the Arabidopsis micro grafting protocol.

Response：

Thank you for this helpful suggestion. we have expanded the Materials and Methods section in the revised manuscript to provide a more detailed description of the micrografting procedure (Lines365–374).

References

Bai J, Luo Y, Wang X, Li S, Luo M, Yin M, Zuo Y, Li G, Yao J, Yang H et al (2020) A protein-independent fluorescent RNA aptamer reporter system for plant genetic engineering. Nat Commun, 11, 1–14.

Christensen NM, Oparka KJ, Tilsner J (2010) Advances in imaging RNA in plants. Trends in Plant Science, 15, 196–203.

Garcia JF, Parker R (2015) MS2 coat proteins bound to yeast mRNAs block 5' to 3' degradation and trap mRNA decay products: implications for the localization of mRNAs by MS2-MCP system. RNA, 21:1393-5

Heinrich S, Sidler CL, Azzalin CM, Weis K (2017) Stem-loop RNA labeling can affect nuclear and cytoplasmic mRNA processing. RNA, 23: 134–141.

Liu S, Bai J, Zhan B, Yao J, Zhang J, Yi J, Dong M, Li Q, Shen Y, Chen Y et al (2025) Development of an RNA aptamer-assisted CRISPR/Cas9 system for efficiently generating and isolating Cas9-free mutants in plant. PLoS Genet 21: e1011931.

Zhang M, Liu W, Wang C, Lin S, Chen Y, Cui H, Xiang C, Ma Y, Li X, Lu Y et al (2025) Root-to-shoot mobile mRNA CmoKARI1 promotes JA-Ile biosynthesis to confer chilling tolerance in grafted cucumbers. Nat Commun 16: 7782

Dear Dr. Zhao,

Thank you for the submission of your revised manuscript. We have now received the enclosed reports from the referees and I am happy to say that all support its publication now. Referees 1 and 3 still have some more suggestions that I would like you to address and incorporate before we can proceed with the official acceptance of your manuscript. Referee 1 point 2 can be discussed in the ms text.

Also a few editorial requests will need to be addressed:

- Please correct the conflict of interest subheading to "Disclosure and Competing Interests Statement".

- The author credits need to be removed from the ms file. All credits need to be entered during online ms submission.

- The manuscript sections should be in the following order: Title page - Abstract - Introduction - Results - Discussion - Methods - Data Availability - Acknowledgments - Disclosure and Competing Interests Statement - References - Figure Legends - (Main Tables with legends if applicable) - Expanded View Figure Legends.

- Materials and Methods to be labeled as 'Methods'

- The Reagents and Tools Table need to be removed from the ms file. It only needs to be uploaded as a separate file.

- Our routine image analyses detected a possible image reuse in Figure 5D. Can you please explain and clarify?

* Figure Legends - Comments *

- Please note that the legends for figures 2, EV3 is not provided in the sequential manner. This needs to be rectified.
- Please note that the exact p values are not provided in the legend of figure 4C. Please provide exact values as reasonable.

I would like to suggest some minor changes to the abstract that needs to be written in present tense. Do you agree with this:

Systemic RNA movement offers a potential route for non-transgenic trait improvement by grafting, but progress has been limited by the lack of reliable tools to identify mobile RNA elements and by the limited cargo capacity of existing delivery systems. Here, we report a fluorescence-aptamer-based live-imaging platform for screening mobile RNAs and mapping mobility elements. Live imaging of 100 RNA-Seq-predicted candidates identifies three bona fide mobile mRNAs (CAT3, CK1, and GAI), with GAI mRNA exhibiting the highest bidirectional mobility. Truncation analysis defines two independent 30-nt cis-elements, GME1 in the coding region and GME2 in the 3′UTR, with GME2 mediating higher speed and efficiency (efficiency of what?). A tandem 2×GME2 cassette in the 3′UTR functions as a high-capacity RNA delivery module and, in our transient assay, outperforms the representative TLS (what is TLS?) construct tested here. Using 2×GME2, we deliver three otherwise non-mobile mRNAs across graft junctions and detect associated molecular or phenotypic effects in recipient tissues. Together, these findings establish a live-imaging-guided pipeline for mobile RNA discovery and identify 2×GME2 as a modular non-viral RNA delivery element for graft-mediated trait modification.

EMBO press papers are accompanied online by A) a short (1-2 sentences) summary of the findings and their significance, B) 2-3 bullet points highlighting key results and C) a synopsis image that is exactly 550 pixels wide and 300 pixels high. The synopsis image should provide a sketch of the major findings, like a graphical abstract. Please note that text needs to be readable at the final size. Please send us this information along with the final manuscript.

I look forward to seeing a final version of your manuscript as soon as possible.

Best regards,
Esther

Esther Schnapp, PhD
Senior Editor
EMBO reports

Referee #1:

In the revised / resubmitted manuscript titled " Developing a Mobile RNA Delivery System for Grafting Trait Improvement" by Tao et al. the authors represent a method to trace mobile mRNAs after agroinfiltration and in grafted plants using 3WJ-4.Bro tagged transcripts. and identified two new motifs that appear to mediate mRNA transport. The revised manuscript addressed all main points raised. However, there is still room for improvements.

Comments

-The text of the abstract should be rephrased: " ...limited cargo capacity of existing delivery systems". There are no grounds on which such a statement can be made. Please delete. Also, the statement:" Live imaging of 100 RNA Seq-predicted candidates identified only three bona fide mobile mRNAs ..." should be changed in the abstract or presented in the right context. The authors used a heterologous plant system (N. tabacum vs. Arabidopsis") to test their readout system.

-The phenotypic improvement experiments with transgenic mobile constructs.
Although it nicely shows that transgenic shoots or roots can change the growth behaviour of the grafted plants crucial controls are missing in form of transgenic expression of the same mRNAs lacking the mobility motif sequence. This is necessary to unequivocally confirm that the changed growth is depending on mobility and not the systemic transfer of other factors induced by the transgenes.

-Missing information that should be added:
The DNA sequence of the 3WJ-4xBro fused to 2×GME2 used in the study should be presented. Also it is not mentioned which TLS sequence was used for the comparison of mobility.

Referee #2:

Thank you for the revised version of the manuscript. I have carefully reviewed the authors' responses and the changes made. The revision adequately addresses my previous comments, and I have no further concerns.
I recommend that the manuscript be accepted in its current form.

Referee #3:

The revised manuscript by Tao et al. is much improved. We list a few additional minor revisions below:

Lines 16-18. "Live imaging of 100 RNASeq-predicted candidates identified only three bona fide mobile mRNAs (CAT3, CK1, and GAI), with GAI mRNA exhibiting the highest bidirectional mobility."
This statement is too strong, as previously pointed out by reviewer 1 in their first comment. It would be better to use an alternative term instead of "bona fide", for example, "demonstrated mobile mRNAs".

Lines 23-24. "Together, these findings establish a live-imaging-guided pipeline for mobile RNA discovery."
Discovery came from the original paper in which these 100 RNAs were identified; the term "validation" would be more appropriate.

Line 174. I think that hpi is hours post-infection, but you never clarify this in the text.

Line 175-187; 207-208. Is this really 'velocity' or 'mobile rate'? Single-molecule imaging would be required to make statements about velocity. It would be better to use another term, such as mobile rate or mobility estimate. Please define your parameters for this estimate within the main text, and not just in the methods.

Lines 206-207: Is it possible to estimate how base pairs correspond with kD? That would be interesting.

In Lines 209-211: The statement, "with full load delivery efficiency of approximately 35% (Fig. EV5B)" is unclear. Please define more clearly.

Lines 264-266: It would be more accurate to add the following qualifier to this statement - "Within this framework, only three of the 100 candidates tested here showed reproducible long-distance movement" + "in our experimental conditions". This added statement covers potential conditional requirements for movement that may not be consistent across experimental settings.

Dear editors and reviewers,

Thank you for your careful review and constructive suggestions regarding our manuscript entitled "Developing a Mobile RNA Delivery System for Grafting Trait Improvement" (previous manuscript ID: EMBOR-2025-63321V2). We have revised the manuscript in accordance with the comments and marked all the changes on our revised manuscript by red. Here are the point-by-point responses for the reviewers' comments. The responses are marked by blue.

Editorial comment

1. - Please correct the conflict of interest subheading to "Disclosure and Competing Interests Statement".

Response： Thank you for your comment. We have corrected the conflict of interest subheading to "Disclosure and Competing Interests Statement" in the revised manuscript (Line 419).

2. - The author credits need to be removed from the ms file. All credits need to be entered during online ms submission.

Response： Thank you for your comment. We have removed the author contribution/credit section from the manuscript file.

3.- The manuscript sections should be in the following order: Title page - Abstract - Introduction - Results - Discussion - Methods - Data Availability - Acknowledgments - Disclosure and Competing Interests Statement - References - Figure Legends - (Main Tables with legends if applicable) - Expanded View Figure Legends.

Response： Thank you for your comment. We have reorganized the manuscript sections according to the required order: Title page, Abstract, Introduction, Results, Discussion, Methods, Data Availability, Acknowledgments, Disclosure and Competing Interests Statement, References, Figure Legends, and Expanded View Figure Legend.

4.- Materials and Methods to be labeled as 'Methods'

Response： Thank you for your comment. We have changed the section heading from "Materials and Methods" to "Methods" in the revised manuscript (Line 243).

5.- The Reagents and Tools Table need to be removed from the ms file. It only needs to be uploaded as a separate file.

Response： Thank you for your comment. We have removed the Reagents and Tools Table from the manuscript file .

6.- Our routine image analyses detected a possible image reuse in Figure 5D. Can you please explain and clarify?

Thank you for bringing this issue to our attention. We carefully re-examined Figure 5D together with the corresponding original image files. We found that one image panel had been inadvertently duplicated during figure assembly. We apologize for this oversight. The duplicated panel has now been replaced with the correct representative image from the original dataset. We have also rechecked all other panels in Figure 5D and confirmed that they correspond to the appropriate experimental conditions. This correction does not affect the quantitative analyses or the conclusions drawn from this experiment.

Figure Legends - Comments *

7.- Please note that the legends for figures 2, EV3 is not provided in the sequential manner. This needs to be rectified.

Thank you for your comment. We have revised the legends for Figure 3 and Figure EV3 so that all panel descriptions are now presented in sequential order. The previously grouped panel descriptions have been separated into individual entries, and the statistical and replication information has been clarified at the end of each legend.

8.- Please note that the exact p values are not provided in the legend of figure 4C. Please provide exact values as reasonable.

Response： Thank you for your comment. We have revised the legend of Figure 4C to include the exact P value (Line 618) .

9.- I would like to suggest some minor changes to the abstract that needs to be written in present tense. Do you agree with this: Systemic RNA movement offers a potential route for non-transgenic trait improvement by grafting, but progress has been limited by the lack of reliable tools to identify mobile RNA elements and by the limited cargo capacity of existing delivery systems. Here, we report a fluorescence-aptamer-based live-imaging platform for screening mobile RNAs and mapping mobility elements. Live imaging of 100 RNA-Seq-predicted candidates identifies three bona fide mobile mRNAs (CAT3, CK1, and GAI), with GAI mRNA exhibiting the highest bidirectional mobility. Truncation analysis defines two independent 30-nt cis-elements, GME1 in the coding region and GME2 in the 3′UTR, with GME2 mediating higher speed and efficiency (efficiency of what?). A tandem 2×GME2 cassette in the 3′UTR functions as a high-capacity RNA delivery module and, in our transient assay, outperforms the representative TLS (what is TLS?) construct tested here. Using 2×GME2, we deliver three otherwise non-mobile mRNAs across graft junctions and detect associated molecular or phenotypic effects in recipient tissues. Together, these findings establish a live-imaging-guided pipeline for mobile RNA discovery and identify 2×GME2

as a modular non-viral RNA delivery element for graft-mediated trait modification.

Response: Thank you for your helpful suggestion. We agree that the Abstract should be written in the present tense and have revised it accordingly.

10.- EMBO press papers are accompanied online by A) a short (1-2 sentences) summary of the findings and their significance, B) 2-3 bullet points highlighting key results and C) a synopsis image that is exactly 550 pixels wide and 300 pixels high. The synopsis image should provide a sketch of the major findings, like a graphical abstract. Please note that text needs to be readable at the final size. Please send us this information along with the final manuscript.

Response: Thank you for your reminder. We have prepared the required online accompanying materials, including a short summary, key result bullet points, and a 550 × 300 px synopsis image. These files have been submitted together with the revised manuscript. The online accompanying materials have been uploaded as separate files

I look forward to seeing a final version of your manuscript as soon as possible.

Best regards,
Esther

Esther Schnapp, PhD
Senior Editor
EMBO reports

Referee #1:

In the revised / resubmitted manuscript titled " Developing a Mobile RNA Delivery System for Grafting Trait Improvement" by Tao et al. the authors represent a method to trace mobile mRNAs after agroinfiltration and in grafted plants using 3WJ-4.Bro tagged transcripts. and identified two new motifs that appear to mediate mRNA transport. The revised manuscript addressed all main points raised. However, there is still room for improvements.

Comments

1.- The text of the abstract should be rephrased: " ...limited cargo capacity of existing delivery systems". There are no grounds on which such a statement can be made. Please delete. Also, the statement:" Live imaging of 100 RNA Seq-predicted candidates identified only three bona fide

mobile mRNAs ..." should be changed in the abstract or presented in the right context. The authors used a heterologous plant system (N. tabacum vs. Arabidopsis") to test their readout system.

Response: Thank you for this helpful comment. We have revised the Abstract accordingly. We removed the statement regarding the "limited cargo capacity of existing delivery systems". We also revised the screening result to avoid overstatement. We removed "only" and "bona fide".

2. -The phenotypic improvement experiments with transgenic mobile constructs.

Although it nicely shows that transgenic shoots or roots can change the growth behaviour of the grafted plants crucial controls are missing in form of transgenic expression of the same mRNAs lacking the mobility motif sequence. This is necessary to unequivocally confirm that the changed growth is depending on mobility and not the systemic transfer of other factors induced by the transgenes.

Response: Thank you for this important comment. We agree that constructs expressing the same mRNAs without the mobility motif are essential controls for evaluating whether the observed phenotypic effects are associated with 2×GME2-mediated RNA mobility, rather than with systemic effects indirectly caused by transgene expression in donor tissues. To address this concern, we added the corresponding control grafts using transgenic lines expressing *AtMYB49*, *AtHMA3*, or *AtPCR2* without the 2×GME2 cassette. Phenotypic and quantitative analyses showed that graft combinations containing the mobile 2×GME2 cassette exhibited stronger stress-related phenotypic effects than the corresponding motif-minus controls under salt, Cd, or Zn stress. These results, together with the detection of the corresponding 2×GME2-tagged transcripts and encoded protein in recipient tissues, support the conclusion that 2×GME2 promotes the delivery of functional mRNAs across the graft junction and contributes to the associated phenotypic improvements in the grafted plants. We have revised the Discussion sections accordingly and included these additional control data in Figure. EV9.

3. -Missing information that should be added:

The DNA sequence of the 3WJ-4×Bro fused to 2×GME2 used in the study should be presented. Also it is not mentioned which TLS sequence was used for the comparison of mobility.

Response: Thank you for this helpful comment. We have now provided the DNA sequence of the 3WJ-4×Bro fused to 2×GME2 construct in the Reagents and Tools Table, which has been uploaded as a separate file during submission. We have also clarified the TLS sequence used for the mobility comparison.

Referee #2:

Thank you for the revised version of the manuscript. I have carefully reviewed the authors' responses and the changes made. The revision adequately addresses my previous comments, and I have no

further concerns.
I recommend that the manuscript be accepted in its current form.

Referee #3:

1. - The revised manuscript by Tao et al. is much improved. We list a few additional minor revisions below:

Lines 16-18. "Live imaging of 100 RNA Seq-predicted candidates identified only three bona fide mobile mRNAs (CAT3, CK1, and GAI), with GAI mRNA exhibiting the highest bidirectional mobility." This statement is too strong, as previously pointed out by reviewer 1 in their first comment. It would be better to use an alternative term instead of "bona fide", for example, "demonstrated mobile mRNAs".

Response： Thank you for this helpful suggestion. We agree that "bona fide" was too strong in this context. We have revised the Abstract to remove both "only" and "bona fide" and now describe *CAT3*, *CK1* and *GAI* as mobile mRNAs detected reproducibly under our assay conditions.

2. - Lines 23-24. "Together, these findings establish a live-imaging-guided pipeline for mobile RNA discovery." Discovery came from the original paper in which these 100 RNAs were identified; the term "validation" would be more appropriate.

Response： Thank you for your helpful suggestion. We agree that "discovery" was not appropriate in this context. We have therefore revised the sentence and changed "mobile RNA discovery" to "mobile RNA validation."

3. - Line 174. I think that hpi is hours post-infection, but you never clarify this in the text.

Response： Thank you for your comment. We apologize for any confusion. The abbreviation "hpi" has been defined as "hours post-inoculation" at its first occurrence in Line 94. We have checked the revised manuscript to ensure that this definition appears before "hpi" is used later in the text.

4. - Line 175-187; 207-208. Is this really 'velocity' or 'mobile rate'? Single-molecule imaging would be required to make statements about velocity. It would be better to use another term, such as mobile rate or mobility estimate. Please define your parameters for this estimate within the main text, and not just in the methods.

Response: Thank you for this important clarification. We agree that our assay does not measure single-molecule velocity. We have therefore revised the terminology to avoid implying single-molecule tracking and now refer to this parameter as an "transport rate," as appropriate. We define this operational parameter in the main text and figure legends as the distance from the inoculation

site to the designated distal region divided by the time after inoculation at which the first fluorescence-positive plant was detected in that region. We also define “mobility rate” as the percentage of plants showing detectable distal fluorescence among the total number examined.

5. - Lines 206-207: Is it possible to estimate how base pairs correspond with kD? That would be interesting.

Response： Thank you for the suggestion. Because the delivery module is evaluated here for RNA cargos rather than protein cargos, we describe cargo size in nucleotides, which more directly reflects the transported RNA molecule. We have clarified the RNA cargo size range in the revised text to make the delivery capacity easier to interpret.

6. - In Lines 209-211: The statement, "with full load delivery efficiency of approximately 35% (Fig. EV5B)" is unclear. Please define more clearly.

Response: Thank you for pointing this out. We agree that “full-load delivery efficiency” was unclear. We have replaced this phrase with a more explicit description and now define mobility rate as the percentage of plants showing detectable distal fluorescence among the total number examined. We also specify that the approximately 35% mobility rate refers to the 5×mCherry cargo. (Line 203-205) .

7. - Lines 264-266: It would be more accurate to add the following qualifier to this statement - "Within this framework, only three of the 100 candidates tested here showed reproducible long-distance movement" + "in our experimental conditions". This added statement covers potential conditional requirements for movement that may not be consistent across experimental settings.

Response： Thank you for your helpful suggestion. We have revised the statement accordingly by adding the qualifier “under our experimental conditions” to avoid over generalization. (Line 259)

2nd Revision - Editorial Decision 22nd May 2026

Yun Zhao
Sichuan University
College of Life Sciences
No.29 Wangjiang Road
Chengdu 610064
China

Dear Dr. Zhao,

I am very pleased to accept your manuscript for publication in the next available issue of EMBO reports. Thank you for your contribution to our journal.

Your manuscript will be processed for publication by EMBO Press. It will be copy edited and you will receive page proofs prior to publication. Please note that you will be contacted by Springer Nature Author Services to complete licensing and payment information.

You may qualify for financial assistance for your publication charges - either via a Springer Nature fully open access agreement or an EMBO initiative. Check your eligibility: https://link.springer.com/journal/44319/how-to-publish-with-us

Should you be planning a Press Release on your article, please notify the production team via this form in order to coordinate publication and release dates: https://forms.cloud.microsoft/e/eH4A8JVP6g

If you have any questions, please do not hesitate to contact the Editorial Office. Thank you for your contribution to EMBO Reports.

Best regards,
Esther

Esther Schnapp, PhD
Senior Editor
EMBO reports

>>> Please note that it is EMBO Reports policy for the transcript of the editorial process (containing referee reports and your response letter) to be published as an online supplement to each paper. If you do NOT want this, you will need to inform the Editorial Office via email immediately. More information is available here: https://link.springer.com/partners/embo-press/editorial-policies#Peer%20review

EMBO Press Author Checklist

Corresponding Author Name: Yun Zhao	
Journal Submitted to: EMBO Reports	
Manuscript Number: EMBOR-2025-63321V1	

USEFUL LINKS FOR COMPLETING THIS FORM
[The EMBO Journal - Author Guidelines]
[EMBO Reports - Author Guidelines]
[Molecular Systems Biology - Author Guidelines]
[EMBO Molecular Medicine - Author Guidelines]

Reporting Checklist for Life Science Articles (updated January
This checklist is adapted from Materials Design Analysis Reporting (MDAR) Checklist for Authors. MDAR establishes a minimum set of requirements in transparent reporting in the life sciences (see Statement of Task: 10.31222/osf.io/9sm4x). Please follow the journal's guidelines in preparing your
Please note that a copy of this checklist will be published alongside your article.

Abridged guidelines for figures
1. Data
The data shown in figures should satisfy the following conditions:
- the data were obtained and processed according to the field's best practice and are presented to reflect the results of the experiments in an accurate and unbiased manner.
- ideally, figure panels should include only measurements that are directly comparable to each other and obtained with the same assay.
- plots include clearly labeled error bars for independent experiments and sample sizes. Unless justified, error bars should not be shown for technical
- if n<5, the individual data points from each experiment should be plotted. Any statistical test employed should be justified.
- Source Data should be included to report the data underlying figures according to the guidelines set out in the authorship guidelines on Data

2. Captions
Each figure caption should contain the following information, for each panel where they are relevant:
- a specification of the experimental system investigated (eg cell line, species name).
- the assay(s) and method(s) used to carry out the reported observations and measurements.
- an explicit mention of the biological and chemical entity(ies) that are being measured.
- an explicit mention of the biological and chemical entity(ies) that are altered/varied/perturbed in a controlled manner.
- the exact sample size (n) for each experimental group/condition, given as a number, not a range;
- a description of the sample collection allowing the reader to understand whether the samples represent technical or biological replicates (including how many animals, litters, cultures, etc.).
- a statement of how many times the experiment shown was independently replicated in the laboratory.
- definitions of statistical methods and measures:
  - common tests, such as t-test (please specify whether paired vs. unpaired), simple $\chi 2$ tests, Wilcoxon and Mann-Whitney tests, can be unambiguously identified by name only, but more complex techniques should be described in the methods section;
  - are tests one-sided or two-sided?
  - are there adjustments for multiple comparisons?
  - exact statistical test results, e.g., P values = x but not P values < x;
  - definition of 'center values' as median or average;
  - definition of error bars as s.d. or s.e.m.

Please complete ALL of the questions below.
Select "Not Applicable" only when the requested information is not relevant for your study.

Materials

Newly Created Materials	Information included in the manuscript?	In which section is the information available? (Reagents and Tools Table, Materials and Methods, Figures, Data Availability Section)
New materials and reagents need to be available; do any restrictions apply?	Not Applicable	

Antibodies	Information included in the manuscript?	In which section is the information available? (Reagents and Tools Table, Materials and Methods, Figures, Data Availability Section)
For **antibodies** provide the following information:<br>- Commercial antibodies: RRID (if possible) or supplier name, catalogue number and or/clone number<br>- Non-commercial: RRID or citation	Not Applicable	

DNA and RNA sequences	Information included in the manuscript?	In which section is the information available? (Reagents and Tools Table, Materials and Methods, Figures, Data Availability Section)
**Short novel DNA or RNA including primers, probes**: provide the sequences.	Yes	Reagents and Tools Table, Materials and Methods

Cell materials	Information included in the manuscript?	In which section is the information available? (Reagents and Tools Table, Materials and Methods, Figures, Data Availability Section)
**Cell lines:** Provide species information, strain. Provide accession number in repository **OR** supplier name, catalog number, clone number, and/**OR** RRID.	Not Applicable	
**Primary cultures:** Provide species, strain, sex of origin, genetic modification status.	Not Applicable	
Report if the cell lines were recently **authenticated** (e.g., by STR profiling) and tested for mycoplasma contamination.	Not Applicable	

Experimental animals	Information included in the manuscript?	In which section is the information available? (Reagents and Tools Table, Materials and Methods, Figures, Data Availability Section)
**Laboratory animals or Model organisms:** Provide species, strain, sex, age, genetic modification status. Provide accession number in repository **OR** supplier name, catalog number, clone number, **OR** RRID.	Not Applicable	
**Animal observed in or captured from the field:** Provide species, sex, and age where possible.	Not Applicable	
Please detail **housing and husbandry conditions**.	Not Applicable	

Plants and microbes	Information included in the manuscript?	In which section is the information available? (Reagents and Tools Table, Materials and Methods, Figures, Data Availability Section)
**Plants:** provide species and strain, ecotype and cultivar where relevant, unique accession number if available, and source (including location for collected wild specimens).	Yes	Reagents and Tools Table, Materials and Methods
**Microbes:** provide species and strain, unique accession number if available, and source.	Yes	Reagents and Tools Table, Materials and Methods

Human research participants	Information included in the manuscript?	In which section is the information available? (Reagents and Tools Table, Materials and Methods, Figures, Data Availability Section)
If collected and within the bounds of privacy constraints report on age, sex and gender or ethnicity for all study participants.	Not Applicable	

Core facilities	Information included in the manuscript?	In which section is the information available? (Reagents and Tools Table, Materials and Methods, Figures, Data Availability Section)
If your work benefited from core facilities, was their service mentioned in the acknowledgments section?	Not Applicable	

Design

Study protocol	Information included in the manuscript?	In which section is the information available? (Reagents and Tools Table, Materials and Methods, Figures, Data Availability Section)
If study protocol has been **pre-registered, provide DOI in the manuscript**. For clinical trials, provide the trial registration number **OR** cite DOI.	Not Applicable	
Report the **clinical trial registration number** (at ClinicalTrials.gov or equivalent), where applicable.	Not Applicable	

Laboratory protocol	Information included in the manuscript?	In which section is the information available? (Reagents and Tools Table, Materials and Methods, Figures, Data Availability Section)
Provide DOI OR other citation details if **external detailed step-by-step protocols** are available.	Yes	Materials and Methods

Experimental study design and statistics	Information included in the manuscript?	In which section is the information available? (Reagents and Tools Table, Materials and Methods, Figures, Data Availability Section)
Include a statement about **sample size** estimate even if no statistical methods were used.	Not Applicable	
Were any steps taken to minimize the effects of subjective bias when allocating animals/samples to treatment (e.g. **randomization procedure**)? If yes, have they been described?	Not Applicable	
Include a statement about **blinding** even if no blinding was done.	Not Applicable	
Describe **inclusion/exclusion criteria** if samples or animals were excluded from the analysis. Were the criteria pre-established?<br><br>If sample or data points were omitted from analysis, report if this was due to attrition or intentional exclusion and provide justification.	Not Applicable	
For every figure, are **statistical tests** justified as appropriate? Do the data meet the assumptions of the tests (e.g., normal distribution)? Describe any methods used to assess it. Is there an estimate of variation within each group of data? Is the variance similar between the groups that are being statistically compared?	Yes	Figures

Sample definition and in-laboratory replication	Information included in the manuscript?	In which section is the information available? (Reagents and Tools Table, Materials and Methods, Figures, Data Availability Section)
In the figure legends: state number of times the experiment was **replicated** in laboratory.	Yes	Figures
In the figure legends: define whether data describe **technical or biological replicates**.	Yes	Figures

Ethics

Ethics	Information included in the manuscript?	In which section is the information available? (Reagents and Tools Table, Materials and Methods, Figures, Data Availability Section)
Studies involving **human participants**: State details of **authority granting ethics approval** (IRB or equivalent committee(s), provide reference number for approval.	Not Applicable	
Studies involving **human participants**: Include a statement confirming that **informed consent** was obtained from all subjects and that the experiments conformed to the principles set out in the WMA Declaration of Helsinki and the Department of Health and Human Services Belmont Report.	Not Applicable	
Studies involving **human participants:** For publication of **patient photos**, include a statement confirming that consent to publish was obtained.	Not Applicable	
Studies involving experimental **animals**: State details of **authority granting ethics approval** (IRB or equivalent committee(s), provide reference number for approval. Include a statement of compliance with ethical regulations.	Not Applicable	
Studies involving **specimen and field samples:** State if relevant **permits** obtained, provide details of authority approving study; if none were required, explain why.	Not Applicable	

Dual Use Research of Concern (DURC)	Information included in the manuscript?	In which section is the information available? (Reagents and Tools Table, Materials and Methods, Figures, Data Availability Section)
Could your study fall under dual use research restrictions? Please check biosecurity documents and list of **select agents and toxins** (CDC): https://www.selectagents.gov/sat/list.htm	Not Applicable	
If you used a select agent, is the security level of the lab appropriate and reported in the manuscript?	Not Applicable	
If a study is subject to dual use research of concern regulations, is the name of the **authority granting approval and reference number** for the regulatory approval provided in the manuscript?	Not Applicable	

Reporting

The MDAR framework recommends adoption of discipline-specific guidelines, established and endorsed through community initiatives. Journals have their own policy about requiring specific guidelines and recommendations to complement MDAR.

Adherence to community standards	Information included in the manuscript?	In which section is the information available? (Reagents and Tools Table, Materials and Methods, Figures, Data Availability Section)
State if relevant guidelines or checklists (e.g., **ICMJE, MIBBI, ARRIVE, PRISMA**) have been followed or provided.	Not Applicable	
For **tumor marker prognostic studies,** we recommend that you follow the **REMARK** reporting guidelines (see link list at top right). See author guidelines, under 'Reporting Guidelines'. Please confirm you have followed these guidelines.	Not Applicable	
For **phase II and III randomized controlled trials**, please refer to the **CONSORT** flow diagram (see link list at top right) and submit the CONSORT checklist (see link list at top right) with your submission. See author guidelines, under 'Reporting Guidelines'. Please confirm you have submitted this list.	Not Applicable	

Data Availability

Data availability	Information included in the manuscript?	In which section is the information available? (Reagents and Tools Table, Materials and Methods, Figures, Data Availability Section)
Have **primary datasets** been deposited according to the journal's guidelines (see 'Data Deposition' section) and the respective accession numbers provided in the Data Availability Section?	Yes	Data Availability Section
Were **human clinical and genomic datasets** deposited in a public access-controlled repository in accordance to ethical obligations to the patients and to the applicable consent agreement?	Not Applicable	
Are **computational models** that are central and integral to a study available without restrictions in a machine-readable form? Were the relevant accession numbers or links provided?	Not Applicable	
If publicly available data were reused, provide the respective **data citations in the reference list**.	Yes	Results
